# Higher‐order modular regulation of the human proteome

**DOI:** 10.15252/msb.20209503

**Published:** 2023-03-09

**Authors:** Georg Kustatscher, Martina Hödl, Edward Rullmann, Piotr Grabowski, Emmanuel Fiagbedzi, Anja Groth, Juri Rappsilber

**Affiliations:** ^1^ Wellcome Centre for Cell Biology University of Edinburgh Edinburgh UK; ^2^ Biotech Research and Innovation Centre (BRIC), Faculty of Health and Medical Sciences University of Copenhagen Copenhagen Denmark; ^3^ Bioanalytics, Institute of Biotechnology Technische Universität Berlin Berlin Germany; ^4^ Data Sciences and Artificial Intelligence, Clinical Pharmacology & Safety Sciences, IMED Biotech Unit AstraZeneca Cambridge UK; ^5^ Novo Nordisk Foundation Center for Protein Research (CPR), Faculty of Health and Medical Sciences University of Copenhagen Copenhagen Denmark; ^6^ Present address: Austrian Academy of Sciences Vienna Austria

**Keywords:** DNA replication, machine‐learning, mRNA coexpression, protein co‐regulation, quantitative proteomics, Computational Biology, Methods & Resources, Proteomics

## Abstract

Operons are transcriptional modules that allow bacteria to adapt to environmental changes by coordinately expressing the relevant set of genes. In humans, biological pathways and their regulation are more complex. If and how human cells coordinate the expression of entire biological processes is unclear. Here, we capture 31 higher‐order co‐regulation modules, which we term progulons, by help of supervised machine‐learning on proteomics data. Progulons consist of dozens to hundreds of proteins that together mediate core cellular functions. They are not restricted to physical interactions or co‐localisation. Progulon abundance changes are primarily controlled at the level of protein synthesis and degradation. Implemented as a web app at www.proteomehd.net/progulonFinder, our approach enables the targeted search for progulons of specific cellular processes. We use it to identify a DNA replication progulon and reveal multiple new replication factors, validated by extensive phenotyping of siRNA‐induced knockdowns. Progulons provide a new entry point into the molecular understanding of biological processes.

## Graphical abstract

Clustering is combined with machine‐learning to identify large modules of co‐regulated proteins in the human proteome. A simple web‐based implementation of the workflow allows users to search for additional co‐regulation modules.

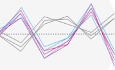

## Introduction

More than 50 years ago, the discovery of the *lac* operon launched the field of gene regulation (Jacob & Monod, 1961; Jacob, 2011). Tightly linked to the discovery of mRNA (Cobb, 2015), the operon concept provided a model for how genes are turned on and off. It also demonstrated how organisms adapt to changes in their environment: not by transforming one enzyme into another as was believed at the time, but by regulating gene expression (Lewis, 2011; Loison, 2013). Hundreds of operons have since been mapped in bacteria, archaea and in some eukaryotes (Blumenthal, 2004). In the classical sense, an operon contains a set of adjacent genes involved in the same metabolic pathway, whose transcription into a polycistronic mRNA is controlled by a shared regulator. It is both a transcriptional unit and a functional module. However, eukaryotic operons differ from this definition in a crucial aspect. For example, 15% of *Caenorhabditis elegans* genes are arranged in operons of 2–8 genes, but these tend to be housekeeping genes rather than inducible ones (Morton & Blumenthal, 2011). Moreover, genes in a single operon do not usually have related functions (Morton & Blumenthal, 2011). Similarly, dicistronic transcripts are “mini‐operons” found in many animals and plants that can have metabolically related functions, but often do not (Blumenthal, 2004; Thimmapuram *et al*, 2005). The most striking divergence between co‐transcription and co‐function is found in trypanosomes, which transcribe an entire chromosome into two large polycistronic transcripts (Martínez‐Calvillo *et al*, 2003). In humans, dicistronic transcripts are rare, but divergently transcribed (bidirectional) gene pairs account for more than 10% of protein‐coding genes (Trinklein *et al*, 2004). Similar to nematode operons, these are co‐transcribed from a shared promoter region (Trinklein *et al*, 2004), tend to have housekeeping activities (Lercher *et al*, 2002; Xu *et al*, 2012), but are rarely functionally related (Xu *et al*, 2012). Indeed, a substantial proportion of human transcript coexpression does not reflect shared function, but gene proximity in sequence or 3D structure of the genome (Batada *et al*, 2007; Ebisuya *et al*, 2008; Kustatscher *et al*, 2017; Wang *et al*, 2017), or genetic (Geiger *et al*, 2010; Stingele *et al*, 2012; Khan *et al*, 2013; Battle *et al*, 2015) and epigenetic (Raj *et al*, 2006; Batada *et al*, 2007; Gandhi *et al*, 2011; Grabowski *et al*, 2018) variation. Importantly, post‐transcriptional and post‐translational regulation ensures that non‐functional mRNA coexpression is not propagated to the protein level (Geiger *et al*, 2010; Stingele *et al*, 2012; Khan *et al*, 2013; Battle *et al*, 2015; Kustatscher *et al*, 2017; Wang *et al*, 2017; Grabowski *et al*, 2018). Based on such observations, it has been suggested that co‐transcription in eukaryotes is not a mechanism for the coordinated regulation of gene modules, but rather a way to ensure efficient, universal expression of housekeeping genes (Batada & Hurst, 2007; Morton & Blumenthal, 2011; Wang *et al*, 2011; Kustatscher *et al*, 2017). Consequently, it remains unclear whether human cells adapt to changes in their environment by regulating the expression of genuine “functional modules,” and if so, how we can identify and characterise them.

Here, we address this question by analysing human protein co‐regulation with machine‐learning. This strategy differs in two key points from traditional gene coexpression studies. First, to ensure we capture functionally relevant expression changes, we analyse protein rather than mRNA abundances. Second, rather than using correlation networks, we analyse the data with a supervised machine‐learning approach. Together, this allowed us to capture 31 large co‐regulation modules, each consisting of dozens to hundreds of proteins, which accurately and comprehensively reflect biological processes regulated in human cells. In reference to bacterial regulons, which are functional but not transcriptional units of regulation (Maas, 1964), we term these modules progulons (protein regulons). The modular nature of human protein expression control can be exploited to identify new proteins contributing to important cellular processes, as we demonstrate at the example of DNA replication. Through our website https://www.proteomehd.net/progulonFinder, biologists can expand the known boundaries of a biological process of interest, by executing our machine‐learning workflow with a single click.

## Results

### Systematic identification of co‐regulated protein modules

To find out whether human cells coordinate the expression of functionally related proteins at a larger scale than currently known, we resorted to our recently reported ProteomeHD, a data matrix documenting the up‐ or downregulation of 10,323 human proteins in response to 294 biological perturbations, including treatments with drugs, growth factors and comparisons of cancer cell lines (Kustatscher *et al*, 2019). Protein abundance changes were measured with high quantitative accuracy using SILAC‐labelling mass spectrometry (Ong *et al*, 2002). In principle, co‐regulation modules in ProteomeHD could be detected through correlation network analysis and clustering (Zhang & Horvath, 2005; Wu *et al*, 2013; Wilhelm *et al*, 2014). However, protein expression control in human cells is very complex and dynamic: proteins may work together in some conditions or biological processes but not in others, and many proteins may only respond to a subset of perturbations. In such complex data, correlation analyses tend to identify only the most strongly and ubiquitously co‐regulated proteins (Montaño‐Gutierrez *et al*, 2017). By contrast, we have previously shown that Random Forests (RF; Breiman, 2001), due to their intrinsic feature selection and outlier robustness, capture co‐regulation patterns in proteomics data very well (Kustatscher *et al*, 2014, 2016). As a supervised machine‐learning approach, the RF algorithm creates a classifier that specifically detects proteins which are co‐regulated with a given set of training proteins. In this way, co‐regulation analysis can be focussed on a specific set of proteins with high accuracy and sensitivity, allowing for a more powerful detection of co‐regulated proteins compared with (unsupervised) clustering approaches. However, the requirement of providing training data means that RFs cannot detect co‐regulation modules *de novo*, that is without prior knowledge of at least some components of these modules, even if training sets can be as small as individual protein complexes (Montaño‐Gutierrez *et al*, 2017).

**Figure 1:**
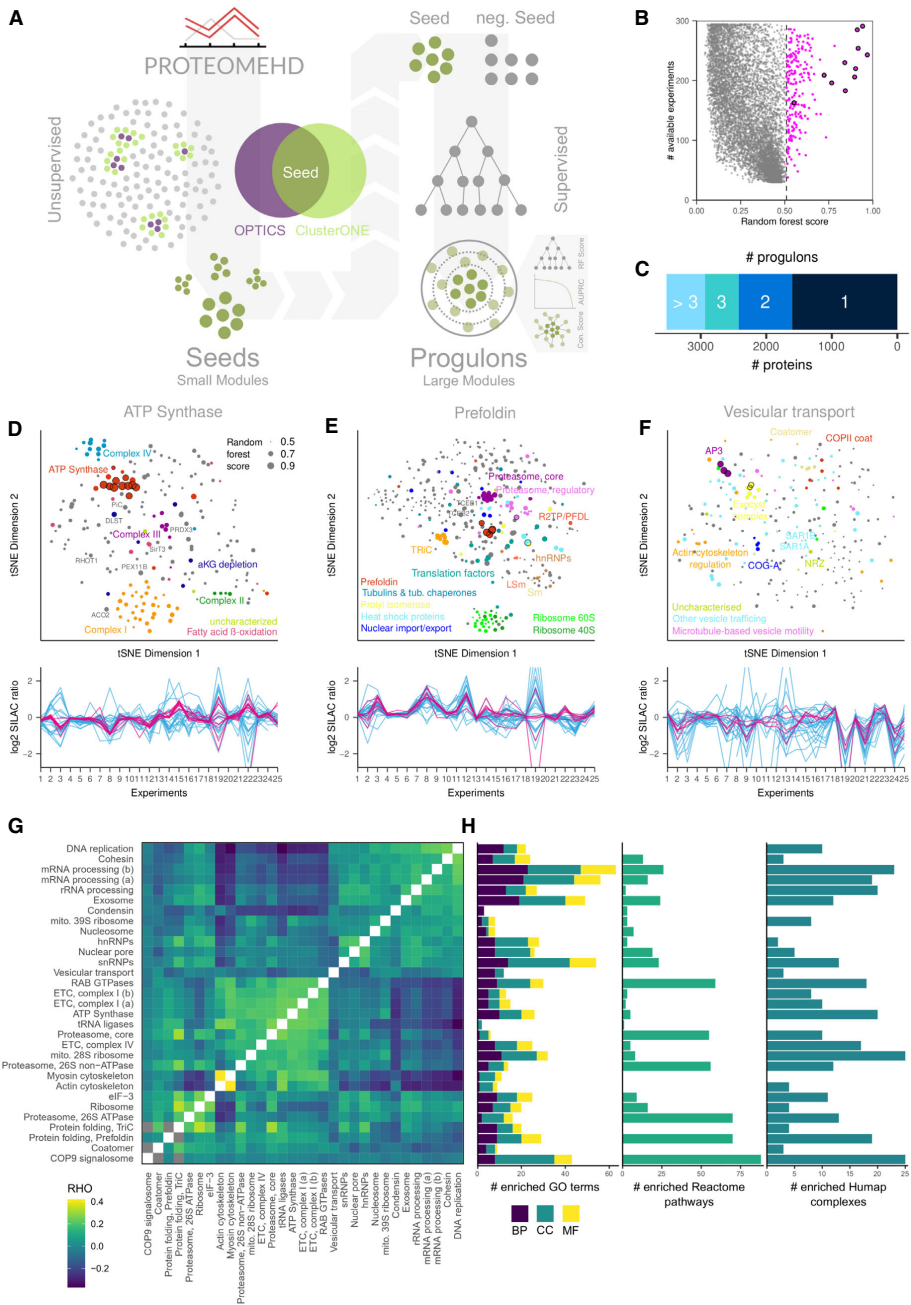
Protein co‐regulation modules capture comprehensive cellular processes A
Outline. Clustering identifies small groups of proteins (“seeds”) that are tightly co‐regulated in response to perturbations in ProteomeHD. A Random Forest‐based machine‐learning workflow, progulonFinder, subsequently captures large protein regulons (progulons) matching the regulatory patterns defined by these seed proteins.B
Example result for a seed containing 11 subunits of the ATP synthase complex (black circles). progulonFinder returns 193 proteins that are co‐regulated with the ATP synthase (magenta).C
Barchart showing how many proteins have been assigned to how many progulons.D–F
(D) t‐SNE map of the ATP synthase progulon, where the distance between proteins indicates how similar their perturbation responses are across ProteomeHD. Dot size shows strength of co‐regulation with the seed proteins (circled). The map is completely data‐driven, labels are only added for illustration. Line plot shows up‐ and downregulation of the seed proteins (magenta) across 25 representative experiments from ProteomeHD. The top 25 co‐regulated progulon proteins are shown in blue. (E, F) Progulons related to prefoldin‐based protein folding and vesicular transport, respectively.G
Overview of the 31 progulons, named after their key biological process. Heatmap shows the average coexpression (Spearman's rank correlation) between the proteins in each progulon. Progulons are clustered by expression similarity.H
Number of Gene Ontology terms, Reactome pathways and HuMap complexes enriched in each progulon.

Here, we combine the advantages of clustering and supervised machine‐learning to identify large co‐regulation modules (progulons) in a systematic way. For this, we developed a two‐stage approach (Fig 1A). First, we use clustering to detect small, tightly co‐regulated protein modules. Next, we use these clusters as “seeds,” or training proteins, for the Random Forests algorithm to detect much larger co‐regulation modules.

### Clustering identifies small, compact co‐regulation modules

We tested three types of clustering approaches for their ability to identify biologically meaningful clusters in ProteomeHD. Hierarchical clustering performed relatively poorly. For example, depending on the cluster calling cut‐off, subunits of the ATP synthase complex were either spread across multiple small clusters or part of a single big cluster that also contained many unrelated proteins (Appendix Fig S1). By contrast, we found density‐based clustering using OPTICS (Ankerst *et al*, 1999) and graph clustering using clusterONE (Nepusz *et al*, 2012) to be better suited for ProteomeHD data. OPTICS and clusterONE produced similar outcomes, despite being based on different mathematical principles and using different input formats (whole ProteomeHD protein–protein association matrix and network of the top 0.5% associations, respectively). For each OPTICS cluster, we identified the clusterONE cluster with the most overlapping proteins and discarded proteins that were only assigned to the cluster by one of the two algorithms. The resulting 72 core modules contained an average of eight proteins (range: 4–81), which were identified as clustered by both types of clustering approaches (Dataset EV1). Many of these small modules correspond to protein complexes. For example, one consists of 11 ATP synthase subunits (out of 18).

### progulonFinder identifies large co‐regulation modules

The 72 core modules detected by clustering were used as “seeds” to identify larger co‐regulation modules by supervised machine‐learning. For this, we developed progulonFinder, a framework for fully automated RF analysis of ProteomeHD (Appendix Fig S2). Starting from a list of seed proteins, progulonFinder trains, tests and averages multiple balanced RF models, performs cross‐validation and outputs the progulon, that is a list of proteins classified as co‐regulated with the seed proteins (see Materials and Methods).

We designed several stringent filtering criteria to ensure the high quality of the final list of progulons. This included a requirement for the cross‐validated training data to achieve a ROC curve area above 0.99 and a requirement that at least four of the 10 proteins with the highest RF scores had to be cross‐validated training proteins. Finally, we introduced a requirement for progulons to be genuine co‐regulation modules, that is a group of proteins that are not only co‐regulated with the seed proteins but also with each other. This was achieved by calculating a “connectivity score”: we compare progulons to a co‐regulation network created with all proteins in the analysis and, using a Fisher's exact test, calculate a P‐value that reflects if a progulon is enriched for protein pairs that are among the top 0.5% of co‐regulated protein pairs in the overall network. We then chose the minimum RF score cut‐off that creates a significantly interconnected module (see Materials and Methods). Only 31 of the 72 seed modules produced a progulon that passed all of our quality control filters (Dataset EV2).

For example, progulonFinder identifies 193 proteins that match the expression pattern of the 11 ATP synthase subunits (Fig 1B). We visualise this “ATP synthesis progulon” using t‐Distributed Stochastic Neighbour Embedding (t‐SNE) (Van Der Maaten & Hinton, 2008), which displays the expression similarities between the progulon proteins in two dimensions (Fig 1D). The progulon contains a range of proteins and protein complexes that are directly or indirectly associated with ATP synthesis (see below). Another progulon is seeded by the prefoldin chaperone and shows the ribosome‐associated folding machinery for tubulin and other proteins (Fig 1E). Finally, a vesicle‐trafficking progulon is revealed by a seed containing the AP3 and exocyst complexes, retrieving additional complexes involved in endocytic vesicle transport (Fig 1F). The strength of co‐regulation with the seed proteins differs between progulons (Fig 1D–F, line plots).

On average, progulons consist of 246 proteins, ranging from 13 to 1,143. A total of 3,523 proteins have been assigned to at least one progulon (Fig 1C). Although the 31 seed groups are nonredundant, there is some overlap between progulons that were seeded by functionally similar proteins, such as the two progulons seeded by a different set of mRNA processing factors (Appendix Fig S3). However, even after excluding any overlapping proteins, expression changes in functionally related progulons can be strongly correlated, for example for the myosin and actin cytoskeleton progulons (Fig 1G). Importantly, we find that all progulons are significantly enriched in Gene Ontology (GO) terms, and most progulons are also enriched for one or more Reactome pathways and known protein complexes (Fig 1H).

As a control experiment, progulonFinder was executed using a set of random seed proteins, matching the real ones in number and size distribution. None of these random seeds produced a progulon that passed our quality criteria, suggesting that our quality criteria were very strict. In addition, for a random grouping of proteins that matched the progulons in number and size distributions, we found no significant enrichment in either GO terms, Reactome pathways or protein complexes (Appendix Fig S3).

### Close‐up of a well‐characterised progulon: ATP Synthesis

**Figure EV1:**
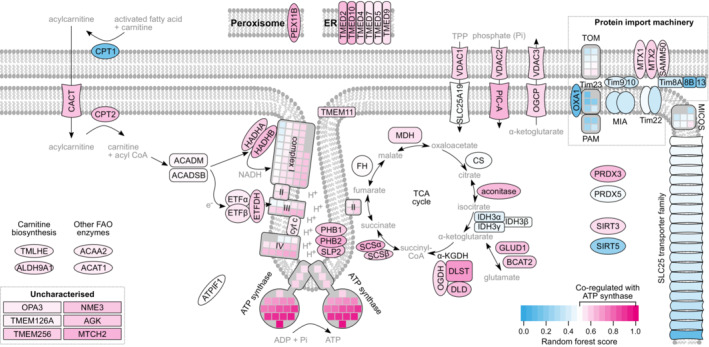
Outline of the ATP synthase progulon Drawing of ATP synthase related biological processes, colour‐coded according to how strongly each protein is co‐regulated with the ATP synthase. This includes almost every protein of the electron transport chain (complexes I‐IV) and the fatty acid β‐oxidation (FAO) pathway except its rate‐limiting enzyme CPT1. This suggests that up‐ or downregulation of the ATP synthase is generally accompanied by a corresponding change in the pathways building up the proton gradient. About 60% of proteins in this progulon have a known function that is clearly linked to ATP synthesis and these are shown here. See Dataset EV3 for a full protein list. The protein that most closely matches the ATP synthase expression pattern, DLST, is part of the TCA cycle in the mitochondrial matrix. As a subunit of the α‐ketoglutarate dehydrogenase, DLST depletes the endogenous ATP synthase inhibitor α‐ketoglutarate (α‐KG) (Chin *et al*, 2014). Three other top hits either metabolise (GLUD1, BCAT2) or export α‐KG (OGCP). By contrast, isocitrate dehydrogenase, which generates α‐KG, is a notable absence among the TCA cycle enzymes, suggesting that part of the biological significance of this progulon may be to prevent metabolic inhibition of ATP synthesis. A third function of the progulon may be to reduce the impact of reactive oxygen species (ROS), which are by‐products of ATP synthesis. For example, among the strongest co‐regulation partners of the ATP synthase are the two most ROS‐sensitive enzymes of the TCA cycle, DLST and aconitase, both of which can be readily inactivated by oxidative damage. Coordinating their expression with the respirasome may be a way to ensure flux through the cycle even in the presence of oxidative stress. Other high‐scoring proteins include the antioxidant peroxiredoxin III and PEX11B, which creates peroxisome‐mitochondria connections thought to alleviate oxidative stress on mitochondria (Kustatscher *et al*, 2019). Control proteins that localise to the inner membrane but are not directly related to ATP synthesis are absent from the progulon. This includes the MICOS complex, the protein import machinery and the bulk of the SLC25 transporter family (some SLC25 proteins have ATP synthesis‐related functions, for example PiC‐A imports the substrate inorganic phosphate).

Co‐regulation in response to biological perturbations indicates a functional link between proteins, but it does not pinpoint the molecular nature of the link. In fact, we have previously shown that protein co‐regulation captures a wide range of associations, from physical protein–protein interactions to metabolic and other functional associations (Kustatscher *et al*, 2019). While this presents a challenge when following up on the mechanism of novel links, it allows for a very comprehensive detection of functional interactions. To illustrate this, we annotated the ATP synthesis progulon in detail, making use of the extensive prior knowledge available for this biological process (Fig EV1). Most proteins in the progulon have well‐defined roles related to ATP production, including dozens of proteins that interact with the ATP synthase to form the respirasome in the inner mitochondrial membrane (Wu *et al*, 2016; Dataset EV3). Beyond these physical associations, our data suggest that many matrix proteins are co‐regulated with the ATP synthase to prevent the accumulation of its metabolic inhibitor, α‐ketoglutarate (Chin *et al*, 2014), and that of reactive oxygen species (Turrens, 2003). Finally, we used the SLC25 transporter family to assess the specificity of our approach. SLC25 proteins also localise to the inner mitochondrial membrane but are involved in a variety of distinct mitochondrial processes. Reassuringly, only a fraction of SLC25 proteins were assigned to the progulon and these have known functions related to ATP production. For example, SLC25A3 imports inorganic phosphate (Seifert *et al*, 2015), an ATP synthase substrate (Fig EV1).

### Progulon expression control: at mRNA or protein level?

**Figure EV2:**
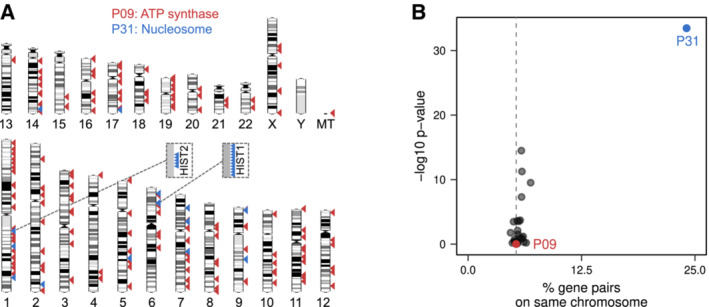
Progulons are not linked to gene position Human chromosomes showing the location of the genes involved in the ATP synthase (red) and Nucleosome (blue) progulons. HIST1 and HIST2 are two histone gene clusters on chromosomes 1 and 6, respectively.Except for this nucleosome progulon, progulons are not strongly enriched for genes from the same chromosome. A dashed line indicates the 5.3% of gene pairs that would be expected to be on the same chromosome by random chance; *P*‐values are from a two‐sided Fisher's exact test.

In contrast to bacterial and eukaryotic operons, we find no substantial clustering of progulon genes in terms of chromosome location. The ATP synthase progulon, for example, contains genes from all human autosomes and the mitochondrial genome (Fig EV2). The “nucleosome” progulon, which is partially encoded by histone gene clusters, is an exception (Fig EV2). This raises the question of which gene expression stage is responsible for coordinating progulon abundance changes. Are progulons controlled at the mRNA level through coordinated transcription and mRNA degradation—or at the protein level, via protein synthesis and degradation?

**Figure 2:**
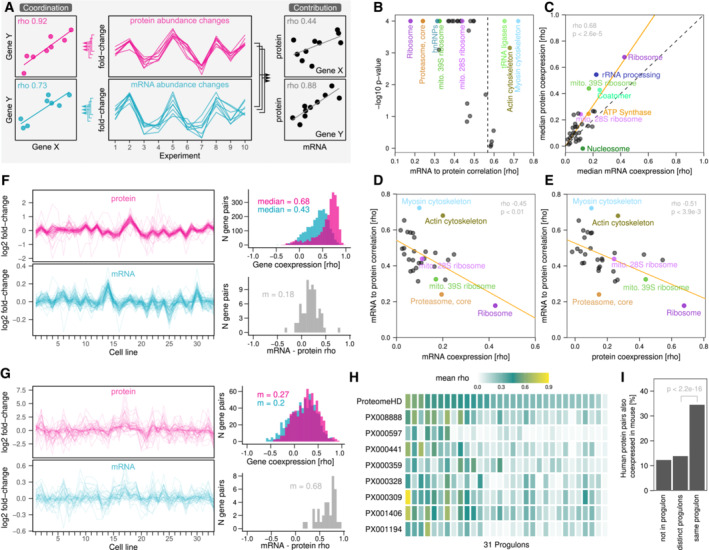
Regulation of progulon expression and coordination A
Illustration of two different aspects of progulon expression control: The coordination of progulon abundance changes (mRNA—mRNA; protein—protein) and the contribution of mRNA to protein abundance changes (mRNA‐protein).B
Spearman's correlation coefficients (rho) between mRNA and protein abundance changes across a breast cancer cell line panel. The median rho of all genes in the dataset is 0.57 (dashed line), but the median rho of genes assigned to different progulons can deviate significantly from that (*P*‐values from permutation testing). High and low mRNA‐to‐protein rho indicates regulation predominantly at the mRNA and protein level, respectively.C
Protein coordination (median protein–protein rho) increases with the mRNA coordination (orange regression line). Most progulons are located on the upper side of the diagonal dashed line, suggesting that they are better coordinated at the protein level.D, E
The mRNA‐to‐protein contribution is inversely correlated (rho, orange regression line) with both mRNA and protein coordination.F
Fold‐changes of the ribosome progulon across the 36 cancer cell lines and the corresponding correlations. It has strong mRNA and protein coordination (rho) (upper histogram) but poor mRNA‐to‐protein correlation (rho) (grey histogram).G
Same as (F), but for the “actin cytoskeleton” progulon, which has weak mRNA and protein coordination (rho) but strong mRNA‐to‐protein correlation (rho).H
Mean protein–protein correlations (rho) for each progulon calculated separately for subsets of ProteomeHD, namely those eight projects consisting of 10 or more individual experiments each. This plot indicates that progulon coordination can vary between cell lines and conditions.I
Co‐regulation of protein pairs in ProteomeHD, defined as rho > 0.5, is about twice as likely to be conserved across mouse tissues for proteins that are part of the same progulon. (*P* < 2.2e^−16^, Fisher's exact test)

To address this question, we analysed gene expression changes across 36 breast cancer cell lines that reflect the difference between breast cancer subtypes. Proteomics (Lapek *et al*, 2017) and transcriptomics (Klijn *et al*, 2015) data for these samples had been reported previously but were not included in ProteomeHD. Notably, there are two different aspects of expression control that are relevant for gene modules: the *contribution* of mRNA to protein abundance changes and the *coordination* of expression changes (Fig 2A). First, we calculated the Spearman's correlation coefficient (rho) between the mRNA and protein abundance changes of each gene. The median mRNA‐to‐protein rho is 0.57, higher than that reported for similar datasets (Fortelny *et al*, 2017; Appendix Fig S4A). We then asked if progulons differ from each other or the rest of the proteome. Indeed, three progulons are significantly enriched for high mRNA‐to‐protein rho (*P* < 0.001 in Monte‐Carlo permutation tests), indicating that transcription contributes more strongly to their regulation than for the proteome overall (Fig 2B). These include the actin and myosin cytoskeleton progulons as well as a tRNA ligase progulon. By contrast, 18 progulons are significantly enriched for low mRNA‐to‐protein rho (*P* = 9.9e^−05^), suggesting that their regulation is predominantly at the protein level (Fig 2B; Dataset EV4).

It has been suggested that the relative contribution of transcriptional and post‐transcriptional or post‐translational regulation depends on the amplitude of expression changes (Vogel & Marcotte, 2012). Indeed, we find a strong correlation between the scale of variation of progulon abundance across the breast cancer samples and the contribution of mRNA to protein abundance changes (Appendix Fig S4B and C; Spearman's rho 0.8).

Next, we assessed the *coordination* of progulon expression, correlating either mRNA or protein abundance changes for all gene pairs in a progulon (Fig 2A). In general, progulons that are more tightly co‐regulated at the protein level also tend to have better coordinated mRNA abundances (Fig 2C; Dataset EV4). We then tested at which stage expression changes are coordinated, expecting that this directly depends on the principal mechanism by which a progulon is controlled. For example, progulons with tightly coordinated mRNA abundances could be transcriptionally controlled, as they would produce coordinated protein changes without additional post‐transcriptional regulation. Surprisingly, we find that this is not the case: as the coordination of mRNA or protein expression changes becomes stronger, the contribution of mRNA to protein changes becomes weaker (Fig 2D and E, rho −0.45 and −0.51, respectively). This trend is driven by the ribosome and proteasome progulons in particular, but persists in weakened form even if these two progulons are removed (rho −0.36 and −0.46), at least across this breast cancer cell line dataset. The “ribosome” progulon shows the strongest mRNA covariation and the strongest protein covariation of any progulon—but the weakest mRNA‐to‐protein correlation. This means that both mRNA and protein abundance changes are highly coordinated, but independently and differently to each other (Fig 2F). By contrast, the “Actin cytoskeleton” progulon is more weakly coordinated at mRNA and protein level but shows one of the highest mRNA‐to‐protein correlations (Fig 2G). Moreover, there is no significant correlation between the amplitude of expression changes and the extent of progulon coordination (Appendix Fig S4D and E). Therefore, large expression changes are not a prerequisite for precise coordination of either mRNA or protein abundances. In short, we find that the *coordination* of mRNA abundances is distinct from the *contribution* of these mRNAs to the actual up‐ or downregulation of the progulon proteins. Similarly to progulons, we observe that also for protein complexes, there is a positive correlation between mRNA and protein coordination, and a weak but significant negative correlation between mRNA‐to‐protein contribution and mRNA coordination and protein coordination, respectively (Appendix Fig S4F–H).

In order to validate these results, we analysed two additional datasets with matched mRNA and protein measurements: a lymphoblastoid cell line panel, in which gene expression differs due to genetic variation, and mouse tissues, that is developmentally regulated expression changes. Overall, mRNA and protein levels correlate less well in these two datasets (median rho 0.17 and 0.43, respectively). These datasets generally confirmed the above conclusions (Appendix Figs S5 and S6). In these datasets, the negative correlation between *coordination* and *contribution* of expression changes is not significant. Nevertheless, this confirms that these are two separate aspects of gene regulation that are not directly, positively correlated as one might have expected.

We also observed that the mean protein rho can differ substantially within progulons when analysed separately for individual projects that contribute to ProteomeHD (Fig 2H). This suggests that some of these modules are more strongly co‐regulated in certain cell types and experimental conditions than in others.

### Correlated mRNA and protein half‐lives in progulons

**Figure EV3:**
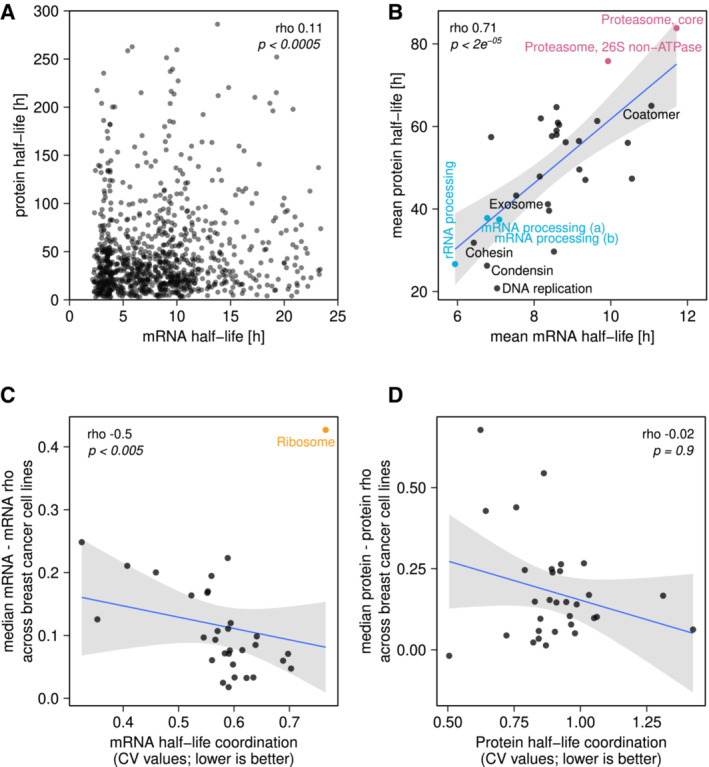
mRNA and protein half‐lives of progulons The mRNA and protein half‐lives of individual genes are correlated only very weakly.The average half‐life of all proteins and mRNAs of a progulon show a strong and significant correlation. This is even though mRNA half‐lives were measured in HeLa cells (Tani *et al*, 2012) and protein half‐lives in RPE1 cells (McShane *et al*, 2016). Note that proteins are longer lived than mRNAs.The coordination of mRNA half‐lives within progulons correlates with the degree of coordination of mRNA expression changes across the breast cancer cell line panel.No equivalent significant relationship is observed on the protein level.

We tested whether progulon coordination could be linked to mRNA or protein stability. Average mRNA half‐lives (Tani *et al*, 2012) of progulons range between 5 and 14 h, shorter than the respective protein half‐lives (McShane *et al*, 2016) ranging between 21 and 84 h (Dataset EV5). As reported by others (Schwanhäusser *et al*, 2011), we observe that the mRNA and protein half‐lives of individual genes are poorly correlated (Fig EV3A). By contrast, the average mRNA and protein half‐lives of progulons are strongly correlated (Fig EV3, rho 0.71, *P* < 2e^−5^). RNA processing and DNA replication progulons tend to have short half‐lives, whereas protein‐processing progulons tend to be more stable. In addition, we find that the coordination of mRNA levels correlates with the coordination of mRNA half‐lives, suggesting that mRNA stability is relevant for coordinating mRNA abundance changes of progulons (Fig EV3C). No equivalent, significant relationship exists at the protein level (Fig EV3D).

### Evolutionary conservation

We calculated co‐regulation of proteins across mouse tissues (Geiger *et al*, 2013) to assess if progulon co‐regulation is evolutionary conserved. For this, we analysed all protein pairs that are coexpressed across ProteomeHD (rho > 0.5). Coexpression of proteins of the same human progulon is about twice as likely to be conserved than the coexpression of proteins that have not been assigned to any progulon, or that have been assigned to different progulons (Fig 2I).

### A targeted progulon search identifies new DNA replication proteins

**Figure 3:**
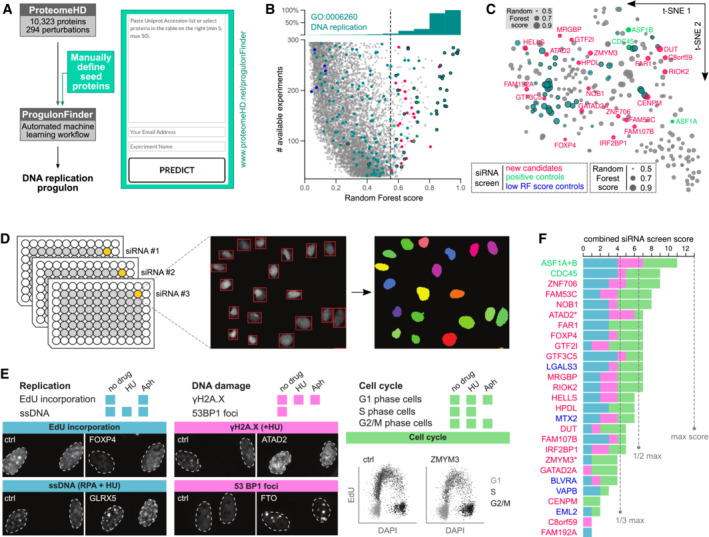
Prediction of new DNA replication factors through the replisome progulon The online version of progulonFinder allows users to specify seed proteins by pasting a list of proteins of interest.We used 41 proteins belonging to the replisome (Alabert *et al*, 2014) to predict a DNA replication progulon, dashed line indicates score‐cutoff. It is heavily enriched in known DNA replication proteins, but also contains many proteins not previously associated with this process. All proteins shown in grey, proteins annotated as DNA replication in GO in turquoise, siRNA screen candidates colour‐coded as shown in legend.t‐SNE plot of the DNA replication progulon highlighting the training proteins (circled) and the candidates selected for siRNA screening.Three siRNAs were used per candidate in a high content imaging setup.Overview of the readouts tested in the siRNA screening, including example images of controls and phenotypes. Assays are designed to capture replication phenotypes either directly or through their downstream impact on DNA damage and cell cycle profiles. EdU incorporation detects DNA synthesis, antibodies against replication protein A (RPA) detect exposed single‐stranded DNA (ssDNA), antibodies against histone H2A.X phosphorylated at S139 and against p53BP1 detect DNA damage. Cell cycle distributions were assessed based on high content imaging of EdU incorporation across cell populations. Some assays included the drugs hydroxyurea (HU) or aphidicolin (Aph) to cause replication stress and trigger phenotypes that might not be visible in unchallenged knockdowns.Cumulative siRNA screen score, ranging from 0 to 13. Assays coloured as in (E), candidates coloured as in (B, C). We consider candidates validated with high confidence if they score in more than half of the assays, and validated with medium confidence if they score in more than a third.

A key advantage that a supervised classification approach has over traditional gene expression analysis is the possibility to target specific proteins of interest, simply by choosing them as training proteins. This opens up a new opportunity for protein function prediction, that is to directly search for proteins that function in a specific biological process. For example, here we identify new factors involved in DNA replication. For this, we created an open and freely available webtool (https://www.proteomehd.net/progulonFinder), which executes our progulonFinder workflow using a list of user‐specified proteins as training data (Fig 3A). In our case, these were 41 well‐characterised core components of the replisome (Alabert & Groth, 2012; Alabert *et al*, 2014; Appendix Fig S7). progulonFinder automatically matched these to the correct protein IDs in ProteomeHD, then created and evaluated a series of Random Forest models (for details, see Materials and Methods and Appendix Fig S2). progulonFinder identified 212 proteins that behave similar to known replisome components (Dataset EV6) in being similarly up‐ or downregulated in response to the 294 perturbations in ProteomeHD. These proteins are heavily enriched in the GO (Ashburner *et al*, 2000) term “DNA replication” (Fig 3B). In addition, many proteins have well‐documented roles in the wider process of replication. This includes DNMT1 and UHRF1, which ensure the faithful propagation of DNA methylation marks, and numerous DNA repair proteins required to fix mismatches and other replication errors.

Dozens of proteins in the replisome progulon have not previously been linked to DNA replication. This progulon therefore has the potential to substantially expand the known repertoire of factors with replication associated functions. In principle, any one of the new candidates could be selected for a mechanistic follow‐up study to identify the precise replication step or subprocess to which it may contribute. However, to estimate the success rate of such endeavours and to validate our approach in general, we subjected 20 candidates to comprehensive profiling of DNA replication phenotypes (Fig 3C). For this, we conducted multiple RNAi screens scoring 20 different phenotypes linked to DNA replication, capturing both direct effects on replication (replication speed, DNA unwinding) and downstream consequences of erroneous replication, such as DNA damage accumulation and cell cycle profile changes (Fig 3D and E). Optionally, we challenged cells with agents causing acute high or permanent low replication stress to uncover related functions (Fig 3E). For two of our candidates, ATAD2 (Koo *et al*, 2016; Lazarchuk *et al*, 2020) and ZMYM3 (Leung *et al*, 2017; Shapson‐Coe *et al*, 2019; Lee *et al*, 2022), existing evidence suggested possible replication‐related functions. In addition, the screen also included three positive controls (known replication function) and five proteins that were predicted not to be involved in DNA replication (low Random Forest (RF) score). We used three siRNAs per candidate in an immunofluorescence staining and high content imaging set‐up (Fig 3D). Individual assay scores (Appendix Figs S8 and S9) were combined into a single “validation score,” based on which we grouped targets into high confidence (> ½ maximum score), medium confidence (> ⅓ maximum score) and unvalidated hits (≤ ⅓ maximum score).

The RNAi screen confirmed a replication‐related function with high confidence for the positive controls and 10 (50%) new candidates (Fig 3F; Dataset EV7). We note that MRGBP was recently shown to negatively regulate homologous recombination repair (Rivero *et al*, 2021) that is required to resolve replication problems (Carvalho *et al*, 2014). This group also included one of the low RF score controls, LGALS3. These controls were selected for a lack of co‐regulation with the replisome, but LGALS3 had previously been functionally linked to homologous recombination (Carvalho *et al*, 2014). This highlights the fact that co‐regulation is a good indicator for shared function, but proteins can also share functions without being co‐regulated. LGALS3, for example, is a multifunctional protein that is not only active in the nucleus but also in the cytoplasm and as a secreted protein. It is likely that replication‐unrelated functions dominate its overall regulatory pattern, explaining the low RF score. Five (25%) additional candidates were validated with medium confidence, including HELLS, which was recently implicated in replication fork protection (Xu *et al*, 2021). The phenotypic evidence did not sufficiently support the remaining five (25%). The latter includes ZMYM3, indicating that our validation rate may be a conservative estimate and could be increased by different validation approaches.

### Selective perturbation is not necessary for protein function prediction

SILAC proteomics enables the accurate quantitation of very small fold‐changes (Ong *et al*, 2002; Kustatscher *et al*, 2019), which means that the indirect, downstream impact of perturbations is often detectable for a large portion of the proteome. This raises the question whether it is necessary to selectively perturb a biological process to identify a related progulon. The replisome progulon offers an opportunity to address this, because 15 of the 294 perturbations in ProteomeHD are from nascent chromatin capture (NCC) experiments (Alabert *et al*, 2014; Nakamura *et al*, 2021). These NCC samples compare newly replicated chromatin with mature chromatin and were treated with drugs that perturb DNA replication, such as hydroxyurea and camptothecin. To test how important these data were for the identification of the replisome progulon, we repeated our prediction without them. The resulting RF Scores are nearly identical (*R* ^2^ 0.97, Appendix Fig S10A and Dataset EV6). However, removing a random set of 15 experiments had a lower impact on the progulon prediction (*R* ^2^ 0.99, Appendix Fig S10B). Therefore, the replication‐related input experiments contributed more to the replisome prediction than others but were not essential for it.

### Perturbation diversity is essential

**Figure EV4:**
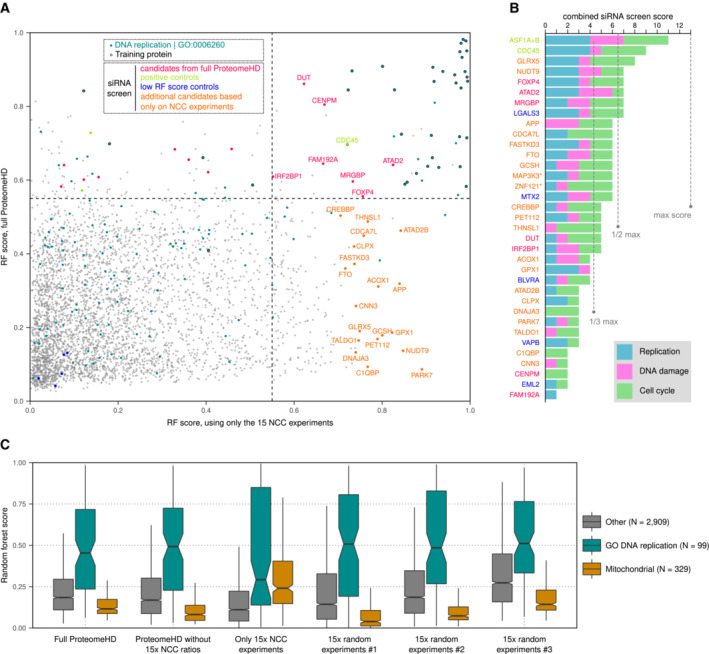
Replisome progulon predictions using only NCC data Replisome progulon prediction using only the 15 Nascent Chromatin Capture (NCC) (Alabert *et al*, 2014) experiments in ProteomeHD, compared to the full set of 294 perturbations. Only proteins that are part of both predictions are shown (some proteins were not detected in NCC experiments, whereas others were only detected in NCC data and therefore did not make the cut‐off to be included in the ProteomeHD‐wide search). Twenty‐one candidates predicted exclusively by focussing on NCC experiments (orange; dashed lines show score cut‐off 0.55) as well as seven candidates predicted by NCC and ProteomeHD (red; upper right corner) were subjected to the high content siRNA screens, which were performed together with the candidates from full ProteomeHD‐based predictions and the appropriate assay controls (see also Fig 3).Results of the NCC‐based screen shown as in Fig 3F. Asterisks mark two genes that are not plotted in (A) because they were only included in the NCC‐only search. Positive controls (green), low‐RF‐score controls (blue) and seven candidates that were predicted by both approaches (red) are also shown in Fig 3F.Boxplot showing RF scores of proteins related to DNA replication, mitochondrial proteins and the remaining proteins. NCC data were either included, left out or used exclusively for the replisome progulon prediction. In addition, 15 random experiments were used exclusively for replisome progulon prediction, and that was repeated three times using different randomly selected experiments. Lower and upper hinges correspond to the first and third quartiles, and lower and upper whiskers extend to the smallest or largest value no further than 1.5 interquartile ranges (IQR) from the hinge. The notches towards the medians (central band) extend 1.58 * IQR/square root (*n*). This gives a roughly 95% confidence interval for comparing medians.

Studying replication intermediates in cells through NCC and iPOND has been invaluable for identifying novel replication fork components (Sirbu *et al*, 2013; Alabert *et al*, 2014, 2015; Dungrawala *et al*, 2015; Cortez, 2017; Nakamura *et al*, 2021). We therefore asked whether we could identify additional replication factors by basing the machine‐learning specifically on the 15 NCC experiments. Using only these data as progulonFinder input yielded very different results (Fig EV4A). Only a fifth of the progulon proteins from the full‐scale analysis were still identified, possibly reflecting that the remaining factors do not function directly at replication forks. Many previously low‐scoring proteins were now classified as co‐regulated. In principle, these could be multifunctional proteins whose replication‐specific activity is obscured by their main function in the global analysis. To test this possibility, we subjected 21 of them to our extensive RNAi screens. These included FTO, C1QBP and CREBBP, for which some prior evidence for a replication‐related or DNA damage‐related function was available (Xiang *et al*, 2017; Dutto *et al*, 2018; Bai *et al*, 2019). Two candidates predicted by the NCC‐only dataset showed a high‐confidence replication phenotype, and 10 showed a medium‐confidence phenotype (Fig EV4B; Dataset EV6). Thus, the validation rate was better for candidates predicted using all of ProteomeHD compared with those predicted based on the 15 directly relevant NCC experiments.

One possible explanation for this is that a diverse range of samples and conditions allows the machine‐learning algorithm to distinguish better between replication‐related and unrelated expression patterns. To test this, we compared the replication progulon RF scores of replication factors and mitochondrial proteins in these experiments (Fig EV4C). Indeed, we found that Random Forests based on either full‐scale or random subsets of ProteomeHD assign very low replication progulon RF scores to mitochondrial proteins. By contrast, using only NCC experiments (which are enriched for freshly replicated chromatin) as input data leads to relatively high scores for mitochondrial proteins. This indicates that a diverse set of random perturbations is important to capture the unique regulatory signature of a biological process in machine‐guided predictions.

## Discussion

The human proteome contains physical protein modules ranging from protein complexes to organelles. The proteome also consists of regulatory modules, but so far these appeared to be limited in size. In part, this is because conventional protein covariation analysis is better suited to capture small regulatory modules, for example tightly co‐regulated protein complex subunits. Challenges associated with identifying large co‐regulation modules include the widespread multifunctional nature of human proteins (Christoforou *et al*, 2016), the varying degree of co‐regulation observed for different cellular processes, and the fact that not all perturbations trigger an equally characteristic response for every protein. We overcome these issues through the use of a machine‐learning‐based workflow, identifying large protein co‐regulation modules that effectively correspond to entire biological processes. One practical application of these protein co‐regulation modules could be gene set enrichment analysis (GSEA; Subramanian *et al*, 2005; Dataset EV8).

The mechanisms of progulon expression control appear to be progulon‐specific, with two contrasting categories emerging from the analysis of extreme cases. Some progulons, such as the cytoskeleton progulons, are characterised by strong mRNA‐to‐protein correlation, suggesting they are primarily controlled via transcription. Other progulons show strong mRNA coexpression but weak mRNA‐to‐protein correlation, and these may be regulated translationally or through protein degradation. One possible mechanism by which such progulons may be controlled is nonexponential protein degradation (NED; McShane *et al*, 2016). Many NED proteins are protein complex subunits that are produced in superstochiometric amounts. These proteins become stabilised by incorporation into the complex, while any excess subunits are degraded. Future work will determine whether NED plays a role for progulon regulation.

While we report here on the discovery of progulons, it is difficult if not impossible to state their exact number or protein composition. There are both biological and technical reasons for this. Different cell types, for example, contain a different set of proteins and require a different set of functionalities. It is therefore expected that their protein co‐regulation modules may be somewhat different, too. The exact composition of a progulon will also be somewhat dependent on a range of technical factors, such as the choice of algorithms and parameter settings, input data and seed lists. In this sense, our current view of progulons is reminiscent of early definitions of protein families based on multiple sequence alignments: on the one hand, it is likely that better algorithms and more/better data will in the future contribute to the discovery of more progulons and affect their precise composition. On the other hand, the existence of such modules, and the ability to detect them using an online tool, may provide a new entry point for the functional characterisation of understudied human proteins.

Finally, the progulon approach offers a new functional proteomics route to specifically search for proteins functioning in a particular biological process. We demonstrate that even for a well‐studied process such as DNA replication, new factors can be identified with high confidence. We expect that our progulonFinder webtool will be useful to researchers from all areas of cell biology to identify new factors contributing to cellular processes of their interest.

## Material and Methods

### General data analysis

Data analysis was performed in R (R Core Team, 2018) and KNIME (Berthold *et al*, 2007). The following R packages were used for all analyses: data.table for fast processing, ggplot2 (Wickham, 2016) for figure making and viridis for colour schemes. The KNIME extension for Weka Data Mining Integration (3.7; Frank *et al*, 2016) was used for Random Forest predictions.

### progulonFinder: Set‐up and considerations

The purpose of progulonFinder is to make automated Random Forest (RF; Breiman, 2001) predictions using ProteomeHD (Kustatscher *et al*, 2019) and very small training sets. Its individual steps are outlined and explained in Appendix Fig S2. Our first goal for progulonFinder was to automate the entire RF machine‐learning procedure, in order to make it accessible to biologists without any computational experience. As a webtool (www.proteomehd.net/progulonFinder), it requires a user to specify nothing but a list of proteins of interest. progulonFinder then automatically and randomly selects negative training data, trains and tests the model, performs cross‐validation and outputs the scores and a report. In addition, advanced users can operate it locally as a KNIME workflow, that is through a graphical user interface that does not require programming skills.

Our second goal was to work with very small training sets. We previously used Random Forests on proteomics data to determine which proteins belong to mitotic chromosomes (Ohta *et al*, 2010), interphase chromatin (Kustatscher *et al*, 2014) and mitochondria (Kustatscher *et al*, 2016). These were more traditional applications in the sense that we could use hundreds of proteins for each training class. However, when operating at the scale of cellular processes and biological pathways, usually only very few well‐known proteins are available for training. We have recently shown that it is possible to create successful RF models with just seven protein complex subunits as positive class, while still using hundreds of unrelated proteins as the negative class (Montaño‐Gutierrez *et al*, 2017). This does, however, create a class imbalance problem, which can reduce prediction accuracy for the minority class, that is our proteins of interest. To reduce this, progulonFinder creates many different RF models using balanced training data, for example 10 positive and 10 negative training proteins, and then averages their result. The number of models depends on the number of proteins of interest (Appendix Fig S2).

### Framework for online version of progulonFinder

The web interface to progulonFinder was written using the Python Flask microframework. The training sets along with the user‐specified contact data are sent to the University of Edinburgh High Performance Computing Cluster, where a prediction job is queued and run when sufficient resources are available. The link with compressed result files is sent to the user‐specified email address using the Mailgun service.

### Creating seed groups by combining OPTICS and clusterONE clustering

Seed protein groups were created by clustering ProteomeHD using the OPTICS (Ankerst *et al*, 1999) and ClusterONE (Nepusz *et al*, 2012) clustering algorithms. For both approaches, we used the treeClust (Buttrey & Whitaker, 2015, 2016) dissimilarity measure, which we have previously found to be an ideal distance metric for isotope‐labelled proteomics data (Kustatscher *et al*, 2019). OPTICS was applied via the dbscan R package (Hahsler *et al*, 2019). The OPTICS parameter Xi (cluster calling steepness threshold) was set to 0.0001, which was found to work well in terms of median cluster size, cluster number and the ability to recover proteins of the ATP synthase complex. ClusterONE was applied via the Java application available at https://paccanarolab.org/cluster‐one. While OPTICS was applied to the entire protein–protein association matrix, ClusterONE was applied only to the top 0.5% of protein pairs with the highest treeClust similarity. The minimal cluster size was set to 4 and the density threshold to 0.4. The seed groups were created by overlapping the groups from these two clustering algorithms, keeping only proteins that were assigned to a group by both algorithms. Since the ClusterONE algorithm creates a partially redundant list of clusters, only the clusters with the highest overlap to the corresponding OPTICS clusters were selected. This procedure resulted in the identification of 72 protein seed groups.

### Identifying progulons from seed proteins

The 72 groups of proteins defined by the clustering were then used as positive training sets to run on an offline version of the progulonFinder workflow. Parameters were set to 500 decision trees of unlimited depth, 1,000 randomly selected negative training proteins, requiring training data to have a minimum of 45 features (SILAC ratios in ProteomeHD) and test data a minimum of 30 features. Not all clusters were successful as training proteins. We expect that for any successful model, the cross‐validated positive training proteins must score very high, especially since there are so few. We therefore discarded 30 progulons that yielded an area under the ROC curve smaller than 0.99 (calculated based on leave‐one‐out cross‐validated training data). In addition, we introduced the requirement that at least four of the 10 proteins with the highest RF scores had to be cross‐validated training proteins. This ensures that, for the smallest training seeds consisting of only four proteins, the entire seed had to be in the top‐scoring proteins. This filter removed another 10 candidate progulons.

Finally, we introduced a “connectivity score” to determine the optimal RF score cut‐off used to assign proteins to progulons. The RF score describes the fraction of trees that voted for a protein to belong to the positive class (1—co‐regulated with positive training set) or to the negative class (0—co‐regulated with the negative training set). This only takes into account whether proteins are co‐regulated with the training proteins and not whether progulon proteins are co‐regulated with each other, that is are forming genuine interconnected modules. To address this, we calculate a connectivity P‐value, which is used to select the appropriate RF score cut‐off such that the resulting module contains proteins that are significantly co‐regulated with each other. For this, we compare progulons to a co‐regulation network created with all proteins in the analysis. Using a Fisher's exact test, we calculate a *P*‐value that reflects if a progulon is enriched for protein pairs that are among the top 0.5% of co‐regulated protein pairs in the overall network. This calculation is performed at a series of RF score cut‐offs, going from 0.5 to 1.0 in 0.01 increments. We then chose the minimum RF score cut‐off that creates a significantly interconnected module. For ~ 80% of the progulons, the default RF score cut‐off of 0.5 already creates a module with a connectivity *P*‐value < 0.05. For the remaining progulons, this approach resulted in more stringent cut‐offs ranging from 0.51 to 0.57, and one progulon had to be discarded completely, because no RF score cut‐off resulted in a significantly interconnected module.

In total, this approach yielded 31 progulons (Dataset EV2). They were manually assigned a short, descriptive name based on their main function.

### t‐Distributed Stochastic Neighbour Embedding

To visualise progulons as scatter plots, we used t‐Distributed Stochastic Neighbour Embedding (t‐SNE; Van Der Maaten & Hinton, 2008) through the Rtsne (Krijthe, 2015) package for R. We used default parameters except theta was set to zero to calculate the exact embedding. For the proteins present in a given progulon, a treeClust distance matrix of ProteomeHD was calculated and this was used as t‐SNE input. The resulting t‐SNE coordinates reflect how similar proteins are in ProteomeHD, for example grouping the subunits of protein complexes together and thus simplifying the visual interpretation. Annotations shown in Fig 1 were made manually using UniProt (The UniProt Consortium, 2017) and the available literature.

### Correlation and functional enrichment analysis

To calculate correlations between proteins, mRNAs and progulons, we use Spearman's rank correlation coefficient (rho) through the R base function. Only pairwise complete observations were used for correlation analysis, that is missing values were ignored. Statistical tests such as Fisher's exact tests and Mann–Whitney significance tests were calculated with R base functions. The Median Absolute Deviation, a robust measure of scale, was used to determine the scale of expression changes. The perm R package was used for permutation testing with 10,000 Monte‐Carlo replications.

Enrichment of progulons for Gene Ontology (GO) terms was tested using the topGO (Alexa & Rahnenfuhrer, 2016) R package. The three aspects (Biological process, Molecular function, Cellular component) of GO were downloaded from QuickGO (Binns *et al*, 2009) with taxon set to human and qualifier to null. Rather than the whole proteome, only proteins that were included in this analysis and had GO annotations were used as the gene “universe” or background for the topGO analysis. Enrichment of GO terms among protein co‐regulation groups was tested considering GO graph structure and using Fisher's exact test.

Enrichment of progulons for Reactome pathways (Fabregat *et al*, 2016) was tested using the “lowest level pathways” Uniprot2reactome.txt table downloaded from https://reactome.org. The pathways were filtered for a minimal size of 20. Only proteins that were included in this analysis and had Reactome annotations were used as the gene “universe” (background). Contingency tables were created for each combination of progulons and Reactome pathways (“In Progulon” or “Not in Progulon” to “In Pathway” or “Not in Pathway”). Each combination was tested for significant pathway enrichment within a progulon through Fisher's exact test and the resulting P‐values were Bonferroni‐corrected.

Enrichment of progulons for hu.Map complexes (Drew *et al*, 2017) was tested using the Protein Complex Map from http://humap2.proteincomplexes.org/download. Enrichment P‐values were calculated as described for Reacome pathways.

### Genomic location of progulon genes

Human genome annotation (GRCh38.p10) was downloaded from ENSEMBL (Zerbino *et al*, 2018), and the enrichment of progulons for genes from the same chromosome was assessed using a two‐sided Fisher's exact test. The chromosome icon map with progulon genes highlighted was created using ENSEMBL's Karyotype display tool.

### mRNA and protein stability and turnover kinetics

mRNA half‐lives in HeLa cells (Tani *et al*, 2012) and protein half‐lives in RPE1 cells (McShane *et al*, 2016) were reported previously. Permutation tests were performed using the perm R package and used 10,000 Monte‐Carlo replications.

### Evolutionary conservation of progulon‐based co‐regulation

We compared co‐regulated protein pairs, defined as pairs with rho > 0.5, between ProteomeHD and the mouse tissue dataset, based on ENSEMBL's one‐to‐one ortholog annotation. Co‐regulated pairs were divided into three groups: (a) neither protein has been assigned to any progulon; (b) pairs where the two co‐regulated proteins were assigned to different progulons; and (c) pairs where both proteins were assigned to the same progulon.

### Replisome progulon predictions

For the prediction of the replisome progulon, we used default parameters as described for progulonFinder above. When using only the 15 NCC or 15 random input ratios, we changed the number of required features to 7 for training proteins and to 5 for test proteins. Dataset EV6 contains information about training protein IDs, feature counts and RF scores obtained for all or subsets of input experiments. Gene Ontology annotation for DNA replication (GO:0006260) was obtained from QuickGO (Binns *et al*, 2009), considering the qualifiers “part of” and “involved in.”

### High‐throughput siRNA screening

siRNA screening candidates selected from both ProteomeHD and NCC‐only predictions were collectively run together in the same experiments. U‐2‐OS cells were grown in DMEM (Gibco) containing 1% Pen/Strep and 10% FBS (Hyclone). The RFP‐PCNA reporter cell line was derived essentially as described (Mejlvang *et al*, 2014). Standard U‐2‐OS or reporter U‐2‐OS cells expressing RFP‐PCNA were reversely transfected with a custom siRNA library (silencer select, Ambion) comprising three independent siRNAs per gene. siRNA control sequences are available in Dataset EV7. In brief, 1.2 μl siRNA (500 nM) was added to 15 μl OptiMEM (Invitrogen) to each well of a 96‐well plate (Greiner #655090). In addition, a 15 μl OptiMEM/0.3 μl Lipofectamine RNAiMAX mix was added and incubated for 20 min. Subsequently, 90 μl of cells was added to give a total cell density of 8,000 cells per well. The final concentration of siRNA was 5 nM. Cells were generally fixed after 48 h. In drug‐challenged set‐ups, cells were incubated for 46 h, followed by hydroxyurea (3 mM, Sigma‐Aldrich) treatment for 2 h; or cells were incubated for 24 h, followed by aphidicolin (0.4 μM) treatment for 24 h. EdU was incorporated 15 and 30 min (without drug treatment and in the presence of aphidicolin, respectively) at a concentration of 40 μM. Cells were fixed directly or subjected to pre‐extraction with CSK buffer (10 mM PIPES pH 7, 100 mM NaCl, 300 mM sucrose, 3 mM MgCl2) containing phosphatase inhibitors (1 mM DTT, 10 μg/ml leupeptin, 10 μg/ml pepstatin, 0.1 mM PMSF, 0.2 mM sodium vanadate, 5 mM sodium fluoride, 10 mM beta‐glycerophosphate) and 0.5% Triton for 5 min on ice, 4% paraformaldehyde fixation.

### Immunocytochemistry and microscopy

EdU visualisation was performed using Click‐iT™ EdU Alexa Fluor® Azide 647 (Invitrogen) according to the manufacturer's instructions. For subsequent immunostaining of endogenous proteins, cells were blocked with PBS containing 5% BSA and 0.1% Tween‐20 for 1 h and incubated with primary antibody overnight at 4 degrees. After washing three times with PBS containing 5% BSA and 0.1% Tween‐20, fluorophore‐coupled secondary antibody was applied for 45 min. DAPI (Sigma) was added to this mix at a final concentration of 1 μg/ml and incubated for another 20 min. Cells were washed three times in PBS. The following primary antibodies were used: mouse anti‐RPA/p34 (Thermo Scientific, MA1‐26418, 1:400), rabbit anti‐H2A.X S139 phosphorylation (Cell Signaling Technology, #2577, 1:500) and rabbit anti‐p53BP1 (Novus Biologicals, NB100‐904, 1:1,000). Secondary antibodies were conjugated with fluorescence labels Alexa488, Alexa568 or Alexa647 (Thermo Fisher Scientific, 1:1,000).

Thirty‐six images per well were acquired with a motorised IX83 wide‐field microscope (Olympus) with PlanSApo 20×/0.75 NA dry objective (> 3,000 nuclei per well). Images were then analysed by the ScanR image analysis software (Olympus). Single‐cell data was further processed and combined in the data visualisation software Spotfire (Tibco).

### Data analysis and scoring

We performed a standard of three independent biological replicates per assay, except in +Aph conditions (six replicates). Due to the biased nature of the library, we normalised for plate‐to‐plate variability based on the negative control siRNAs instead of the plate median. We combined the normalised data from replicates and scored candidates based on their activity as positive “hits” if at least two of three siRNA were above a given threshold in respect to negative control wells. This threshold was set individually per readout based on variability of the assay as measured by the standard deviation (SD) of the negative control wells across plates and replicates. Thresholds were defined as 3× SD for SD ≤ 0.05 (low variability); 2× SD for 0.05 < SD ≤ 0.13 (medium variability); 1× SD for SD > 0.13 (high variability).

For comparison, we evaluated the probability of a gene “hit” based on the collective activities of three siRNAs per gene using the statistical method redundant siRNA activity (RSA) analysis (König *et al*, 2007). Again, we normalised for plate‐to‐plate variability based on the negative control siRNAs instead of the plate median. Candidates with a combined *P*‐fisher value < 0.1 were positively scored. In the set‐up with six biological replicates, we only included candidates that were scoring with at least a single siRNA in five replicates. The SD and RSA scoring method yielded similar results (Appendix Fig S9). The SD‐based method was used for all analyses in this manuscript.

Scoring in one readout (up‐ or downregulation) was counted as “+1” for the cumulative siRNA score. Consequently, the replication phenotypes contributed a maximum of 5 points (EdU, EdU + Aph, RPA, RPA + HU and RPA + Aph) and the DNA damage phenotypes a maximum of 4 (53BP1, γH2A.X, γH2A.X + HU and γH2A.X + Aph). For cell cycle readouts, changes in G1, S and/or G2M populations were scored individually. Since differences in one cell cycle phase occur at the expense of other phases, a maximum of “+1” was counted per experimental condition assessed. This should assure a balanced contribution of the process “cell cycle” to the cumulative siRNA score. There were four experimental conditions assessed (EdU‐ and PCNA‐ based readouts without replication stress, PCNA‐based readout with HU, and EdU‐based readout with Aph), leading to a maximum cumulative score of 4 for the process cell cycle. In total, this scoring system leads to a maximum cumulative score of 13.

### GSEA compatible data formatting

The progulon list was formatted to fit the GSEA compatibility requirements as tab‐separated GMX and GMT data tables (https://www.gsea‐msigdb.org/gsea/index.jsp). Their compatibility was successfully tested on GSEA version 4.2.3.

### Analysed datasets

All proteomics and transcriptomics data used for this manuscript were previously published by us and others. This includes proteomics (Lapek *et al*, 2017) and transcriptomics (Klijn *et al*, 2015) data for breast cancer cell lines, which were obtained by Lapek *et al* (2017). Proteomics (Battle *et al*, 2015) and transcriptomics (Pickrell *et al*, 2010) data for lymphoblastoid cell lines were also described and are available in matched and preprocessed form as Dataset EV1 in ref (Kustatscher *et al*, 2017). We previously reported the re‐processing of a number of transcriptomics studies and their matching with proteomics (Geiger *et al*, 2013) data to analyse expression changes across mouse tissues (Grabowski *et al*, 2018). The matched dataset is available as supplementary file S1 in ref (Grabowski *et al*, 2018).

## Author contributions

**Georg Kustatscher:** Conceptualization; data curation; software; formal analysis; validation; investigation; visualization; methodology; writing – original draft; project administration; writing – review and editing. **Martina Hödl:** Conceptualization; data curation; formal analysis; validation; investigation; methodology; writing – original draft; writing – review and editing. **Edward Rullmann:** Data curation; formal analysis; investigation; visualization; methodology; writing – review and editing. **Piotr Grabowski:** Conceptualization; data curation; software; formal analysis; investigation; methodology; writing – review and editing. **Emmanuel Fiagbedzi:** Formal analysis; investigation; visualization; methodology. **Anja Groth:** Conceptualization; resources; supervision; funding acquisition; investigation; methodology; writing – original draft; project administration; writing – review and editing. **Juri Rappsilber:** Conceptualization; resources; supervision; funding acquisition; investigation; methodology; writing – original draft; project administration; writing – review and editing.

## Disclosure and competing interests statement

A.G. is inventor on a patent covering the therapeutic targeting of TONSL for cancer therapy. A.G. is co‐founder and Chief Scientific Officer of Ankrin Therapeutics. The remaining authors declare that they have no conflict of interest.

## Supporting information



Appendix

Expanded View Figures PDF

Dataset EV1

Dataset EV2

Dataset EV3

Dataset EV4

Dataset EV5

Dataset EV6

Dataset EV7

Dataset EV8

PDF+

## Data Availability

R scripts and KNIME workflows required to reproduce the results of this manuscript are available in GitHub, together with their corresponding input files (https://github.com/Rappsilber‐Laboratory/progulons_v2).
