# Supplementary material for: Higher‐order modular regulation of the human proteome
Source: Mol Syst Biol. 2023 Mar 9;19(5):e9503. doi: 10.15252/msb.20209503 (PMC10167480; doi:10.15252/msb.20209503)
Supplement: Supplementary file 1 — Appendix [file MSB-19-e9503-s008.pdf]

APPENDIX - TABLE OF CONTENTS

Page 2 - Appendix Figure S1:
Weighted correlation network analysis (WGCNA) on ProteomeHD dataset

Page 3 - Appendix Figure S2:
Outline of the progulonFinder workflow

Page 4 - Appendix Figure S3:
(A) Progulon Overlap and (B) GO enrichment for the 31 progulons and 31 control groups.

Page 5 - Appendix Figure S4:
mRNA and protein abundance changes across breast cancer cell lines for progulons and Humap2 protein complexes

Page 6 - Appendix Figure S5:
mRNA and protein abundance changes across lymphoblastoid cell lines (LCLs)

Page 7 - Appendix Figure S6:
mRNA and protein abundance changes across mouse tissues

Page 8 - Appendix Figure S7:
Proteins used as training set to predict DNA replication factors

Page 9 - Appendix Figure S8:
Up- and downregulation of all siRNA screening candidates in all assays

Page 10 - Appendix Figure S9:
High degree of validation overlap between two independent statistical scoring methods

Page 11 - Appendix Figure S10:
Removing NCC data from ProteomeHD has a minor effect on the replisome progulon prediction

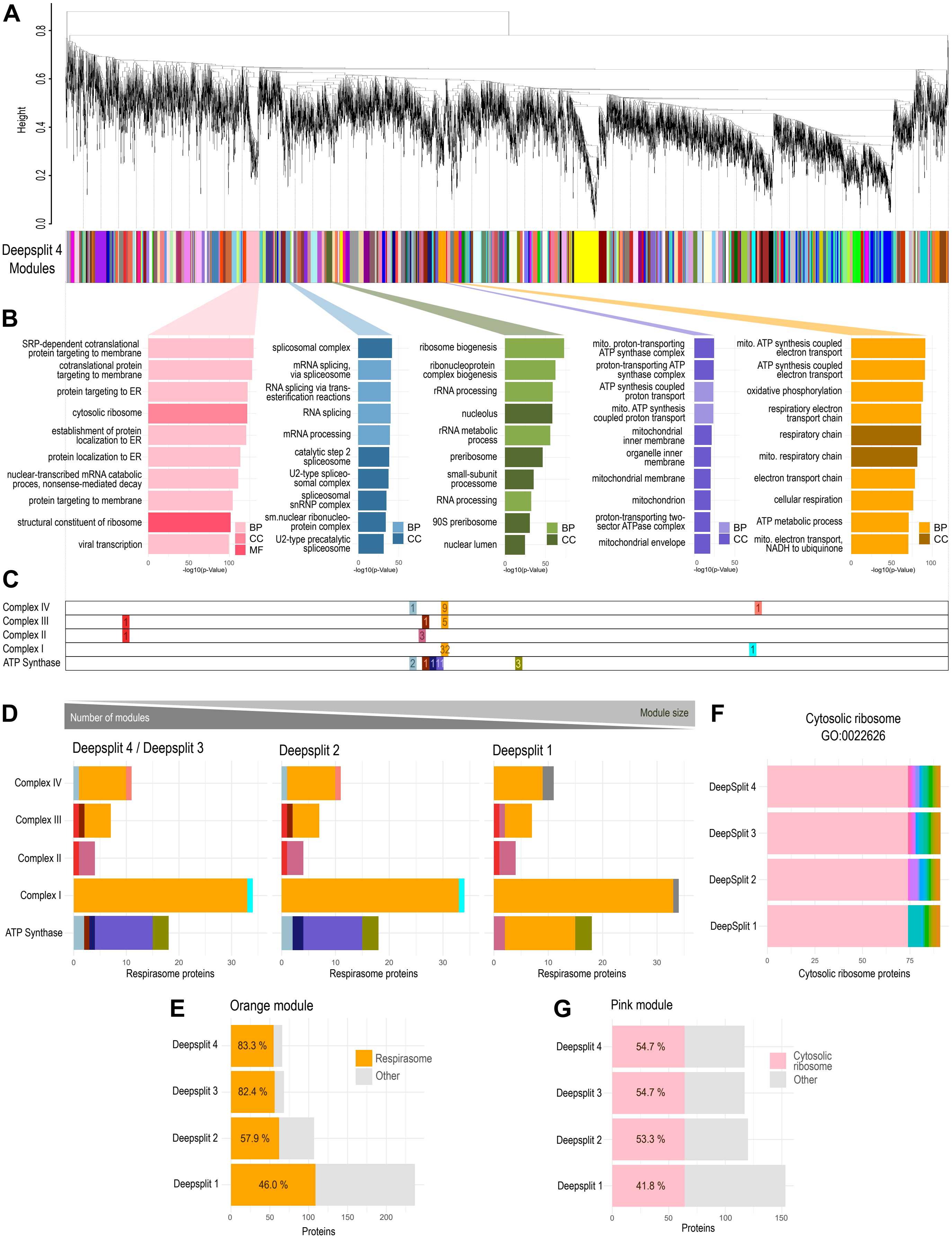

Appendix Figure S1: Weighted correlation network analysis (WGCNA) on ProteomeHD dataset, (**A**) dendrogram and hierarchical clustering of ProteomeHD treeClust distances at WGCNA specific setting Deepsplit = 4. (**B**) WGCNA inbuilt GO enrichment showing the top 10 enriched terms of exemplary modules covering protein localisation (pink), RNA splicing (blue), ribosome biogenesis (green), ATP synthase proton transport (purple) and ATP synthesis electron transport (orange). (**C**) Position of Respirasome (ATP synthase and Complex I-IV) proteins within the dendrogram (Complex I: 32/33 in Orange Module, ATP Synthase: 11/18 in pink Module, other single proteins belong mainly to close proximity modules) (**D**) Distribution of Respirasome proteins (subdivided into Complex I to IV and ATP synthase) across all Deepsplit settings. Higher Deepsplit yields smaller but more modules whereas lower Deepsplit yields bigger but fewer modules. (**E**) coverage of Respirasome proteins within the orange WGCNA module (most enriched for Respirasome) across all Deepsplit settings. Increasing the module size (decreasing Deepsplit parameter) unifies a majority of Respirasome IDs in one module, however also introduces a large number of non associated IDs (**F**) Distribution of cytosolic ribosome proteins (GO:0022626) across all Deepsplit settings and (**G**) the coverage of cytosolic ribosome proteins within the most enriched for cytosolic ribosome WGCNA module (bottom) shows high consistency across parameter variation. In general D and E show that WGCNA needs parameter tuning to find a balance between precision and completeness with constraints on either side.

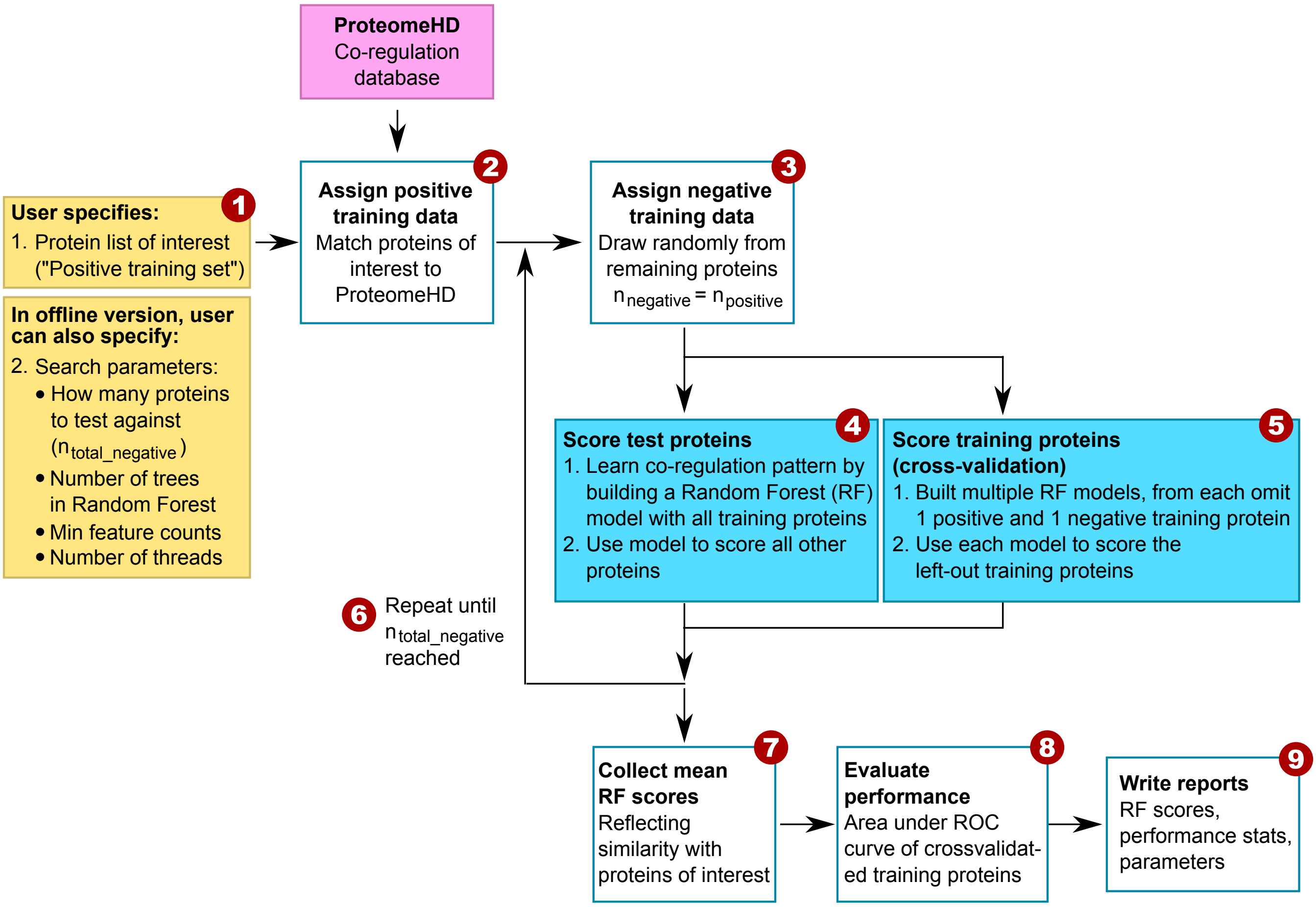

Appendix Figure S2. Outline of the progulonFinder workflow

ProgulonFinder is a workflow to make semi-automated Random Forest predictions based on ProteomeHD. The online version uses only a list of proteins as input, the offline version also allows manual parameter adjustments. Both versions break down into the following steps:

1. User specifies proteins of interest, i.e. a list of of Uniprot IDs. Limited to min. 5, max. 50 in the online version.

2. Find which of these proteins (and which actual isoforms) are detected in ProteomeHD and assign them a “positive class” training label. To increase robustness, no training labels are assigned to any protein quantified in less than 45 experiments (15% of ProteomeHD).

3. Randomly select "negative" training proteins from the remaining entries in ProteomeHD, equal in number to the matched positive training proteins. In this way, the size of the training classes will be balanced (see also step 6).

4. These training proteins are used to train a Random Forest model. Then all remaining (non-training) proteins are run through that model and the probability for each of them to belong to the “positive” (1) or “negative” (0) class is recorded. Only proteins which were observed in at least 30 (10%) experiments in ProteomeHD are scored.

5. Leave-one-out cross-validation of training proteins. This is used for performance evaluation and to provide unbiased scores for the training proteins. Multiple Random Forests are created, each without one of the positive and one of the negative training proteins. These models are then used to score the left-out training proteins.

6. Scores of test proteins and cross-validated scores of training proteins are combined and saved. Then steps 3 - 5 are repeated until a total of ~1,000 different negative training proteins have been used. This repetition is necessary to provide reliable predictions for such small sets of positive training proteins without creating a class imbalance issue. See Methods sections for more details.

   For example, a user may upload a list of 10 proteins, 7 of which are found in ProteomeHD. This is the positive training set. From the remaining proteins in ProteomeHD, 7 are randomly drawn as negative training set. A Random Forest is built with these 14 training proteins and used to score all other proteins. In parallel, 7 additional Random Forests are built, each omitting one positive and one negative training protein, which are then scored by these models. This entire process is repeated 143 times, each time using 7 different negative training proteins (in total, the 7 positive training proteins are therefore compared to 7 x 143 = 1,001 random negative training proteins).

7. Average Random Forest scores and their standard deviation are calculated. The standard deviation gives an indication of how robust the score is towards selecting different negative training proteins at random.

8. To evaluate the performance, cross-validated scores of training proteins are used to generate a ROC curve.

9. Final Random Forest scores are written out, together with a PDF report containing a plot and a methods summary to be used in any publications arising from this tool.

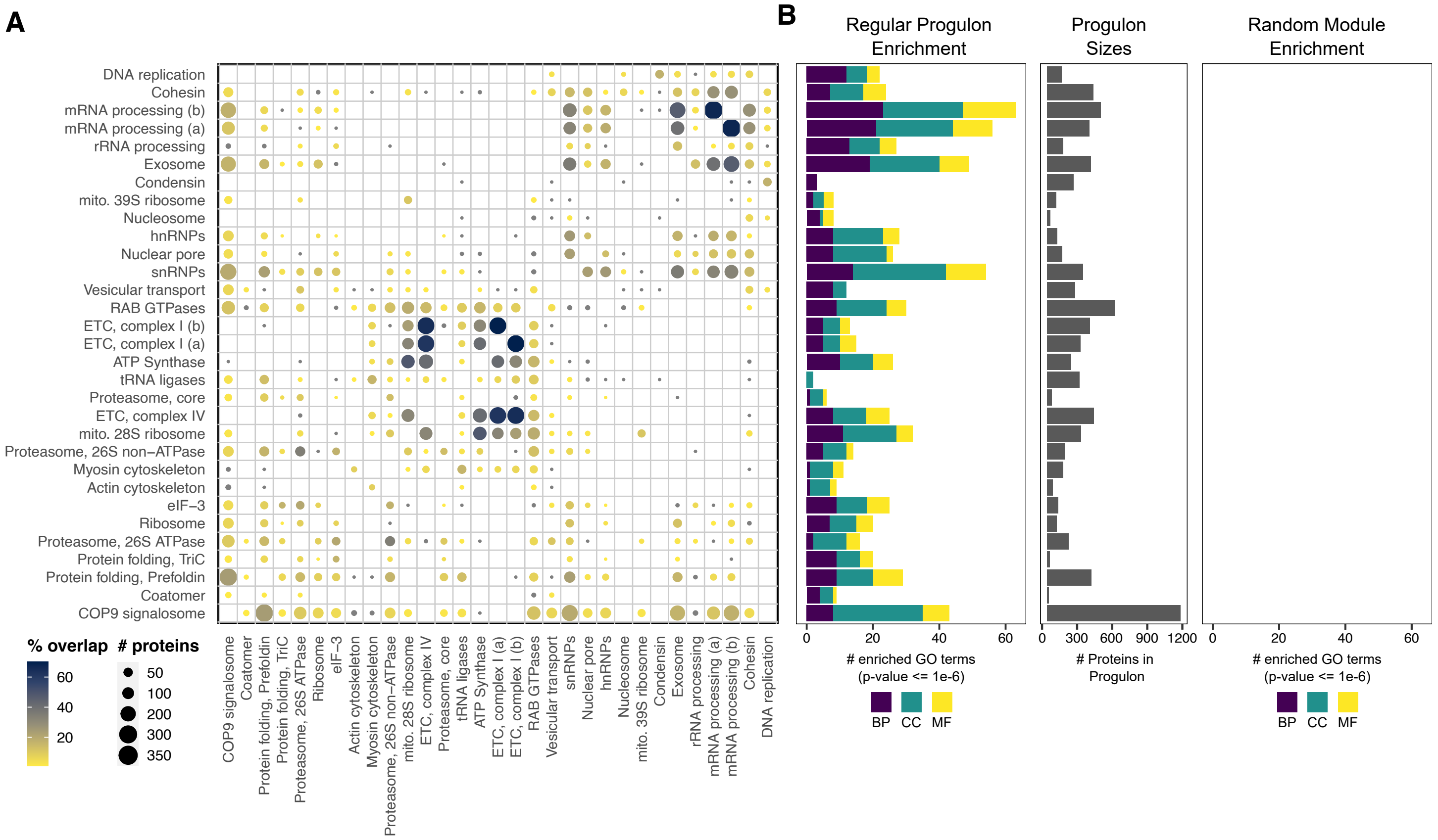

Appendix Figure S3. (A) Progulon Overlap and (B) GO enrichment for the 31 progulons and 31 control groups. The latter consist of randomly generated groups of proteins with the same size distribution as the real progulons. (**A**) Although the 31 seed groups are non-redundant, there is some overlap between progulons that were seeded by functionally similar proteins, such as the two progulons seeded by a different set of mRNA processing factors or different subunits of complex I of the electron transport chain (ETC). The percentage overlap is given relative to the bigger of the two progulons in each pair. (**B**) There was no GO term enrichment for randomly generated modules with identical sizes to progulons when applying the same p-value cut-off. Note the left panel (real progulons) is identical to the left-most panel in Fig 1, it is re-produced here for comparison with the random control groups.

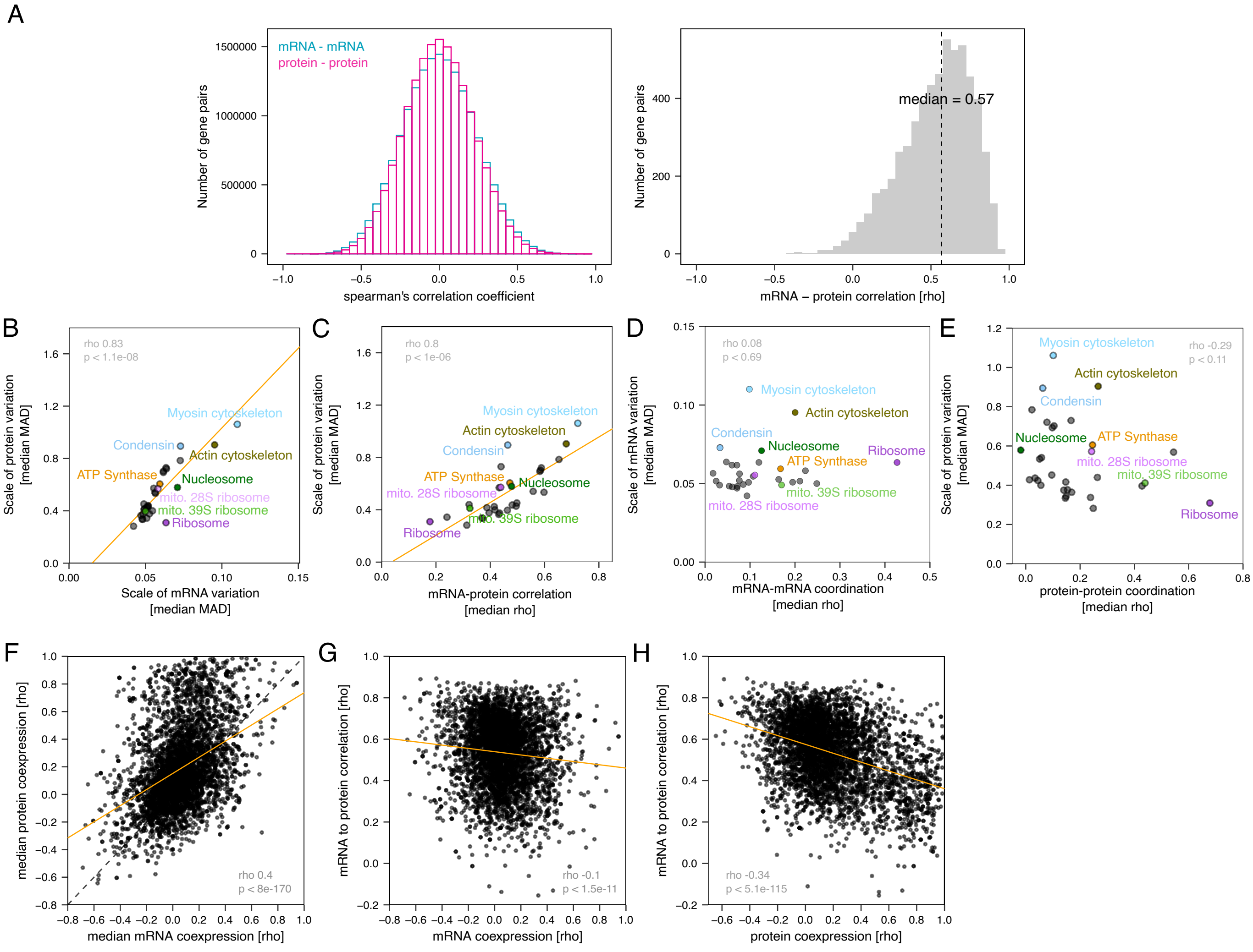

Appendix Figure S4. mRNA and protein abundance changes across breast cancer cell lines for Progulons and Humap2 protein complexes

(**A-E**) For Progulons: (**A**) Distribution of mRNA - mRNA and protein - protein correlation coefficients (left) as well as mRNA - to - protein correlations (right) for the breast cancer cell line data. The median mRNA - to - protein correlation is 0.57. (**B**) Progulons with higher mRNA variation also have higher protein variation. Expression variation between breast cancer samples was determined using a robust measure of scale, the median absolute deviation (MAD). The dots show the median of the MADs of the proteins (or mRNAs) in each progulon. (**C**) The contribution of mRNA to protein abundance changes, estimated by the correlation between the two, correlates strongly with scale of protein variation. This indicates that larger expression changes generally require more transcriptional regulation. (**D**) In contrast, the degree of coordination of mRNA abundance changes is independent of the scale of expression changes. (**E**) Same as (**D**) but for protein levels.
(**F-H**) For Humap2 protein complexes: (**F**) Protein coordination increases with the mRNA coordination (rho, orange regression line). The majority of Humap2 protein complexes are located on the upper side of the diagonal dashed line, suggesting that they are better coordinated on the protein level. (**G, H**) The mRNA-to-protein contribution is inversely correlated (rho, orange regression line) with both mRNA and protein coordination.

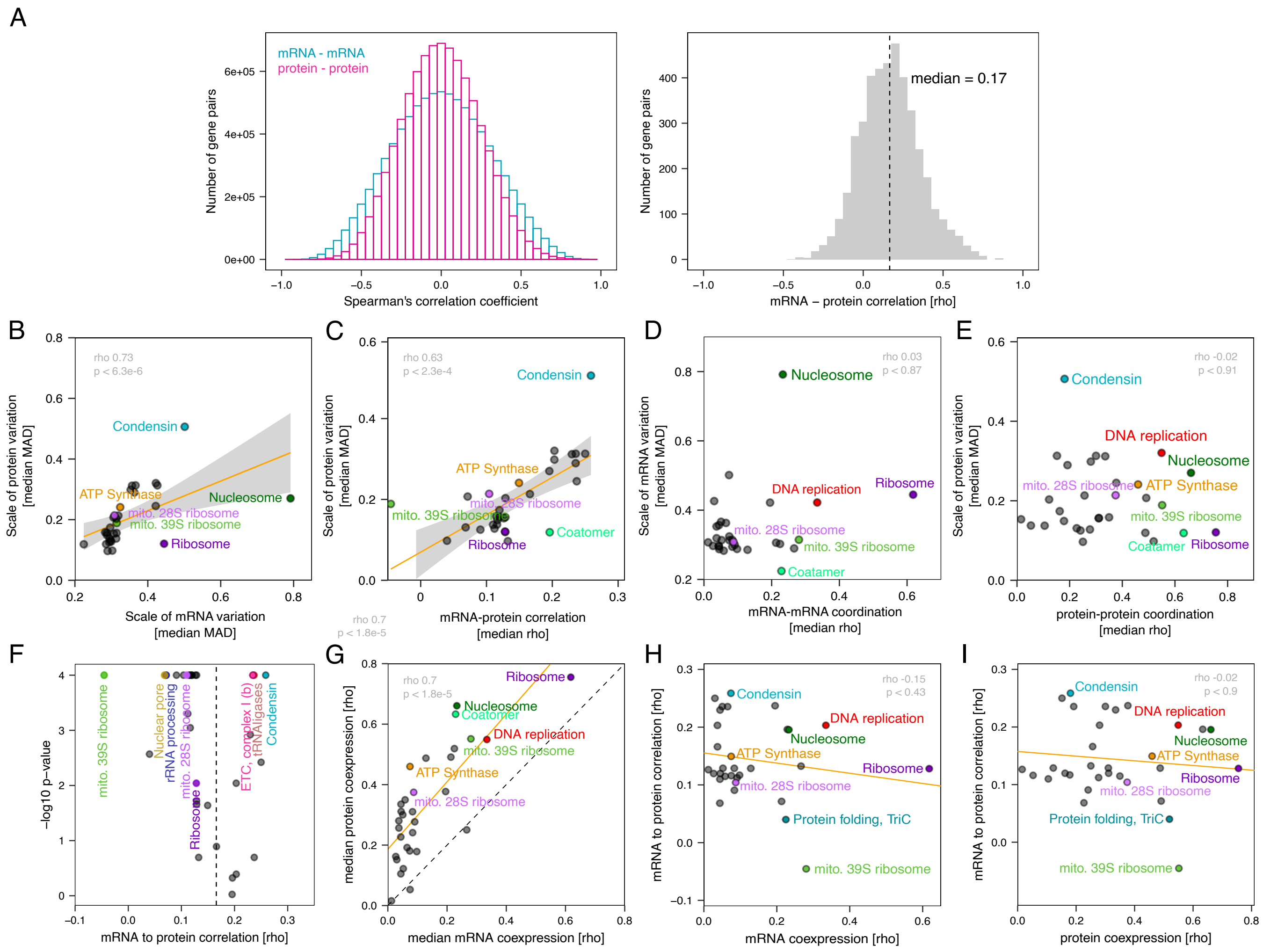

Appendix Figure S5. mRNA and protein abundance changes across lymphoblastoid cell lines (LCLs)

This figure reports the analysis of the LCL dataset, equivalent to the analysis of the breast cancer dataset discussed in the main text and shown in Figure 2 and Appendix Figure S4. (**A**) Distribution of mRNA - mRNA and protein - protein correlation coefficients (left) as well as mRNA - to - protein correlations (right) for the LCL dataset. The median mRNA - to - protein correlation is 0.17. (**B**) Expression variation between lymphoblastoid cell lines was determined using the median absolute deviation (MAD), a measure of scale that is used as a robust alternative to the standard deviation. The dots show the median of the MADs of the proteins (or mRNAs) in each progulon. Progulons with higher mRNA variation also have higher protein variation. (**C**) The contribution of mRNA to protein abundance changes, estimated by the correlation between the two, correlates strongly with the extent of protein variation. (**D**) In contrast, the degree of coordination of mRNA abundance changes is independent of the scale of expression changes. (**E**) Same as (**D**) but for protein levels. (**F**) The median mRNA-to-protein correlation in the dataset is 0.17, but the median rho of genes assigned to different progulons can deviate significantly from that (p-values from permutation testing). (**G**) Protein coordination increases with the mRNA coordination (orange regression line), but most progulons are much better coordinated at the protein level, i.e. they are on the upper side of diagonal, which is indicated by a dashed line. (**H**) The mRNA-to-protein contribution is not correlated with mRNA coordination. (**I**) Same as (**H**) but for protein levels.

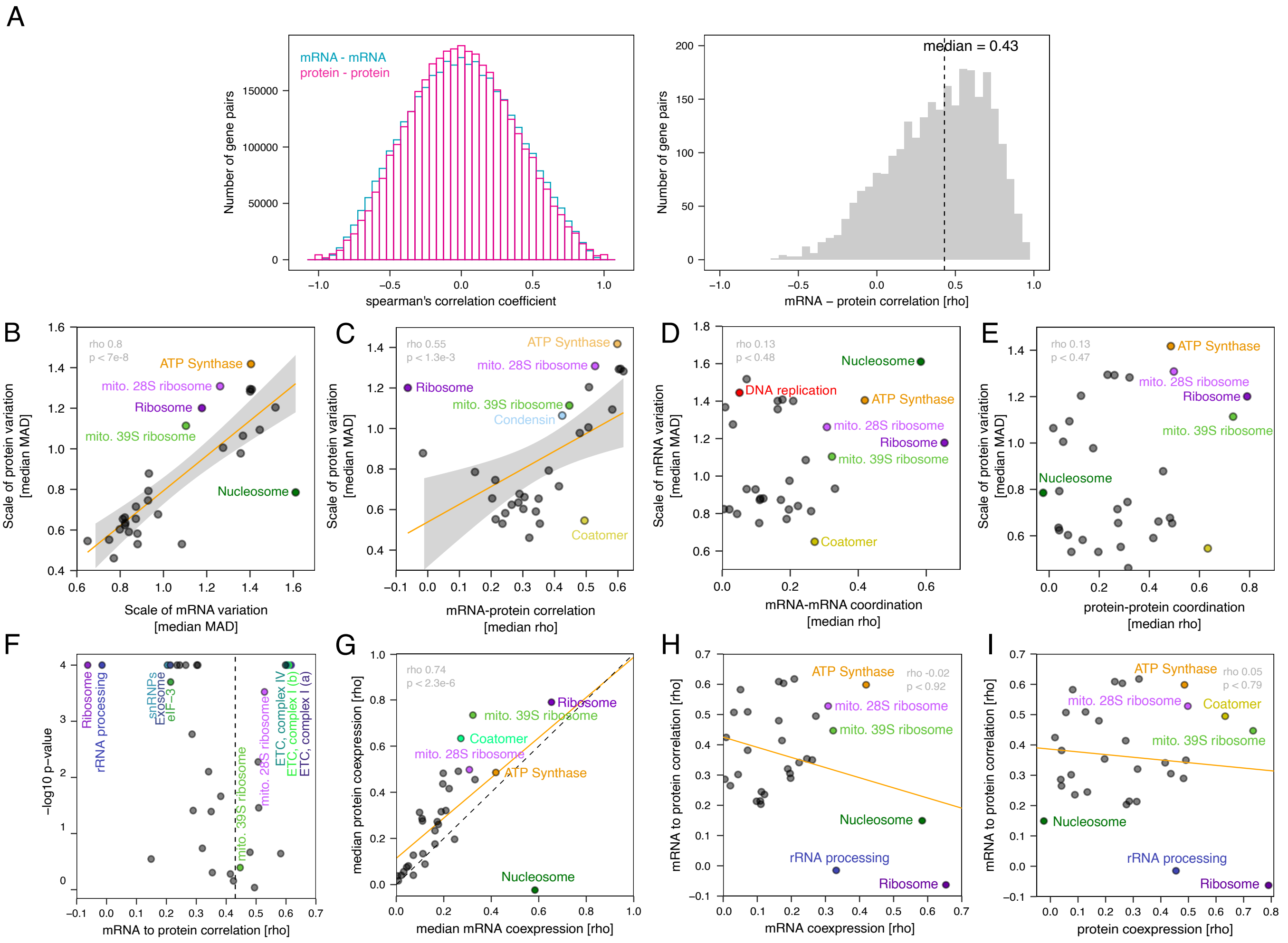

Appendix Figure S6. mRNA and protein abundance changes across mouse tissues

This figure reports the analysis of the mjouse tissue dataset, equivalent to the analysis of the breast cancer dataset discussed in the main text and shown in Figure 2 and Appendix Figure S4. (**A**) Distribution of mRNA - mRNA and protein - protein correlation coefficients (left) as well as mRNA - to - protein correlations (right) for the LCL dataset. The median mRNA - to - protein correlation is 0.43. (**B**) Expression variation between lymphoblastoid cell lines was determined using the median absolute deviation (MAD), a measure of scale that is used as a robust alternative to the standard deviation. The dots show the median of the MADs of the proteins (or mRNAs) in each progulon. Progulons with higher mRNA variation also have higher protein variation. (**C**) The contribution of mRNA to protein abundance changes, estimated by the correlation between the two, correlates with the extent of protein variation. (**D**) In contrast, the degree of coordination of mRNA abundance changes is independent of the scale of expression changes. (**E**) Same as (**D**) but for protein levels. (**F**) The median mRNA-to-protein correlation in the dataset is 0.43, but the median rho of genes assigned to different progulons can deviate significantly from that (p-values from permutation testing). (**G**) Protein coordination increases with the mRNA coordination (orange regression line), but most progulons are better coordinated at the protein level, i.e. they are on the upper side of diagonal, which is indicated by a dashed line. (**H**) The mRNA-to-protein contribution is not correlated with mRNA coordination. (**I**) Same as (**H**) but for protein levels.

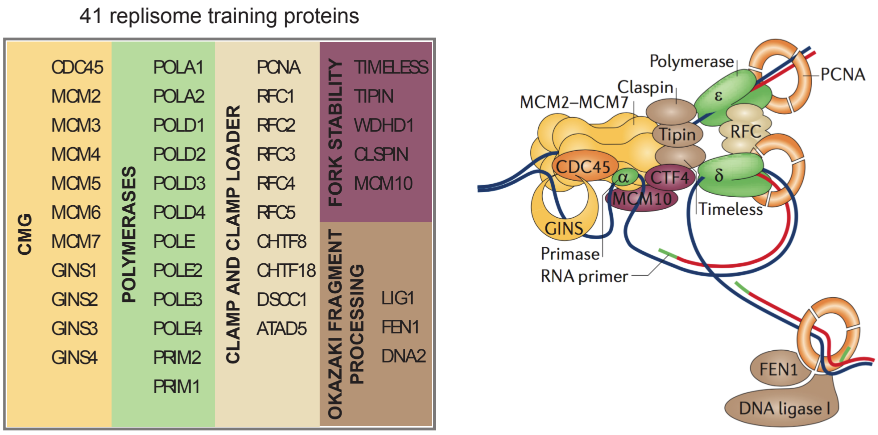

Appendix Figure S7. Proteins used as training set to predict DNA replication factors

These 41 replisome proteins were used to predict the replisome progulon. The basic structure of the replisome is shown on the right (modified from Alabert and Groth, *Nat Rev Mol Cell Biol*, 2012).

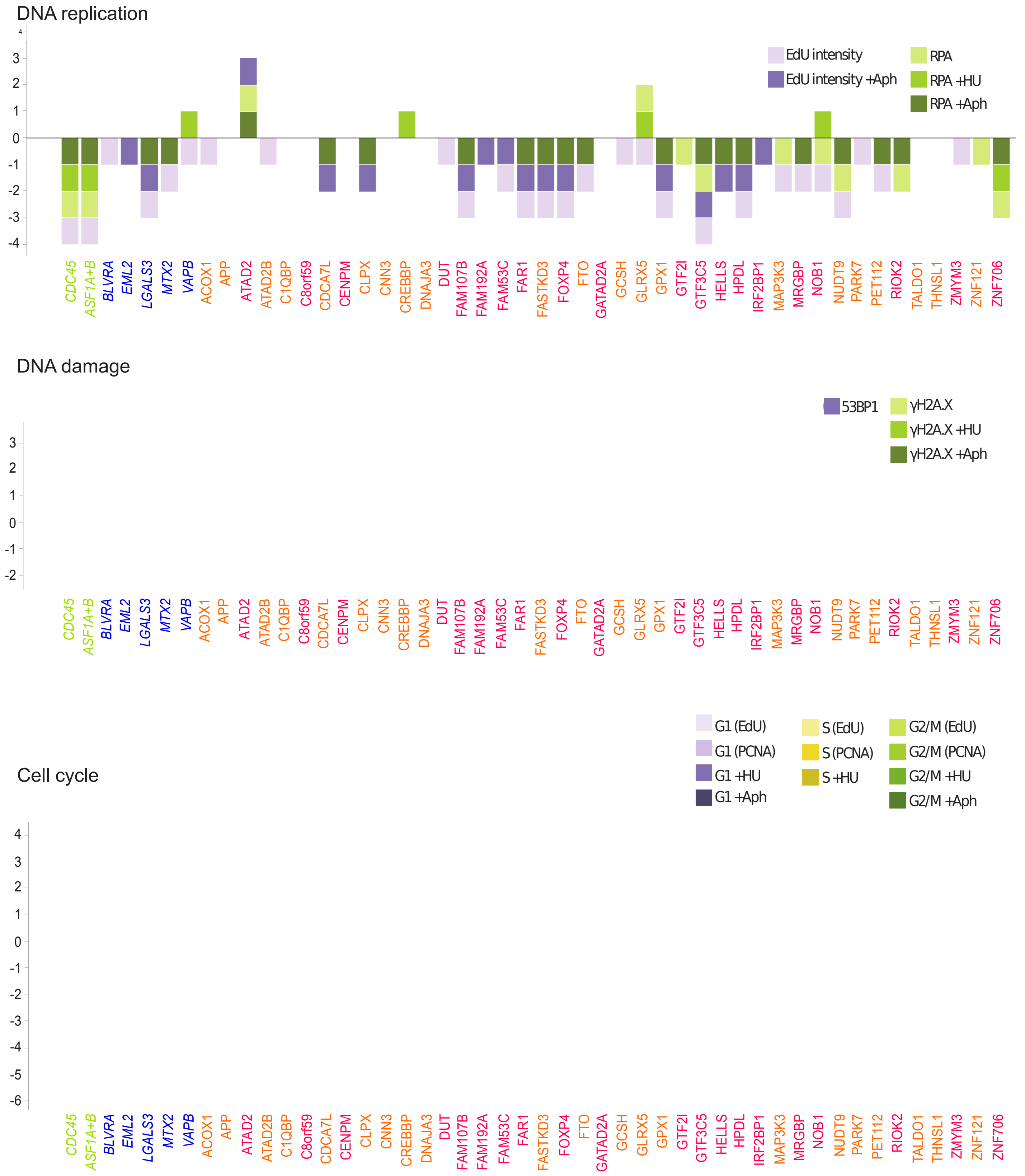

Appendix Figure S8: Up- and downregulation of all siRNA screening candidates in all assays

Positive scores reflect upregulation, negative scores reflect downregulation. EdU incorporation detects DNA synthesis, antibodies against replication protein A (RPA) detect exposed single-stranded DNA (ssDNA), antibodies against histone H2A.X phosphorylated at S139 and against p53BP1 detect DNA damage. Cell cycle distributions were assessed based on high content imaging of EdU incorporation across cell populations. Some assays included the drugs hydroxyurea (HU) or aphidicolin (Aph) to cause replication stress and trigger phenotypes that might not be visible in unchallenged knock-downs.

Scoring in one readout (up- or downregulation) was counted as "+1" for the cumulative siRNA score. Consequently, the replication phenotypes contributed a maximum of 5 points (EdU, EdU + Aph, RPA, RPA + HU, RPA + Aph) and the DNA damage phenotypes a maximum of 4 (53BP1, γH2A.X, γH2A.X + HU, γH2A.X + Aph). For cell cycle readouts, changes in G1, S and/or G2M populations were scored individually. Since differences in one cell cycle phase occur at the expense of other phases, a maximum of "+1" was counted per experimental condition assessed. This should assure a balanced contribution of the process "cell cycle" to the cumulative siRNA score. There were four experimental conditions assessed (EdU- and PCNA- based readouts without replication stress, PCNA-based readout with HU, and EdU-based readout with Aph), leading to a maximum cumulative score of 4 for the process cell cycle. In total, this scoring system leads to a maximum cumulative score of 13.

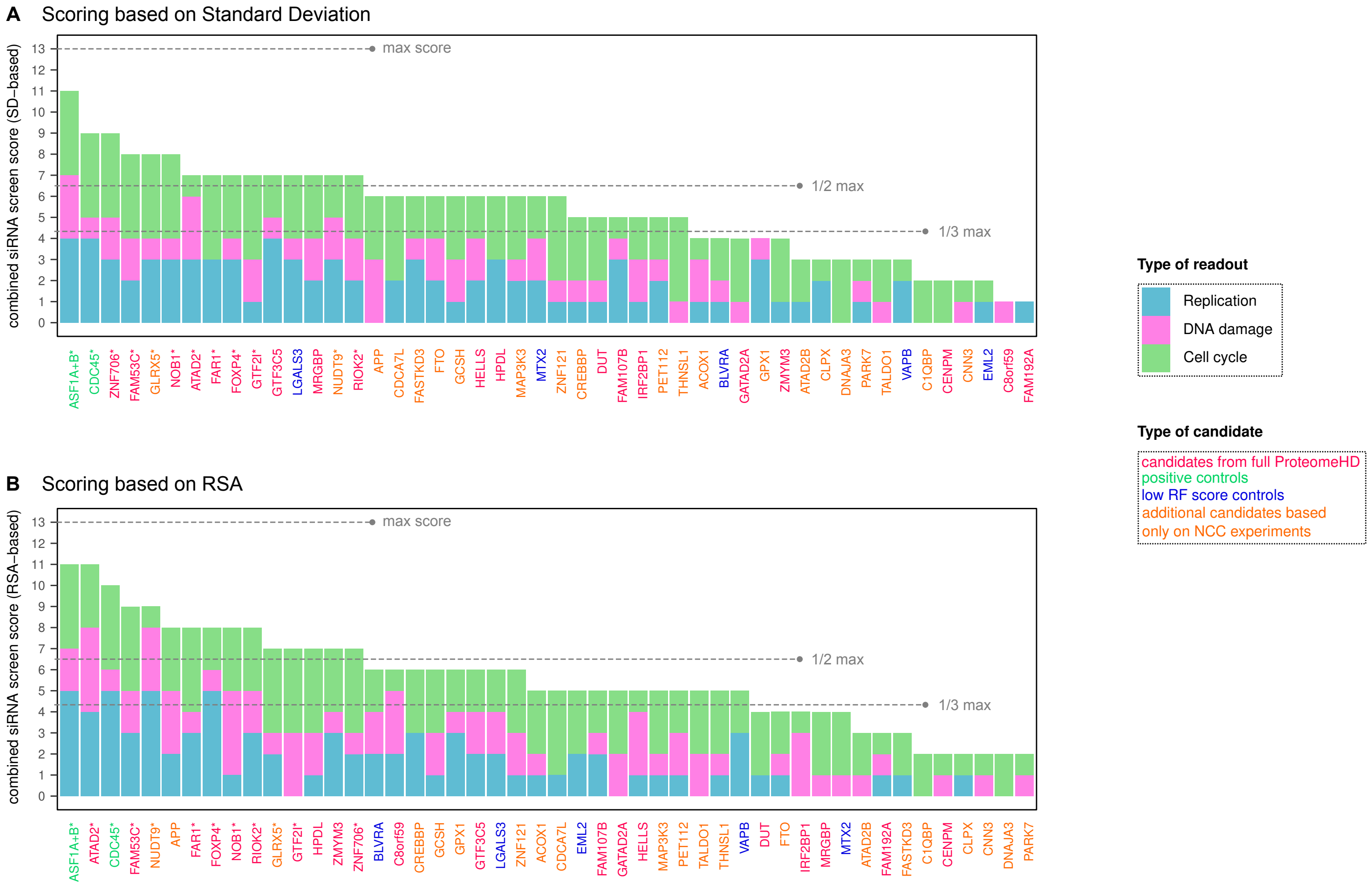

Appendix Figure S9: High degree of validation overlap between two independent statistical scoring methods

Cumulative screening score as determined based on standard deviation (A) and RSA (B). Asterisks incidate proteins that scored above the high-confidence threshold with both methods. The SD method was used for the main figures and analysis. See methods section for details about how these scores were calculated.

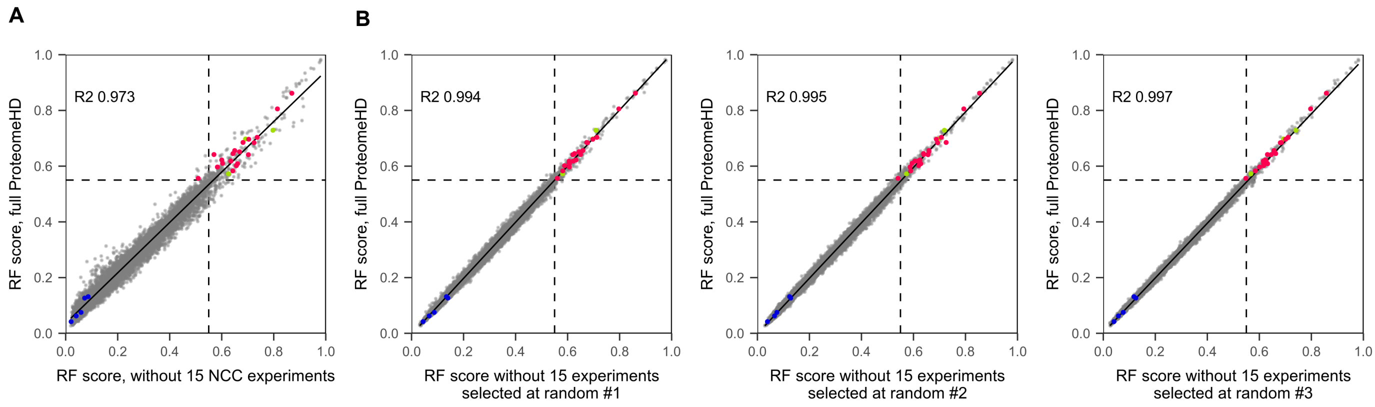

Appendix Figure S10. Removing NCC data from ProteomeHD has a minor effect on the replisome progulon prediction
(A) Omitting the 15 NCC experiments from ProteomeHD has a minor effect on the replisome Random Forest scores. Green, blue and magenta proteins are positive and negative controls and siRNA screen candidates as shown in Fig 3. **(B)** However, removing a set of 15 random experiments from ProteomeHD has even less of an effect. This experiment was repeated three times and each time the impact was less than when removing the 15 NCC ratios.
