# Supplementary material for: Higher‐order modular regulation of the human proteome
Source: Mol Syst Biol. 2023 Mar 9;19(5):e9503. doi: 10.15252/msb.20209503 (PMC10167480; doi:10.15252/msb.20209503)
Supplement: Supplementary file 2 — Expanded View Figures PDF [file MSB-19-e9503-s006.pdf]

Expanded View Figures

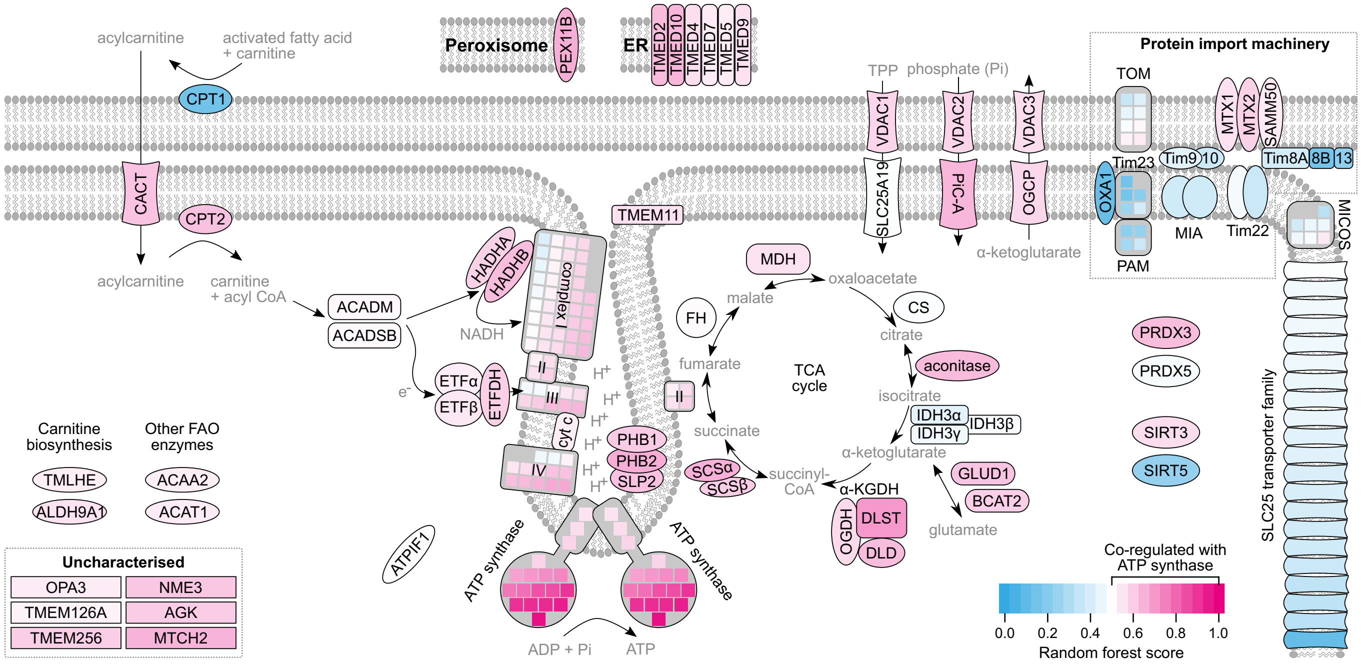

Figure EV1. Outline of the ATP synthase progulon.

Drawing of ATP synthase related biological processes, colour-coded according to how strongly each protein is co-regulated with the ATP synthase. This includes almost every protein of the electron transport chain (complexes I-IV) and the fatty acid β-oxidation (FAO) pathway except its rate-limiting enzyme CPT1. This suggests that up- or downregulation of the ATP synthase is generally accompanied by a corresponding change in the pathways building up the proton gradient. About 60% of proteins in this progulon have a known function that is clearly linked to ATP synthesis and these are shown here. See Dataset EV3 for a full protein list. The protein that most closely matches the ATP synthase expression pattern, DLST, is part of the TCA cycle in the mitochondrial matrix. As a subunit of the α-ketoglutarate dehydrogenase, DLST depletes the endogenous ATP synthase inhibitor α-ketoglutarate (α-KG) (Chin *et al*, 2014). Three other top hits either metabolise (GLUD1, BCAT2) or export α-KG (OGCP). By contrast, isocitrate dehydrogenase, which generates α-KG, is a notable absence among the TCA cycle enzymes, suggesting that part of the biological significance of this progulon may be to prevent metabolic inhibition of ATP synthesis. A third function of the progulon may be to reduce the impact of reactive oxygen species (ROS), which are by-products of ATP synthesis. For example, among the strongest co-regulation partners of the ATP synthase are the two most ROS-sensitive enzymes of the TCA cycle, DLST and aconitase, both of which can be readily inactivated by oxidative damage. Coordinating their expression with the respirasome may be a way to ensure flux through the cycle even in the presence of oxidative stress. Other high-scoring proteins include the antioxidant peroxiredoxin III and PEX11B, which creates peroxisome-mitochondria connections thought to alleviate oxidative stress on mitochondria (Kustatscher *et al*, 2019). Control proteins that localise to the inner membrane but are not directly related to ATP synthesis are absent from the progulon. This includes the MICOS complex, the protein import machinery and the bulk of the SLC25 transporter family (some SLC25 proteins have ATP synthesis-related functions, for example PiC-A imports the substrate inorganic phosphate).

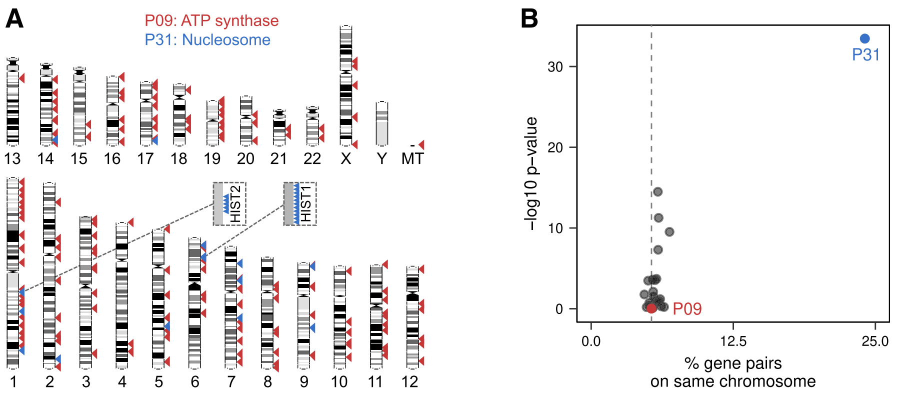

Figure EV2. Progulons are not linked to gene position.

- A Human chromosomes showing the location of the genes involved in the ATP synthase (red) and Nucleosome (blue) progulons. HIST1 and HIST2 are two histone gene clusters on chromosomes 1 and 6, respectively.
- B Except for this nucleosome progulon, progulons are not strongly enriched for genes from the same chromosome. A dashed line indicates the 5.3% of gene pairs that would be expected to be on the same chromosome by random chance; *P*-values are from a two-sided Fisher's exact test.

© 2023 The Authors

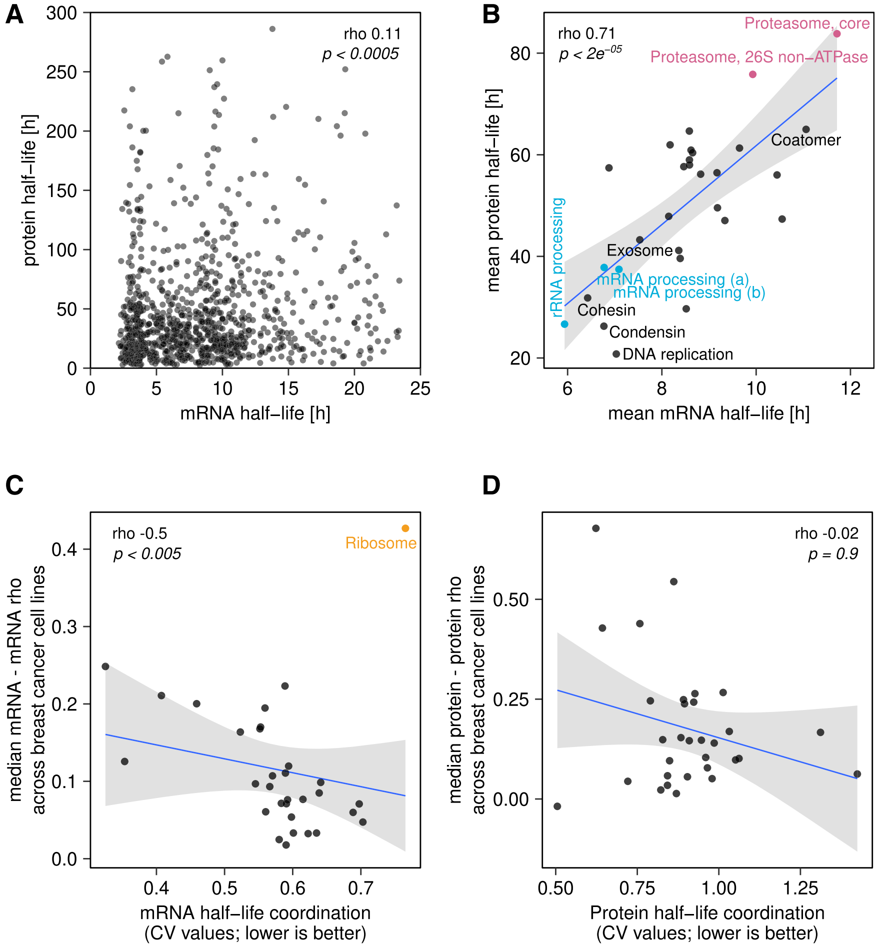

Figure EV3. mRNA and protein half-lives of progulons.

A The mRNA and protein half-lives of individual genes are correlated only very weakly.

B The average half-life of all proteins and mRNAs of a progulon show a strong and significant correlation. This is even though mRNA half-lives were measured in HeLa cells (Tani *et al*, 2012) and protein half-lives in RPE1 cells (McShane *et al*, 2016). Note that proteins are longer lived than mRNAs.

C The coordination of mRNA half-lives within progulons correlates with the degree of coordination of mRNA expression changes across the breast cancer cell line panel.

D No equivalent significant relationship is observed on the protein level.

© 2023 The Authors

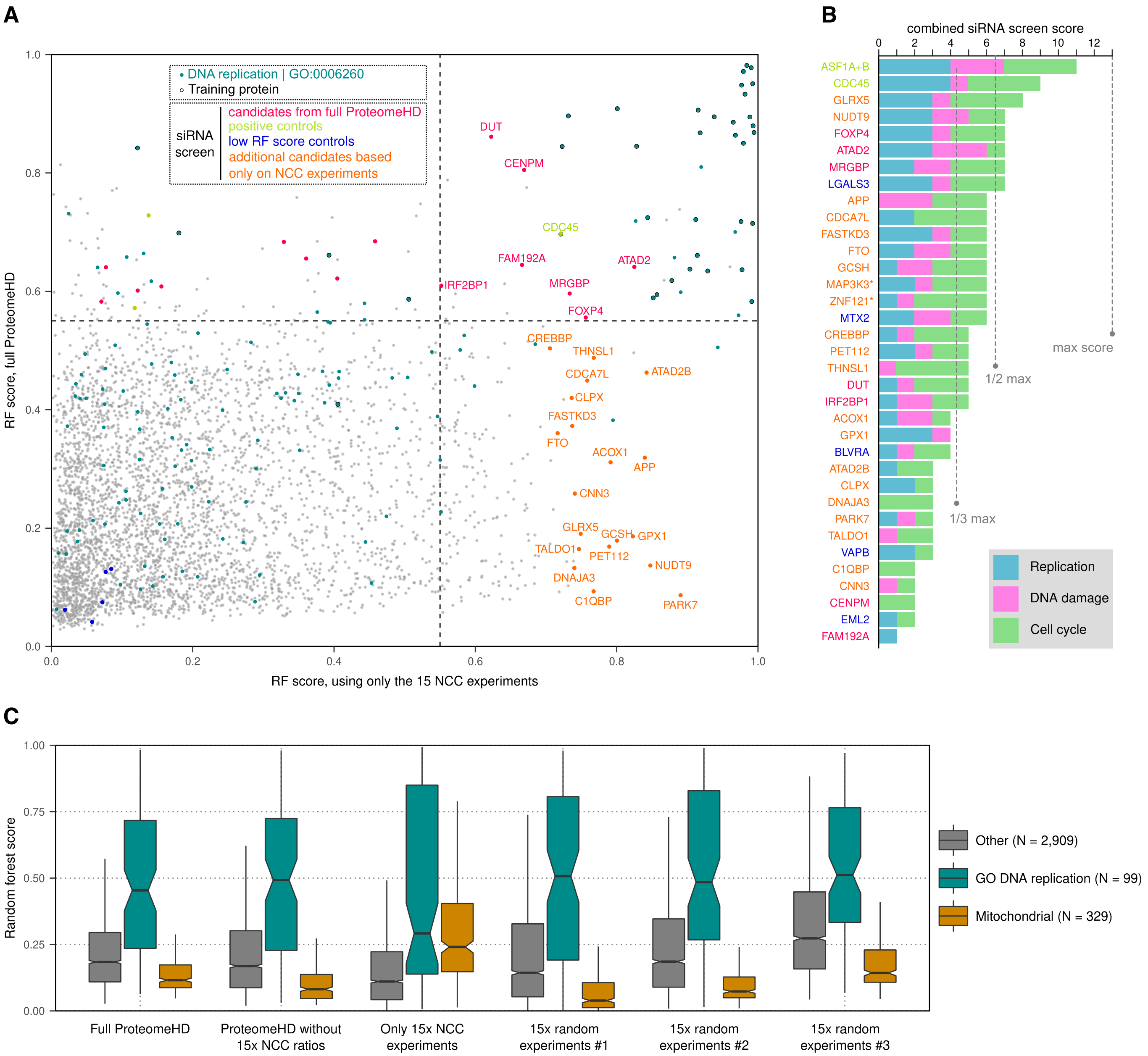

Figure EV4. Replisome progulon predictions using only NCC data.

A Replisome progulon prediction using only the 15 Nascent Chromatin Capture (NCC) (Alabert *et al*, 2014) experiments in ProteomeHD, compared to the full set of 294 perturbations. Only proteins that are part of both predictions are shown (some proteins were not detected in NCC experiments, whereas others were only detected in NCC data and therefore did not make the cut-off to be included in the ProteomeHD-wide search). Twenty-one candidates predicted exclusively by focussing on NCC experiments (orange; dashed lines show score cut-off 0.55) as well as seven candidates predicted by NCC and ProteomeHD (red; upper right corner) were subjected to the high content siRNA screens, which were performed together with the candidates from full ProteomeHD-based predictions and the appropriate assay controls (see also Fig 3).

B Results of the NCC-based screen shown as in Fig 3F. Asterisks mark two genes that are not plotted in (A) because they were only included in the NCC-only search. Positive controls (green), low-RF-score controls (blue) and seven candidates that were predicted by both approaches (red) are also shown in Fig 3F.

C Boxplot showing RF scores of proteins related to DNA replication, mitochondrial proteins and the remaining proteins. NCC data were either included, left out or used exclusively for the replisome progulon prediction. In addition, 15 random experiments were used exclusively for replisome progulon prediction, and that was repeated three times using different randomly selected experiments. Lower and upper hinges correspond to the first and third quartiles, and lower and upper whiskers extend to the smallest or largest value no further than 1.5 interquartile ranges (IQR) from the hinge. The notches towards the medians (central band) extend 1.58 * IQR/square root (*n*). This gives a roughly 95% confidence interval for comparing medians.

© 2023 The Authors
