# Supplementary material for: Higher‐order modular regulation of the human proteome
Source: Mol Syst Biol. 2023 Mar 9;19(5):e9503. doi: 10.15252/msb.20209503 (PMC10167480; doi:10.15252/msb.20209503)
Supplement: Supplementary file 12 [file MSB-19-e9503-s012.pdf]

molecular systems biology

Higher-order modular regulation of the human proteome

Georg Kustatscher, Martina Hödl, Edward Rullmann, Piotr Grabowski, Emmanuel Fiagbedzi, Anja Groth, and Juri Rappsilber
DOI: 10.15252/msb.20-99503

Corresponding author(s): Juri Rappsilber (Juri.Rappsilber@TU-Berlin.de) , Anja Groth (anja.groth@cpr.ku.dk), Georg Kustatscher (georg.kustatscher@ed.ac.uk)

Review Timeline:

Submission Date:	6th Feb 20
Editorial Decision:	3rd Mar 20
Revision Received:	17th Aug 22
Editorial Decision:	20th Sep 22
Revision Received:	8th Feb 23
Accepted:	14th Feb 23

Editor: Maria Polychronidou

Transaction Report:

(Note: With the exception of the correction of typographical or spelling errors that could be a source of ambiguity, letters and reports are not edited. Depending on transfer agreements, referee reports obtained elsewhere may or may not be included in this compilation. Referee reports are anonymous unless the Referee chooses to sign their reports.)

1st Editorial Decision **3rd Mar 2020**

Thank you again for submitting your work to Molecular Systems Biology. We have now heard back from the three referees who agreed to evaluate your study. As you will see below, the reviewers raise substantial concerns, which unfortunately preclude the publication of the study in its current form.

All three reviewers raise significant concerns regarding the conclusiveness of the study and point out that in absence of substantial follow up analyses the ability of progulonFinder to predict protein function and its broader relevance and superiority compared to alternative approaches remain are not well supported. They also mention that given your previous study (Kustatscher et al, 2019), the conceptual novelty of the work in its current form seems somewhat limited. The reviewers nevertheless acknowledge that the presented findings seem potentially interesting and all three of them provide quite constructive and detailed suggestions on how the study could be revised and extended in order to address their concerns and make it suitable for publication in MSB. Taken together and considering that the reviewers think that the work is potentially relevant, we would like to offer you a chance to address the issues raised in a major revision. As the reviewers' recommendations are rather clear, I think that there is no need to repeat any of the points raised.

All issues raised by the reviewers need to be satisfactorily addressed. As you may already know, our editorial policy allows in principle a single round of major revision, so it is essential to provide responses to the reviewers' comments that are as complete as possible. I understand that the required revisions are substantive. Please feel free to contact me in case you would like to discuss in further detail any of the issues raised or if you would like to share your revision plan with me.

On a more editorial level, we would ask you to address the following:

Reviewer #1:

Kustatscher et al. presented a bioinformatic approach/ analysis on "protein regulon" or progulons. This approach is built on ProteomeHD, their previous work published at Nature Biotechnology (Ref. 27, please update the bioRxiv citation). Although ProteomeHD already heavily studied co-regulation of proteins using quantitative data of thousands of proteins to 294 cell perturbations, the authors suggest that Progulon analysis, due to usage of a supervised learning approach, captures large co-regulation modules that are functional. They first applied OPTICS, a clustering algorithm, and divided the proteome into 41 seed groups. They then retrieved 41 "Progulon" (i.e., relevant protein lists to each seed) through ProteomeHD data using seeding lists. Mapping these lists to an independently published dataset of 36 breast cancer cell lines which has both mRNA and protein data, they found the majority of the Progulons (24 vs.3 in 41) have low mRNA-to-protein correlation, but the co-variation (i.e., coordination) of mRNA and proteins respectively within each Progulon seems high. They mapped to the Non-exponential protein degradation (NED) list and found the NED extent in a Progulon negatively impacts mRNA-protein correlation. Following, they presented a targeted search example using a list of 41 DNA replication proteins and retrieved 212 proteins. They used RNA screens and validated 10 of 20 (50%) proteins as potential candidates for DNA replication. Finally, they showed diverse perturbation in ProteomeHD is important to find correct proteins for a Progulon.

In general, this reviewer has no doubt that a following-up tool after NBT paper which extends the specific search of "one protein" to "a list of proteins" in ProteomeHD for fishing out co-varying proteins is useful. However, the current analysis, data interpretation, and logic flow need significant improvements. Especially the author failed to consider a few alternative explanations, which, if true, will jeopardize many of their major conclusions.

Major:

1. The definition of Progulon. In the first breast cancer dataset, the author seems to claim that those 41 progulons extracted from ProteomeHD are stable and presenting "high-order modular regulation of the proteome" (see their title), i.e., progulon is a biological concept with lists. However, the targeted search using the list of DNA replication proteins suggested that a 'Progulon" can be extracted with any list of proteins, i.e., progulon is not stable.
Essentially, this number of 41 for progulons was almost random - it was defined by OPTICS clustering result. It is unknown why other clustering methods cannot be used, so that one may come up with another number of protein lists for fishing out "Progulon".
In simple words, it seems that "Progulon" is a touted concept, which is highly dynamic, depending on the input list. The stability of Progulon lists has to be established under the current title and core message the paper aims to deliver. For example, if the authors only use half of ProteomeHD samples or only the new breast cancer dataset, will they see the same/ similar "Progulons"? How robust is OPTICs result compared to other clustering algorithms?

2. The supervised learning approach. It is not clear to biologists how the score cutoff of 0.5 (RF score) in Progulon is decided. The author may want to refer to String, where a network connectivity is always compared to a random list for any given lists, with a P value reported. A relevant question is that, what if some user come up with a random list of proteins- will progulon produce reliable result (e.g., no relevant proteins will be identified)? It seems that the cutoff score should be tested for different list sizes and derived with random permutations. If this is already done, please fully describe it.

3. Orthogonal, external validation of Progulon results. Before analyzing the mRNA and protein variation and co-variation patterns, the author should consider whether an external resource such as STRING, or BioPlex Interactome can be to validate the progulon in large-scale. I appreciated that detailed discussion about ATP synthesis progulon and the impressive validation experiments on DNA replication proteins. But one could still argue that both ATP synthesis and DNA replication are two selected examples that are biologically distinctive and well-known to impact the proteome broadly.

4. The impact of ribosome and mitoribosome progulons in whole data interpretation. The author reported several significant observations, including the negative correlation between mRNA/ protein changes and mRNA-protein correlation (PCC), and also those correlations regarding NED proteins (Figure 2 d, e, h; Figure S4c). However, as the author mentioned, the ribosome and mitoribosome are two dramatic outlies causing these correlation trend. The alternative explanation is that only these two progulons are following the mechanisms that the authors proposed. This reviewer is not surprised at all (see below) that protein coordination negatively correlates to PCC (Figure 2e), but indeed surprised about Figure 2d, i.e., mRNA coordination also negatively correlates to PCC. First of all, it is difficult to speculate such a biology mechanism that controls mRNA to reduce PCC. Secondly, a closer look at Figure 2e only suggests ribosomes and proteasome are outliers. Removing them will ends up with no correlation or even positive correlation for other progulons. This is an essential point that the authors need to address/revise before the manuscript being considered for MSB publication.

5. An alternative explanation for NED related conclusions. The conclusions that non-exponential protein degradation explains "coordination vs contribution paradox" are interesting, but not convincing. NED is a quite new concept. And the authors explanations (Page 7) mixed up protein degradation, NED, and protein complex formation relationships. It is already well-documented by lot of literature that protein complexes are co-regulated in many conditions (i.e., which explains coordination of proteins), and that the active degradation of excessive copies of complex subunits causes the low PCC. To fully establish their theory, the authors need to show that those NEDs which are not participating protein complexes similarly explained the paradox, or at least, to exclude the contribution from protein complex organization.

6. Following, the discussion about "starting materials" of mRNA, NED, and protein coordination is too speculative (Para.2, Page 11) and should be removed, unless the above issues are solved.

7. Extended Figure 4 and "random 15" experiments. The author made an interesting comparison of data redundancy by using 15 NCC samples only, not using them but all the other samples, and using random 15 samples. This intriguingly suggests that one could rely on ProteomeHD to inspect the potential protein relationships without the need to perform some NEW experiments. However, in their results (Figure E4), even the "random 15" experiments succeeded to distinguish replication-related proteins and mitochondria proteins. It is therefore not convincing to rely on the case of NCC experiment (only), because the DNA replication seems to be globally affected in all ProteomeHD samples. The authors should consider to include more lists (e.g., using the breast cancer dataset, to observe whether the breast cancer signatures can be acquired or practically acquired etc from ProteomeHD without breast cancer dataset, or random 36 samples). Right now, the data leads to the impression that NCC experiment tends to only report false positive hits.

Minor:
1. The raw score of RF and the numbers in each box should be also shown for Figure E4 (e.g., only for n=15 comparisons), so that one can compare the relevance of NCC experiment in DNA replication.

2. The author may want to advocate the usage of Progulon proteins (names in Table S7) as gene sets for e.g., GSEA analysis.

3. Line 260. Please define protein isoforms, as it suddenly showed up.

4. Line 263. This sentence is not clear and can be miss-interpreted.

Reviewer #2:

Summary:

In the manuscript entitled 'Higher order modular regulation of the human proteome', Kustatscher and colleagues build on their previous work establishing the ProteomeHD resource and additionally develop a random forest algorithm to mine it. They develop a publicly accessible pipeline capable of identifying modules of co-regulated proteins in a semi-supervised fashion. They further investigate the mechanisms of gene expression regulation underlying 'progulon' regulation. Finally, they attempt to demonstrate the power of their approach in predicting gene function using a small siRNA validation screen of candidate genes involved in DNA replication and investigate how the structure of their source database affects gene function prediction.

As a whole, the results presented in this paper are interesting. However, they are somewhat incremental given the previous work by the same authors. Specifically, the proteomeHD paper (Kustatscher et al., Nat. Biotechnol., 2019) already makes the point that the co-regulation data can be used to infer protein function. The current manuscript is largely based on the same data source and "just" presents a novel tool "progulonFinder" to mine it. So the conceptual novelty is quite low.

My second major critique is that the breadth and depth of the functional followup of the authors' predictions are currently insufficient: The presented data from siRNA screens does not support the conclusion that progulonFinder can indeed predict protein function. The statement in the abstract that they identified "at least ten new replication factors as confirmed by extensive siRNA screening" is not supported by the data presented. Therefore, the "Adequacy of methods analysis and validation" is currently low.

Major comments and suggestions
The results of the siRNA screen against proteins involved in DNA replication are not convincing: In total, the authors tested 25 genes, 20 "new candidates" and 5 "low RF score controls". In case the "new candidates" were really enriched in factors mediating DNA replication there "combined siRNA screen score" would be expected to be significantly higher. 11 genes fall into the "high confidence" set, 10 of them "new candidates" and 1 "low RF score control". This gives a hypergeometric p-value $P(X >= 10)$ of 0.24, which is far from a reasonable significance level. The authors argue that the "low RF score" hit in the high confidence set (LGALS3) is actually involved in DNA replication based on a previous publication. While this is true, it still means that low and high RF score proteins do not behave significantly differently in the genetic screen. Importantly, even if LGALS3 would be removed from the data, the hypergeometric test would not show a significant enrichment of "new candidates" in the "high confidence" subset. The main problem of the screen seems to be that too few "low RF score" genes were included. As it stands, the data does not support the statement that progulonFinder can identify new proteins involved in DNA replication. The validation of predictions could be much improved if they investigated how these candidates perform in other, more comprehensive, genome-wide, cell cycle/DNA damage related screens assessed either flow cytometrically (https://doi.org/10.1016/j.molcel.2009.06.021) or microscopically (MitoCheck - https://www.mitocheck.org/screens_browser.shtml). Functional validation of further progulon candidate members in other pathways (e.g. secretion or DNA damage - see MitoCheck or other resources) would further strengthen the case.

Line 372: "We demonstrate that even for a well-studied process such as DNA replication, new factors can be identified with high accuracy (~75% validation rate)." As stated above this alleged high accuracy is actually not statistically significant.
Line 374": "While our work was in progress, replication-related functions for three of our predictions were independently confirmed: ATAD2 , FTO and ZMYM3." The corresponding publications date back to 2016 and 2017. Therefore, the argument that these were independently confirmed while the "work was in progress" is somewhat weak.

Progulon-specific explorations regarding perturbations: The authors clearly explore the fact that the nature of the perturbations encompassed by the ProteomeHD database is not as important as their sheer number. While this may be true, this reviewer feels that the functional impact and novelty of the study could be improved by exploring the perturbations whereby co-regulation of a progulon becomes weaker. For instance, in Figure 1C to E (lower panels), the 'seed' groups of proteins tend to follow very closely to the novel interacting candidates of the progulons. Nonetheless, there are clearly perturbations which lead to a breakdown in this relationship (i.e. when the magenta and blue lines part from one another). Can any conclusions be drawn from focusing on these specific conditions? For instance, do some perturbation specifically decouple potential post-transcriptional regulations between different parts of the progulon? Can the authors elaborate on potential mechanisms behind this?

Along the same lines as comment 4), it would be informative to have a heatmap displaying the coordination of each progulon as a function of perturbation (as in Romanov et al. 2019 Cell https://doi.org/10.1016/j.cell.2019.03.015 Figure 3) either in the supplement or in the main figures.

Minor comments and suggestions
The authors should revise their figure annotations (e.g. there is no annotated Supplementary Figure 2C)

The relationship between protein variation of a progulon and its respective protein-mRNA PCC seems a bit circular: it is an exercise in comparing the covariance of two variables to the variation in one of those variables. In any case, it would be interesting for the authors to always outline with colours which of these progulons correspond to mitochondrial and cytoplasmic ribosomes, given that these seem to always be the outliers (in Supplementary Figures 2, 3 and 4)

In figure 1h, several of the progulons seem to have overlapping members with one another ('off diagonal' clusters). The authors could perhaps elaborate on these cross-pathway cameos and whether or not they are expected based on the literature.

The authors underline how the average half-lives of mRNA and proteins seem to correlate for progulons, with entities such as the proteosome displaying high half-lives. How about the coordination of mRNA and protein half-lives within progulons? Do these follow the same trends as steady state abundances?

Supplementary Figure 2 b: The axis label and legend is uses the term "median MAD", which means "median median absolute deviation", so "median" is redundant.

Reviewer #3:

The authors of the manuscript used human proteome data across 294 perturbations to train a model for the prediction of protein co-expression clusters. The output of their model is a set of 41 protein regulons ('progulons') that are significantly co-regulated in terms of their protein abundances.

The authors then use transcriptomic and proteomic data from cancer cell lines to try and determine whether the 41 progulons are co-ordinated at the level of mRNA expression (transcription and degradation) or at the protein level (synthesis and degradation). They later divide their progulon proteins into exponentially degraded (ED) and non-exponentially degraded (NED) proteins to try and account for the patterns they observe.

The authors have made their random forest (RF) classifier available in the form of a webtool (progulonFinder). They use this to predict new components of the replisome as a case study. For validation, RNAi screens for replication-linked phenotypes were then performed on 20 new replisome candidates.

Main conclusions and concepts:

• Identification of 41 protein co-regulation clusters in the human proteome, covering ~4000 proteins. The prediction tool has been made publicly available for re-use.
• Progulon proteins tend to have a lower mRNA-to-protein correlation than the proteome average, indicating a role for regulation after transcription.
• Finding of no positive relationship overall between mRNA co-expression (co-ordination) within progulons and the mRNA-to-protein correlation (contribution).
• Progulons are enriched for non-exponentially degraded proteins (NEDs), which are often regulated by proteasome-mediated

degradation. This may explain the discord between co-ordination and contribution.
• Prediction of new replisome components as a proof-of-principle, with an attempted validation using RNAi-based screens of replication-linked phenotypes for 20 candidates.

General comments:

This manuscript follows on from a paper published recently in Nature Biotechnology (Kustatscher et al., 2019). Relative to that paper, the authors try to establish novelty here by using a supervised learning approach to generate co-regulated clusters instead of an unsupervised or correlation-based approach. However, they do not demonstrate for this dataset (proteomeHD) the effectiveness of this method over and above unsupervised or correlation-based approaches.

In the second part of the manuscript, the authors use transcriptomic and proteomic data to probe the mechanism (transcriptional vs. post-transcriptional/post-translational) underlying protein co-regulation. The enrichment of progulon proteins for non-exponentially degraded proteins (NEDs) is used to help explain the lack of an expected correlation between co-ordination and contribution (Figure 2h). While potentially interesting, I do not find their conclusions wholly convincing given problems with the underlying data and statistical analysis.

Their RNAi-based validation screen for new replisome components provides reasonable support for the results of progulonFinder

Overall, this research has the potential to reveal some useful insights into protein co-regulation and the mRNA-to-protein relationship. However, the novelty of this research and the extent to which the data support the conclusions are questionable. The NED-associated analysis in particular (Figure 2H) is unconvincing.

Major points:

• The authors emphasise in the manuscript that progulon control is predominantly 'post-transcriptional'. However, this term refers to all steps between transcription and translation and not simply all events after transcription. The proteasome-mediated degradation of proteins is a post-translational mechanism, for example. This confusion should be corrected throughout the manuscript.
• The authors use a supervised learning approach to identify progulons from the seed proteins in clusters. In principle, similar results could be obtained by iteratively retrieving highly correlated/co-regulated protein pairs for each protein in the seed set and then taking the union. This could be achieved using the existing proteomeHD resource and using a correlation/co-regulation threshold producing similar sample sizes to the progulons from progulonFinder. This analysis should be performed and the results benchmarked alongside those of progulonFinder for functional relevance (using GO enrichments and Reactome, for example). This is necessary to establish the novelty/effectiveness of progulonFinder relative to the Kustatscher et al., 2019 paper.
• Figure 1g shows that some progulons have an overlap as high as 80%. It is not surprising therefore that such progulons will have highly correlated expression profiles if they share components. Figure 1g seems to mirror figure 1h for this reason. The average (mean or median?) correlation should therefore be recalculated after excluding self-self (r=1) correlations. The statement 'Expression changes of functionally similar progulons are correlated (Fig. 1h)' is not currently supported by the data for this reason.
• For the GO enrichment analysis in Fig. 1I, GO enrichments should be performed for completely random progulons of the same size to determine if summing the number of GO terms observed within each progulon is meaningful. The results can be included as a supplementary figure.
• Seemingly arbitrary decisions are often made when it comes to the statistical analysis. For example, Spearman's correlation is used in Fig. 1H but Pearsons's for Fig. 2A-C. Also, correlation coefficients are calculated in Figs. 2 D-E but not in Fig 2C? Inconsistencies like this should be justified.
• Much of the analysis for Figure 2 has been repeated for two additional datasets (Supplementary Figure 3 and Supplementary Figure 4). The claim that the differences between results is 'minor' is highly dubious. The median mRNA-protein correlation in Supplementary Figure 3 for example is only 0.15. There also appears to be no correlation in the figure between mRNA coexpression and protein coexpression. The enrichment of progulons for low mRNA-to-protein correlations (implying regulation after transcription) also seems to disappear in this figure. Also, the negative correlations found in Fig 2D and Fig 2E seem to disappear in supplementary figures 3 and 4. These are significant deviations that undermine some of the conclusions in the main text.
• The authors should also state in the main text that the results for supplementary figure 4 derive from one-to-one ortholog mappings between human and mouse for each progulon, and link this to the observation in Figure 2.I that only ~30% of co-expressed human proteins within a progulon are also co-expressed in mouse. This brings into question the appropriateness of using this data set as a means to validate the Figure 2 results.
• For all of the correlations given in Figure 2H, the regression lines seem to be heavily influenced by the two outliers (orange dots). Little-to-no correlation is apparent by eye for the remaining data points. The analysis has been repeated without the outliers in supplementary figure 5c, but the data for breast cancer cells has not been plotted (as in Supplementary figure 5b). This data should be plotted explicitly and r or R2 values displayed in the plots. The relevance of [NED%] as an explanatory variable should then be re-evaluated in the main text, given the weak r/R2 values calculated after outlier exclusiom.

• The claim is also made that the trend observed in Figure 2H (bottom-left) persists even after the exclusion of outliers. However, this is only the case for the mouse tissues and not the LCL cells. In fact, 3/6 of the correlations tested in Supplementary Figure 5b become non-significant after the outliers are excluded. This should be stated in the main text, and again undermines some of the conclusions in the main text. The conclusions given should be re-evaluated in light of these results.
• The authors identify ~4,000 proteins that belong to progulons. This leaves ~15,000 proteins that cannot be assigned to co-regulatory modules. The authors should comment in the discussion whether they believe this represents a biological reality or can be accounted for by data/statistical limitations. The stringent AUC threshold (AUC>0.99) adopted for progulon filtering may be relevant here.

Minor points:

• For figure 1 C-E, please explain in the legend your criteria for selecting the 25 different experiments shown in the line plot. Were they selected at random?
• It should be explained somewhere exactly how the percentage overlaps in Fig. 1G were calculated. The authors should also explain how missing data was handled when calculating the Spearman's correlation in Fig. 1H.
• Line 152→ 'In contrast to bacterial and eukaryotic operons, we find no substantial clustering of progulon gene in terms of chromosome location'.
• On line 174, indicate the significance threshold used for the test.
• The negative correlation found for Figure 2D lies above the standard significance threshold ($p = 0.064 > 0.05$) and seems to be determined strongly by the 'Ribosome' data point. This should be stated in the main text.
• The manuscript refers to a 'Supplementary Figure 2C.', but this does not seem to exist.
• In relation to Figure 2H (top-left) please state the number of progulons that lie significantly above and below the 10% threshold.
• The presentation of the phenotype data for Figure 3 is confusing. I think a brief description of the Figure 3E phenotypes should be given in the legend and also in the legend of supplementary figure 7 for all phenotypes listed to aid non-specialists. It is also not clear to me why 20 phenotypes are tested and yet the maximum siRNA score given is 13 and not 20? The green/yellow/purple color scheme used in Figure 3e and figure 8 also does not seem to correspond to the color scheme used in supplementary figure 7 as the colors represent different concepts. This is very confusing. In general, figure legends for supplementary figure 7 and supplementary figure 8 should be more detailed. It is also not clear if the results in figure 3f derive from the RSA- or SD-based scoring metric.
• For all figures and supplementary figures where a correlation has been calculated, state whether this is a Spearman's or Pearson's correlation coefficient if this has not already been shown in the figure.

Higher-order modular regulation of the human proteome
Kustatscher, Hödl-Baumgartner, Grabowski, Groth and Rappsilber
MSB-20-9503

RESPONSE TO THE REVIEWERS

We would like to thank the reviewers for taking the time to read our manuscript and for their constructive feedback. We would also like to apologise for the exceptionally long time it has taken us to address these comments. This was in part due to a temporary focus on Covid-related research in our group and in part due to the first author starting an independent position while working on these revision tasks. However, we are now happy to present a significantly re-worked and improved version of our manuscript. The major changes to the manuscript include the following:

- Improved the way we detect strongly co-regulated core modules by implementing a double-clustering approach
- Implemented a novel connectivity score to determine biologically relevant cut-offs for the machine-learning-based module predictions
- Removed data on non-exponential protein degradation as a potential mechanism by which progulons are regulated, due to it being considered weak evidence by two reviewers
- Re-wrote parts of the manuscript, especially the results section, and re-generated 11 of 17 figures to reflect these changes

Please find our point-by-point response to the reviewers below.

Reviewer #1

Kustatscher et al. presented a bioinformatic approach / analysis on "protein regulon" or progulons. This approach is built on ProteomeHD, their previous work published at Nature Biotechnology (Ref. 27, please update the bioRxiv citation). Although ProteomeHD already heavily studied co-regulation of proteins using quantitative data of thousands of proteins to 294 cell perturbations, the authors suggest that Progulon analysis, due to usage of a supervised learning approach, captures large co-regulation modules that are functional. They first applied OPTICS, a clustering algorithm, and divided the proteome into 41 seed groups. They then retrieved 41 "Progulon" (i.e., relevant protein lists to each seed) through ProteomeHD data using seeding lists. Mapping these lists to an independently published dataset of 36 breast cancer cell lines which has both mRNA and protein data, they found the majority of the Progulons (24 vs.3 in 41) have low mRNA-to-protein correlation, but the co-variation (i.e., coordination) of mRNA and proteins respectively within each Progulon seems high. They mapped to the Non-exponential protein degradation (NED) list and found the NED extent in a Progulon negatively impacts mRNA-protein correlation. Following, they presented a targeted search example using a list of 41 DNA replication proteins and retrieved 212 proteins. They used RNA screens and validated 10 of 20 (50%) proteins as potential candidates for DNA replication. Finally, they showed diverse perturbation in ProteomeHD is important

to find correct proteins for a Progulon.

In general, this reviewer has no doubt that a following-up tool after NBT paper which extends the specific search of "one protein" to "a list of proteins" in ProteomeHD for fishing out co-varying proteins is useful. However, the current analysis, data interpretation, and logic flow need significant improvements. Especially the author failed to consider a few alternative explanations, which, if true, will jeopardize many of their major conclusions.

We thank the reviewer for this principally positive assessment of our manuscript, and will address their specific comments in detail below. Ref. 27 has been updated.

Major:
1. The definition of Progulon. In the first breast cancer dataset, the author seems to claim that those 41 progulons extracted from ProteomeHD are stable and presenting "high-order modular regulation of the proteome" (see their title), i.e., progulon is a biological concept with lists. However, the targeted search using the list of DNA replication proteins suggested that a 'Progulon" can be extracted with any list of proteins, i.e., progulon is not stable.
Essentially, this number of 41 for progulons was almost random - it was defined by OPTICS clustering result. It is unknown why other clustering methods cannot be used, so that one may come up with another number of protein lists for fishing out "Progulon".
In simple words, it seems that "Progulon" is a touted concept, which is highly dynamic, depending on the input list. The stability of Progulon lists has to be established under the current title and core message the paper aims to deliver. For example, if the authors only use half of ProteomeHD samples or only the new breast cancer dataset, will they see the same / similar "Progulons"? How robust is OPTICs result compared to other clustering algorithms?

We agree that this comment raises important questions about both conceptual and technical aspects of our work. We have addressed these by expanding the discussion section (also outlined below) and by introducing a new clustering approach and a new scoring system.

About the conceptual concerns:
The conceptual questions raised here are (a) if large co-regulation modules (“progulons”) are stable regulatory units of the proteome, and (b) how many of these modules exist. At first sight, there is an apparent discrepancy between the biological concept of stable modules and the technology we use to detect them: supervised machine-learning. Supervised machine-learning (in our case the “progulonFinder” workflow) can be executed with any set of input lists (training data), so theoretically one could detect an infinite number of modules. However, not all input lists will yield meaningful results. Searching with a random input list does not return a progulon. Searching with similar input lists will return similar results.

We cannot detect progulons using clustering alone, because clustering only detects relatively small, tightly co-regulated modules such as protein complexes and fails to scale accurately to larger modules. Supervised machine-learning, on the other hand, creates a model based on input / training lists which can capture complex co-regulation modules much more accurately than clustering. The more components of the module one uses as an input list, the more accurately and comprehensively the results reflect the underlying module. For example, using

the 5x RFC and 4x GINS proteins separately as input lists will return some parts of the "DNA replication module" that would be upregulated in replicating cells. Manually refining that input list to contain all replisome factors just helps to capture the underlying module more accurately and / or comprehensively. This means that by using different, related input lists we are not detecting entirely different progulons, but we are refining the detection of the same progulon.

One way to address this issue is to measure the "connectivity" of a progulon module, i.e. to test if we are capturing genuine modules or just random sets of proteins co-regulated with the training list. We have now introduced a new score that reflects how interconnected the proteins assigned to a progulon are.

Related to this is the question of how many progulons exist. It is difficult if not impossible to state their exact number or protein composition. Very much like the definition of protein families based on multiple sequence alignments, better algorithms and more/better data will affect the number of assigned progulons and also their precise composition. This does not diminish the fact that progulons appear to exist and that they provide a novel entry point into discovering protein functions. We use clustering to define a set of tightly co-regulated proteins as input lists, or seed proteins, so the number of progulons we detect depends on the output of that clustering analysis. As the reviewer rightly suggests, the choice of clustering algorithm / setting will directly affect the number of seeds / progulons we identify. We have now refined our clustering approach as follows:

A refined clustering approach to define seed lists:
Previously we only used a density-based clustering algorithm, OPTICS, leading to the detection of 41 progulons. We now tested the use of two additional types of clustering algorithms: hierarchical and graph clustering. We applied hierarchical clustering through the widely used WGCNA package, which also contains specialised strategies for cluster calling, i.e. dividing the continuous cluster dendrogram into a defined set of clusters. However, as our new Appendix Fig S1 shows, hierarchical clustering was not as successful as OPTICS at capturing functionally related groups of proteins. In contrast, we found that a graph clustering algorithm, clusterONE, did so very well. OPTICS and clusterONE produced similar outcomes despite the fact that they are based on completely different mathematical principles (density vs graph based) and using different input data (entire ProteomeHD protein-protein association matrix vs 0.5% top-scoring associations only). One key difference between these two approaches is that clusterONE results are redundant, whereas OPTICS clusters are not. We therefore decided to proceed as follows: for each OPTICS cluster we identify the best-matching clusterONE cluster (= the one with the most overlapping proteins) and then keep only the proteins that are shared between OPTICS and clusterONE. In this way, we identify 72 core modules that have been identified as groups by two different clustering algorithms.

After using these 72 seed groups as input lists of the progulonFinder we apply our previously established filtering criteria (e.g. area under ROC curve > 0.99) and are left with 41 progulons, as in the previous OPTICS-only version of the manuscript. However, after increasing the stringency of our filtering criteria further and introducing a requirement for progulons to pass a "connectivity score threshold" (see question 2 of reviewer #1), this number was further reduced to a final set of 31 progulons in the revised version of the manuscript. This

is not to be viewed as the absolute number of progulons that exist. This is the number of progulons that we detect. Not being able to define an absolute number of progulons that exist in human cells appears to us not speaking against the validity of the concept. Examples of accepted concepts without absolute numbers despite decades of research include protein families (which like progulons base exclusively on computational methods being applied to experimental data) and protein complexes.

Using subsets or alternatives to ProteomeHD as input data

Following the suggestion of the reviewer we tested if splitting ProteomeHD in halves or thirds would allow us to assess how stable the detected progulons were against different input data. However, we found that all of these settings produced results that were significantly inferior to using ProteomeHD as a whole. For example, on the entire ProteomeHD (294 SILAC ratios), OPTICS clustering identifies one cluster consisting of 15 co-regulated subunits of the ATP synthase complex. When ProteomeHD is split into two random halves (147 SILAC ratios each), then the same clustering approach identifies clusters containing only 7 and 10 subunits of the same complex, respectively. While missing out on some genuine ATP synthase subunits, these smaller clusters obtained from ProteomeHD halves both contain the “Transmembrane emp24 domain-containing protein 2”. This may be functionally related to the ATP synthase in a as-of-yet unknown way, but it is very unlikely to be a genuine subunit of this well-characterised complex. From this and other examples we concluded that splitting ProteomeHD in two or three parts reduces the quality of the results overall and is therefore not suitable to validate them.

Similarly, we aimed to test the approach against a completely different input data set. The breast cancer data set mentioned by the reviewer encompasses 36 cell lines, about 13% of ProteomeHD, which is too small. A more recently released dataset by Nusinow *et al* (Quantitative Proteomics of the Cancer Cell Line Encyclopedia, Cell, 2020) covered protein expression in 375 cancer cell lines. We therefore applied the progulonFinder workflow to this CCLE data set using the same set of seed proteins used for our main ProteomeHD-based approach. Intriguingly, in this case we had to relax our filtering criteria (AUC < 0.90) for a comparable number of progulons to pass through our filters, and those that did pass appear to reflect the underlying biology less well. For example, as shown by **Reviewer figure R1**, the ATP synthase progulon captured in the CCLE data lacked many proteins that were detected using ProteomeHD. Although some proteins were exclusively detected as co-regulated with the ATP synthase by the CCLE data, the vast majority of these were not directly associated with the respirasome (**Reviewer figure R1**). For example, these included proteins of the mitochondrial ribosomes or mitochondrial import machinery, which are biologically not directly related to the

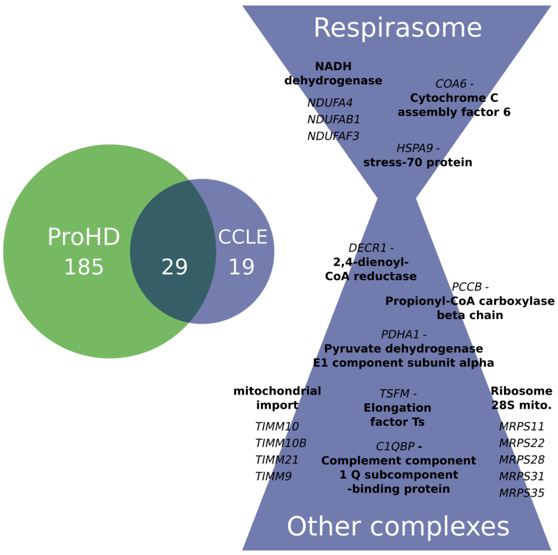

Reviewer figure 1: Comparison of ATP synthase progulon based on the ProteomeHD and CCLE data sets. Note that many CCLE specific hits are not directly related to respiration.

ATP synthase. In contrast, most proteins in the ProteomeHD-based ATP synthase progulon are much more directly related to its biological function, as illustrated in Figure EV1 and Dataset EV3.

We are not sure why ProteomeHD outperforms the CCLE data set for progulon prediction. It could be that SILAC quantitation is more accurate or less susceptible to batch effects than TMT quantitation. It is also possible that this is due to the variety of biological conditions present in ProteomeHD, which contains not just different cell lines, but also various drug and growth factor treatments, for example.

Taken together, we conclude that different clustering approaches could validate our strategy to find seed proteins in ProteomeHD. However, we cannot use different input data to validate progulons, because the presently available choices are underpowered compared to ProtomeHD. Future releases of ProteomeHD will hopefully be big enough to be split and deliver such additional insights.

> 2. The supervised learning approach. It is not clear to biologists how the score cutoff of 0.5 (RF score) in Progulon is decided. The author may want to refer to String, where a network connectivity is always compared to a random list for any given lists, with a P value reported. A relevant question is that, what if some user come up with a random list of proteins- will progulon produce reliable result (e.g., no relevant proteins will be identified)? It seems that the cutoff score should be tested for different list sizes and derived with random permutations. If this is already done, please fully describe it.

We thank the reviewer for this suggestion. In the new version of our manuscript, the Random Forest (RF) score cut-off for co-regulation modules is no longer fixed at 0.5. This value was dictated by the RF algorithm itself, which classifies any proteins above that score to belong to the "co-regulated class". However, this only takes into account whether proteins are co-regulated with the training proteins and not whether progulon proteins are co-regulated with each other, i.e. are forming a genuine co-regulation module. To address this we now also calculate a *connectivity p-value*, which is used to select the appropriate RF score cut-off such that the resulting module contains proteins that are significantly co-regulated with each other. To do so, we now compare progulons to a co-regulation network created with all proteins in the analysis. Using a Fisher's Exact test we calculate a p-value that reflects if a progulon is enriched for protein pairs that are among the top 0.5% of co-regulated protein pairs in the overall network. This calculation is performed at a series of RF score cut-offs going from 0.5 to 1.0 in 0.01 increments. We then chose the minimum RF score cut-off that creates a significantly inter-connected module. For ~80% of the progulons, the default RF score cut-off of 0.5 already creates a module with a connectivity $p\text{-value} < 0.05$. For the remaining progulons, the new approach resulted in more stringent cut-offs ranging from 0.51 to 0.57 and one progulon had to be discarded completely, because no RF score cut-off resulted in a significantly inter-connected module.

Note that our workflow already contained other quality control steps to ensure that only high-quality modules were included in the final report (e.g. a requirement for the cross-validated training data to give an area under ROC curve above 0.99 and the need for the training proteins themselves to receive high cross-validated RF scores). We increased the

stringency of these settings further, which resulted in an overall decrease in the number of progulons from 41 to 31.

Finally, we tested our filtering criteria using a set of randomised seed modules, as suggested by the reviewer. We used the same number of random groups of proteins as we had used for the real core modules and these also had the same size distribution (between 4 and 82 proteins). None of the random modules made it through the first of our filtering criteria (area under ROC > 0.99). This has been added to the results section.

> 3. Orthogonal, external validation of Progulon results. Before analyzing the mRNA and protein variation and co-variation patterns, the author should consider whether an external resource such as STRING, or BioPlex Interactome can be to validate the progulon in large-scale. I appreciated that detailed discussion about ATP synthesis progulon and the impressive validation experiments on DNA replication proteins. But one could still argue that both ATP synthesis and DNA replication are two selected examples that are biologically distinctive and well-known to impact the proteome broadly.

We provide three layers of orthogonal, external validation of our approach. First, we manually annotate three progulons based on existing literature (one of which, the ATP synthase progulon, in high detail). Second, for a more systematic approach, we test each progulon for GO term enrichment. In response to this question we now added two additional dimensions, showing that progulons are also enriched for Reactome pathways and protein complexes. This is shown in the new Figure 1H. Please note that we could not use STRING and BioPlex for this because those report pairwise interactions, not protein groups. Third, we conducted the siRNA screen for the DNA replication progulon to experimentally validate previously unknown associations within a co-regulation module.

> 4. The impact of ribosome and mitoribosome progulons in whole data interpretation. The author reported several significant observations, including the negative correlation between mRNA/ protein changes and mRNA-protein correlation (PCC), and also those correlations regarding NED proteins (Figure 2 d, e, h; Figure S4c). However, as the author mentioned, the ribosome and mitoribosome are two dramatic outlies causing these correlation trend. The alternative explanation is that only these two progulons are following the mechanisms that the authors proposed. This reviewer is not surprised at all (see below) that protein coordination negatively correlates to PCC (Figure 2e), but indeed surprised about Figure 2d, i.e., mRNA coordination also negatively correlates to PCC. First of all, it is difficult to speculate such a biology mechanism that controls mRNA to reduce PCC. Secondly, a closer look at Figure 2e only suggests ribosomes and proteasome are outliers. Removing them will ends up with no correlation or even positive correlation for other progulons. This is an essential point that the authors need to address/revise before the manuscript being considered for MSB publication.

Following the improvements we made to our progulon detection approach the new Figure 2D,E looks slightly different, but the general trends are unchanged. Even without ribosome and proteasome progulons we find statistically significant negative correlations (**Reviewer figure R2)**. However, these correlations are indeed weak. Note that even a weak negative correlation is still an intriguing result, since one would intuitively expect a positive correlation in this case.

This is because if a progulon is strongly coordinated at the mRNA level one would expect that these changes are more or less directly translated into protein level changes, which they apparently are not. We have updated the text to reflect this, including the fact that it is driven by the proteasome and ribosome progulons. We have also removed NED as a potential explanation of these results.

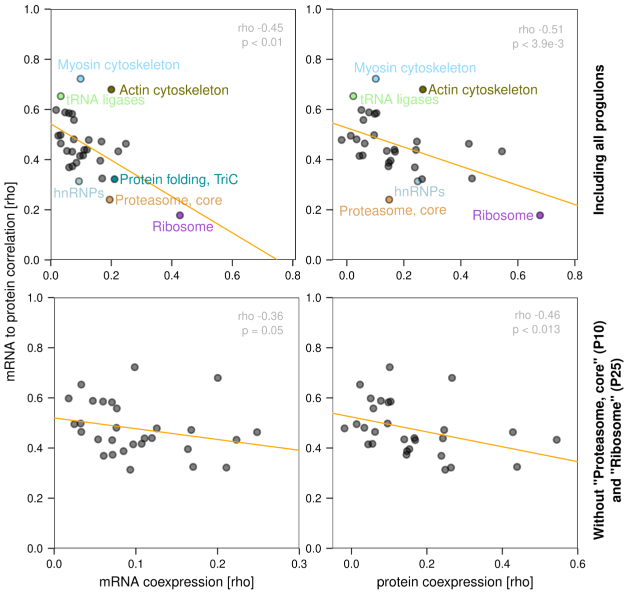

Reviewer figure 2: Comparison of figure 2D,E (top row) with a version of these panels where proteasome and ribosome progulons have been removed (bottom).

5. An alternative explanation for NED related conclusions. The conclusions that non-exponential protein degradation explains "coordination vs contribution paradox" are interesting, but not convincing. NED is a quite new concept. And the authors explanations (Page 7) mixed up protein degradation, NED, and protein complex formation relationships. It is already well-documented by lot of literature that protein complexes are co-regulated in many conditions (i.e., which explains coordination of proteins), and that the active degradation of excessive copies of complex subunits causes the low PCC. To fully establish their theory, the authors need to show that those NEDs which are not participating [in] protein complexes similarly explained the paradox, or at least, to exclude the contribution from protein complex organization.

In our manuscript we show that the mRNA and protein expression changes of many progulons are poorly correlated. The previous figures 2H and S5 suggested that at least some of that discrepancy could be explained by non-exponential degradation (NED) of excess protein copies. However, we agree with reviewers #1 and #3 that the evidence for this is still under-developed. We have therefore taken the decision to remove the reference to NED as explanation for the lack of mRNA - protein correlation from the results section. However, we now refer to this possibility in a short paragraph of the discussion section.

> 6. Following, the discussion about "starting materials" of mRNA, NED, and protein coordination is too speculative (Para.2, Page 11) and should be removed, unless the above issues are solved.

We have removed all references to NEDs from the result section and completely reworked this paragraph in the discussion.

> 7. Extended Figure 4 and "random 15" experiments. The author made an interesting comparison of data redundancy by using 15 NCC samples only, not using them but all the other samples, and using random 15 samples. This intriguingly suggests that one could rely on ProteomeHD to inspect the potential protein relationships without the need to perform some NEW experiments. However, in their results (Figure E4), even the "random 15" experiments succeeded to distinguish replication-related proteins and mitochondria proteins. It is therefore not convincing to rely on the case of NCC experiment (only), because the DNA replication seems to be globally affected in all ProteomeHD samples. The authors should consider to include more lists (e.g., using the breast cancer dataset, to observe whether the breast cancer signatures can be acquired or practically acquired etc from ProteomeHD without breast cancer dataset, or random 36 samples). Right now, the data leads to the impression that NCC experiment tends to only report false positive hits.

Our point is that leaving out the 15 NCC experiments has a worse impact on performance than leaving out 15 randomly chosen experiments. In this sense the NCC experiments are an important part of ProteomeHD. However, the lack of experimental diversity that comes with using the NCC experiments on their own means that the Random Forest algorithm struggles to distinguish replication-related from unrelated proteins. This also implies that one does not necessarily have to conduct targeted experiments to study co-regulation of a particular cellular process. We have now clarified this in the text. For breast cancer signatures this is different, as these link to the joined property of the group of samples (cancer) rather than the proteins.

> Minor:
> 1. The raw score of RF and the numbers in each box should be also shown for Figure E4 (e.g., only for n=15 comparisons), so that one can compare the relevance of NCC experiment in DNA replication.

We assume this comment refers specifically to the boxplots in Fig EV4C. The numbers per box have now been added to the figure. We are also now plotting the raw RF score rather than the quantile-normalised one.

> 2. The author may want to advocate the usage of Progulon proteins (names in Table S7) as gene sets for e.g., GSEA analysis.

We thank the reviewer for this suggestion. We have created lists of our progulons in the GMX format that makes them usable for GSEA analysis using the BROAD institute's tool at http://www.gsea-msigdb.org/gsea/index.jsp. We refer to this possibility in the discussion section and these files are also included in the manuscript as Dataset EV8.

3. Line 260. Please define protein isoforms, as it suddenly showed up.

This is referring to the fact that users may search with a UniProt ID that refers to a given gene, but in some cases ProteomeHD detects a specific splice form of that gene. We simplified this sentence, so that now it simply refers to ProgulonFinder automatically matching the query proteins to the correct protein IDs.

4. Line 263. This sentence is not clear and can be miss-interpreted.

We have paraphrased the sentence as follows: *ProgulonFinder identified 212 proteins that behave similar to known replisome components (Dataset EV6) in being similarly up- or downregulated in response to the 294 perturbations in ProteomeHD.*

Reviewer #2

Summary: In the manuscript entitled 'Higher order modular regulation of the human proteome', Kustatscher and colleagues build on their previous work establishing the ProteomeHD resource and additionally develop a random forest algorithm to mine it. They develop a publicly accessible pipeline capable of identifying modules of co-regulated proteins in a semi-supervised fashion. They further investigate the mechanisms of gene expression regulation underlying 'progulon' regulation. Finally, they attempt to demonstrate the power of their approach in predicting gene function using a small siRNA validation screen of candidate genes involved in DNA replication and investigate how the structure of their source database affects gene function prediction.

As a whole, the results presented in this paper are interesting. However, they are somewhat incremental given the previous work by the same authors. Specifically, the proteomeHD paper (Kustatscher et al., Nat. Biotechnol., 2019) already makes the point that the co-regulation data can be used to infer protein function. The current manuscript is largely based on the same data source and "just" presents a novel tool "progulonFinder" to mine it. So the conceptual novelty is quite low.

We are glad to hear the reviewer finds our results interesting, but we naturally disagree with the assessment that the conceptual novelty is low. There is a big conceptual difference between looking at pairwise protein-protein associations and studying modules of entire biological processes being regulated in a coordinated manner in the human proteome. As far as we are aware, the progulonFinder is also a unique tool that enables biologists with zero programming experience to use machine learning to detect such modules.

My second major critique is that the breadth and depth of the functional followup of the authors' predictions are currently insufficient: The presented data from siRNA screens does not support the conclusion that progulonFinder can indeed predict protein function. The statement in the abstract that they identified "at least ten new replication factors as confirmed by extensive siRNA screening" is not supported by the data presented. Therefore, the "Adequacy of methods analysis and validation" is currently low.

This is the major point of criticism raised by reviewer #2. Several of the subsequent questions are essentially variations of this issue. We therefore address it in a single comprehensive answer below.

> Major comments and suggestions
> The results of the siRNA screen against proteins involved in DNA replication are not convincing: In total, the authors tested 25 genes, 20 "new candidates" and 5 "low RF score controls". In case the "new candidates" were really enriched in factors mediating DNA replication there "combined siRNA screen score" would be expected to be significantly higher. 11 genes fall into the "high confidence" set, 10 of them "new candidates" and 1 "low RF score control". This gives a hypergeometric p-value P(X >= 10) of 0.24, which is far from a reasonable significance level.

At the core of the issue underpinning comments 1-4 appears to be the following question: do proteins that we predict to function in DNA replication receive significantly higher scores in the siRNA screen than proteins which we do not predict to function in replication? We agree that this is an important question. However, in our opinion the hypergeometric test above cannot be applied to test it, for the following reasons:

From a purely statistical perspective, the calculation above is simply underpowered. As shown in **Reviewer figure R3A,B**, if we multiplied all protein numbers by 10 the results would become highly significant (p = 8.01e-05). However, screening an additional 180 candidates would clearly be beyond the scope of this paper. Please note that the focus of our study is on the computational analysis of protein expression patterns and their use to predict protein function. Studies like ours typically provide experimental validation for only one or two candidates, whereas we systematically tested 20 phenotypes for 20 candidates. Reviewer #1 agrees that this is already an "impressive" experimental validation effort.

A	High progulonFinder score (candidates)	Low progulonFinder core (control)
High-scoring in siRNA screen (> ½ max)	10	1
Low-scoring in siRNA screen (< ½ max)	10	4

→ *p-value = 0.2449*

Notes: Distribution as shown in Fig. 3f (without positive controls) and as used by reviewer 2.

B	High progulonFinder score (candidates)	Low progulonFinder core (control)
High-scoring in siRNA screen (> ½ max)	100	10
Low-scoring in siRNA screen (< ½ max)	100	40

→ *p-value = 8.01e-05*

Notes: Same as (A) but multiplied by 10 to show that the calculation in A is underpowered

C	High progulonFinder score (candidates)	Low progulonFinder core (control)
High-scoring in siRNA screen (> ½ max)	12	3
Low-scoring in siRNA screen (< ½ max)	10	21

→ *p-value = 0.002841*

Same as (A), but including 2 green positive control proteins (added to top left) and 19 orange "negative control" proteins (2 top right, 17 bottom right) from Extended Data Fig 4.

Reviewer figure R3: 2x2 tables and notes underpinning the p-value calculations. These calculations were performed in R using a one-sided Fisher's Exact test.

Another way of overcoming the power issue, as this reviewer points out in the next question, would have been to include more low-scoring proteins in the follow up. We have not done this due to the effort and costs involved in validating more than 5 negative controls. However, the 19 additional candidates that we subjected to the siRNA screen as part of Extended Data Fig. 4 could be used as alternative “negative controls”. These proteins scored below cut-off in the default ProteomeHD-wide replisome progulon prediction. They were only subjected to the siRNA screen because they scored high in a special set of predictions based on 15 proteomics experiments involving the nascent-chromatin-capture (NCC) technique. As shown in Extended Data Fig. 4, only two of these proteins (GLRX5, NUDT9) scored high enough in the siRNA screen to be included in the “high-confidence” set. In addition, we could also include the two positive control proteins (ASF1A-B and CDC45) for the significance test. These scored high both in the progulonFinder prediction and the siRNA screen. Once we take all our data into account, we do obtain a very significant p-value of 0.0028 (**Reviewer figure R3C**).

> The authors argue that the "low RF score" hit in the high confidence set (LGALS3) is actually involved in DNA replication based on a previous publication. While this is true, it still means that low and high RF score proteins do not behave significantly differently in the genetic screen.
>
> Importantly, even if LGALS3 would be removed from the data, the hypergeometric test would not show a significant enrichment of "new candidates" in the "high confidence" subset. The main problem of the screen seems to be that too few "low RF score" genes were included. As it stands, the data does not support the statement that progulonFinder can identify new proteins involved in DNA replication.

As outlined in the previous answer, even without excluding LGALS3 from the calculation, the distinction between low and high RF score proteins is significant once we consider all our data. However, an additional reason for why the hypergeometric test above cannot be applied here is that a low progulonFinder score does not imply that a protein has no replication function. Lack of co-regulation does not mean lack of co-function, since the regulatory pattern of a protein can be a mixture of multiple different functions. The LGALS3 protein is actually a good example for this: it is a multifunctional protein which is also active in the cytoplasm and as a secreted protein. This would explain why progulonFinder did not detect it as co-regulated with the replisome. We have updated the text to clarify this.

> The validation of predictions could be much improved if they investigated how these candidates perform in other, more comprehensive, genome-wide, cell cycle/DNA damage related screens assessed either flow cytometrically (https://doi.org/10.1016/j.molcel.2009.06.021) or microscopically (MitoCheck - https://www.mitocheck.org/screens_browser.shtml). Functional validation of further progulon candidate members in other pathways (e.g. secretion or DNA damage - see MitoCheck or other resources) would further strengthen the case.

We provide three layers of orthogonal, external validation of our predictions. First, we manually annotate three progulons based on existing literature (one of which, the ATP synthase progulon, in high detail). Second, for a more systematic approach, we test each

progulon for GO term enrichment. In the revised version of the manuscript we now added two additional dimensions, showing that progulons are also enriched for Reactome pathways and protein complexes. The third layer is the siRNA based validation of the replisome progulon. Note that the MitoCheck resource focuses on mitosis rather than S-phase. We now also cross-referenced the progulonFinder results for our replisome prediction with the first of the resources mentioned here by the reviewer. That study by Paulsen *et al* (Mol Cell, 2009) is a genome-wide siRNA screen monitoring γH2AX phosphorylation. Less than half of our 20 new candidates were covered by that screen and none of them had significantly elevated γH2AX phosphorylation levels. Please note that we also measured γH2AX phosphorylation levels in response to siRNA-mediated knock-down of these proteins and only in one case, ATAD2, did we find an increase in γH2AX phosphorylation upon siRNA-mediated depletion. However, for many candidates we detect a reduction in γH2AX phosphorylation upon challenging siRNA treated cells with drugs such as hydroxyurea and aphidicolin (Appendix Fig S8). This shows that our approach, combining co-regulation based prediction with monitoring multiple siRNA phenotypes, captures a broader spectrum of phenotypes than screening for γH2AX phosphorylation alone, suggesting that this could be the reason why a replication-associated functions of these proteins were not detected earlier. Finally, note that overall we do find an enrichment of proteins with significant γH2AX phosphorylation phenotypes according to Paulsen et al among the proteins in the replisome progulon (**Reviewer Figure R4**).

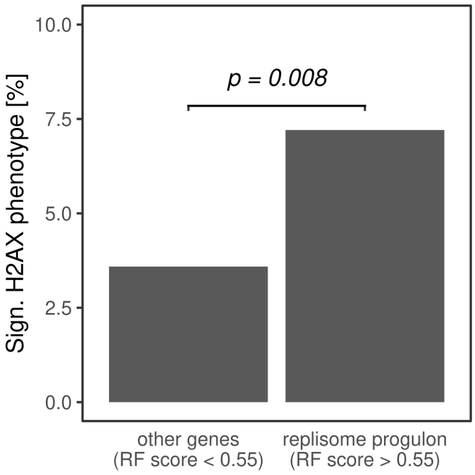

Reviewer figure R4: Members of the replisome progulon are more likely to have a significant H2AX phosphorylation phenotype in the screen by Paulsen *et al*. This plot only considers the most significant hits by Paulsen *et al* (referred to as "level 4" hits in their paper).

> Line 372: "We demonstrate that even for a well-studied process such as DNA replication, new factors can be identified with high accuracy (~75% validation rate)." As stated above this alleged high accuracy is actually not statistically significant.

Please see our responses above for why we believe this statement is, in principle, justified. However, we have softened this statement by rephrasing it as: *We demonstrate that even for a well-studied process such as DNA replication, new factors can be identified with high confidence.*

> Line 374": "While our work was in progress, replication-related functions for three of our predictions were independently confirmed: ATAD2 , FTO and ZMYM3." The corresponding publications date back to 2016 and 2017. Therefore, the argument that these were independently confirmed while the "work was in progress" is somewhat weak.

We agree with this statement. We now simply state in the manuscript that these three proteins have known replication-related functions.

> Progulon-specific explorations regarding perturbations: The authors clearly explore the fact that the nature of the perturbations encompassed by the ProteomeHD database is not as important as their sheer number. While this may be true, this reviewer feels that the functional impact and novelty of the study could be improved by exploring the perturbations whereby co-regulation of a progulon becomes weaker. For instance, in Figure 1C to E (lower panels), the 'seed' groups of proteins tend to follow very closely to the novel interacting candidates of the progulons. Nonetheless, there are clearly perturbations which lead to a breakdown in this relationship (i.e. when the magenta and blue lines part from one another). Can any conclusions be drawn from focusing on these specific conditions? For instance, do some perturbation specifically decouple potential post-transcriptional regulations between different parts of the progulon? Can the authors elaborate on potential mechanisms behind this?

We thank the reviewer for this suggestion, which led to an additional figure described in response to the next question below. Note that lack of co-regulation in a particular subset of samples can have both biological and technical reasons. Biologically this could be the result of a protein being multifunctional, with different functions dominating the regulatory pattern in different samples. Technical reasons could include identification or quantitation errors associated with a particular sample.

> Along the same lines as comment 4), it would be informative to have a heatmap displaying the coordination of each progulon as a function of perturbation (as in Romanov et al. 2019 Cell https://doi.org/10.1016/j.cell.2019.03.015 Figure 3) either in the supplement or in the main figures.

We have taken this comment on board in the new Figure 2H, which is based on the figure by Romanov and colleagues. Our new figure shows the mean Spearman's correlation of different progulons, calculated either across the entire ProteomeHD dataset or across sub-sections of ProteomeHD. These sub-sections are projects composed of more than 10 experiments, for example lymphoblastoid cell lines from different human individuals or endothelial cells grown in different adhesion conditions. The figure shows that there can be substantial variation in the extent of module co-regulation across different sub-projects.

> Minor comments and suggestions
> The authors should revise their figure annotations (e.g. there is no annotated Supplementary Figure 2C)

We have corrected this mistake now.

> The relationship between protein variation of a progulon and its respective protein-mRNA PCC seems a bit circular: it is an exercise in comparing the covariance of two variables to the variation in one of those variables. In any case, it would be

interesting for the authors to always outline with colours which of these progulons correspond to mitochondrial and cytoplasmic ribosomes, given that these seem to always be the outliers (in Supplementary Figures 2, 3 and 4)

We now highlight mitochondrial and cytoplasmic ribosomes in colour in Appendix Figures S4, S5, and S6 (previously called Supplementary Figures 2b, 3b, 4b).

In figure 1h, several of the progulons seem to have overlapping members with one another ('off diagonal' clusters). The authors could perhaps elaborate on these cross-pathway cameos and whether or not they are expected based on the literature.

This is now Appendix Figure S3A. In the revised version of the manuscript there are fewer progulons with less redundancy between seed groups due to the improved clustering and filtering steps. Consequently, there is also less overlap between the final progulons. However, significant overlap persists between four mitochondrial progulons (especially the two seeded by different sub-groups of electron transport chain complex I) and also between progulons related to mRNA processing. We have included a sentence in the text to reflect that.

The authors underline how the average half-lives of mRNA and proteins seem to correlate for progulons, with entities such as the proteosome displaying high half-lives. How about the coordination of mRNA and protein half-lives within progulons? Do these follow the same trends as steady state abundances?

We thank the reviewer for this suggestion. There is indeed a significant correlation between the coordination of mRNA level changes of progulons and the coordination of the mRNA half-lives. The ribosome progulon is an exception to this. Interestingly, no such correlation exists at the protein level. We have updated Extended Data Figure 3 to include two new figure panels that demonstrate this and have added a statement referring to them in the main text.

Supplementary Figure 2 b: The axis label and legend is uses the term "median MAD", which means "median median absolute deviation", so "median" is redundant.

This is actually referring to the “median MAD”, i.e. the median of the “median absolute deviations” of proteins (or mRNAs) in each progulon. MAD is a robust alternative to the standard deviation, so this is the equivalent of talking about the “median SD” of the proteins in a progulon. We have rephrased the corresponding figure legends to clarify this.

Reviewer #3

The authors of the manuscript used human proteome data across 294 perturbations to train a model for the prediction of protein co-expression clusters. The output of their model is a set of 41 protein regulons ('progulons') that are significantly co-regulated in terms of their protein abundances.

The authors then use transcriptomic and proteomic data from cancer cell lines to try and determine whether the 41 progulons are co-ordinated at the level of mRNA expression (transcription and degradation) or at the protein level (synthesis and degradation). They later divide their progulon proteins into exponentially degraded (ED) and non-exponentially degraded (NED) proteins to try and account for the patterns they observe.

The authors have made their random forest (RF) classifier available in the form of a webtool (progulonFinder). They use this to predict new components of the replisome as a case study. For validation, RNAi screens for replication-linked phenotypes were then performed on 20 new replisome candidates.

Main conclusions and concepts:

• Identification of 41 protein co-regulation clusters in the human proteome, covering ~4000 proteins. The prediction tool has been made publicly available for re-use.
• Progulon proteins tend to have a lower mRNA-to-protein correlation than the proteome average, indicating a role for regulation after transcription.
• Finding of no positive relationship overall between mRNA co-expression (co-ordination) within progulons and the mRNA-to-protein correlation (contribution).
• Progulons are enriched for non-exponentially degraded proteins (NEDs), which are often regulated by proteasome-mediated degradation. This may explain the discord between co-ordination and contribution.
• Prediction of new replisome components as a proof-of-principle, with an attempted validation using RNAi-based screens of replication-linked phenotypes for 20 candidates.

We would like to thank the reviewer for this accurate summary and principally positive assessment of our manuscript.

General comments:

This manuscript follows on from a paper published recently in Nature Biotechnology (Kustatscher et al., 2019). Relative to that paper, the authors try to establish novelty here by using a supervised learning approach to generate co-regulated clusters instead of an unsupervised or correlation-based approach. However, they do not demonstrate for this dataset (proteomeHD) the effectiveness of this method over and above unsupervised or correlation-based approaches.

We have now expanded our analysis and tested three different unsupervised clustering approaches (hierarchical, density-based and graph-based) as well as a correlation-based approach suggested by the reviewer. See the respective comments below for more details.

In the second part of the manuscript, the authors use transcriptomic and proteomic data to probe the mechanism (transcriptional vs. post-transcriptional/post-translational) underlying protein co-regulation. The enrichment of progulon proteins for non-exponentially degraded proteins (NEDs) is used to help explain the lack of an expected correlation between co-ordination and

contribution (Figure 2h). While potentially interesting, I do not find their conclusions wholly convincing given problems with the underlying data and statistical analysis.

We agree with reviewers #1 and #3 that the evidence presented regarding NEDs is still under-developed. Therefore, we have taken the decision to remove the reference to NED as explanation for the lack of mRNA - protein correlation from the results section, together with the original Figures 2h and S5. However, we allude to this possibility in a new paragraph of the discussion section.

Their RNAi-based validation screen for new replisome components provides reasonable support for the results of progulonFinder

We thank the reviewer for this assessment and would like to point out that we also include substantial computational validation of our results, cross-referencing progulons with the literature and with gene function data from GO, Reactome and huMAP.

Overall, this research has the potential to reveal some useful insights into protein co-regulation and the mRNA-to-protein relationship. However, the novelty of this research and the extent to which the data support the conclusions are questionable. The NED-associated analysis in particular (Figure 2H) is unconvincing.

As noted above, we have removed the NED associated analysis from the manuscript. We also note that there is a substantial difference between this manuscript and our earlier NBT paper. In the latter, we only analysed protein expression data to identify pairwise protein - protein associations, in an entirely unsupervised manner. Here, we identify large co-regulation modules in the human proteome, which could only be captured by a supervised machine-learning approach.

Major points:

• The authors emphasise in the manuscript that progulon control is predominantly 'post-transcriptional'. However, this term refers to all steps between transcription and translation and not simply all events after transcription. The proteasome-mediated degradation of proteins is a post-translational mechanism, for example. This confusion should be corrected throughout the manuscript.

We have corrected this through-out the text, changing most instances to either refer to mRNA and protein level regulation, or to specify both post-transcriptional and post-translational regulation separately.

• The authors use a supervised learning approach to identify progulons from the seed proteins in clusters. In principle, similar results could be obtained by iteratively retrieving highly correlated/co-regulated protein pairs for each protein in the seed set and then taking the union. This could be achieved using the existing proteomeHD resource and using a correlation/co-regulation threshold producing similar sample sizes

> to the progulons from progulonFinder. This analysis should be performed and the results benchmarked alongside those of progulonFinder for functional relevance (using GO enrichments and Reactome, for example). This is necessary to establish the novelty/effectiveness of progulonFinder relative to the Kustatscher et al., 2019 paper.

Our approach actually combines unsupervised and supervised approaches. First, an unsupervised clustering algorithm (OPTICS in the original version of the manuscript) is used to identify tightly co-regulated “seed groups”. These are then used as training set for a supervised learning approach. The reason we do not simply use unsupervised ML / clustering to explore modules in ProteomeHD is that, in our hands, these approaches only succeed in finding relatively small, tightly correlated clusters. In contrast, the supervised approach essentially constructs a model of which patterns of up- and downregulation are relevant for a seed group, and then tests all other proteins to see if they (partially) match that. In this way we find much larger co-regulation modules, which still contain functionally closely related proteins.

As a control, we have implemented the intersection strategy suggested by the reviewer. In short, we calculated pairwise Pearson correlation (PCC) for ProteomeHD and filtered the correlation matrix specifically for seed proteins of one exemplary progulon (ATP Synthase). We filtered for pairwise correlations above a given PCC cutoff for each seed protein and then intersected each of these lists consecutively. By testing different PCC cutoffs we found that at PCC = 0.14 this strategy produced an ATP synthase module of similar size as the equivalent one obtained by progulonFinder. We finally performed a GO overrepresentation analysis for both the PCC picked module and the ATP Synthase progulon. Although both modules

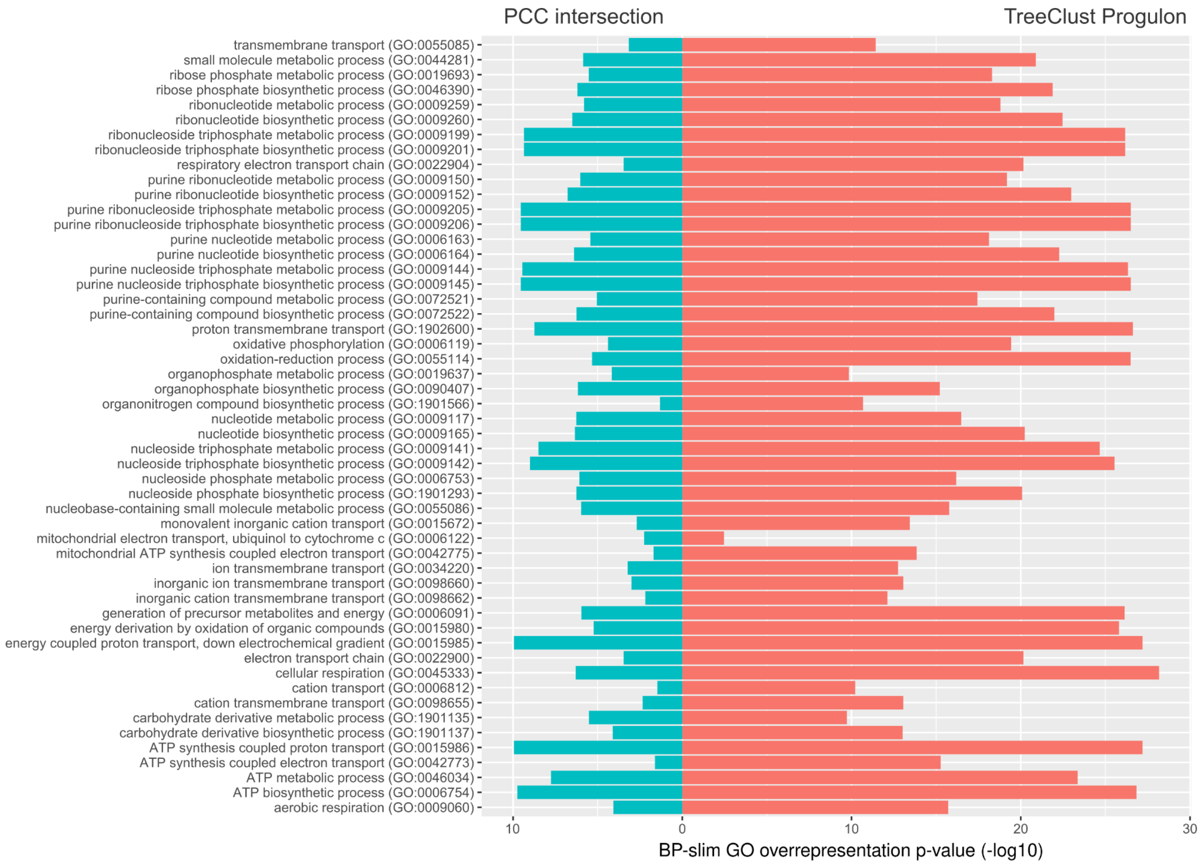

Reviewer Figure R5: GO overrepresentation in ATP synthase module found by PCC intersection (left) and progulonFinder (right)

calculated in this manner are significantly overrepresented in many GO terms, this enrichment tends to be much weaker for the PCC intersection strategy (**Reviewer Figure R5**).

However, this intersection strategy is reminiscent of a graph-clustering approach employed by the clusterONE algorithm ("Clustering with Overlapping Neighborhood Expansion") (Nepusz, Yu and Paccanaro, Nat Methods, 2012). To further increase the confidence in our seed groups, we now use both OPTICS and clusterONE to calculate two sets of seed groups and use the intersection of these two groups as training data for progulonFinder.

> BULLETPOINTS
> • Figure 1g shows that some progulons have an overlap as high as 80%. It is not surprising therefore that such progulons will have highly correlated expression profiles if they share components. Figure 1g seems to mirror figure 1h for this reason. The average (mean or median?) correlation should therefore be recalculated after excluding self-self (r=1) correlations. The statement 'Expression changes of functionally similar progulons are correlated (Fig. 1h)' is not currently supported by the data for this reason.

We have re-calculated the correlation between the progulons without considering overlapping proteins. Although this changes some of the correlation values, the overall conclusion that expression changes of functionally similar progulons are correlated remains unaffected.

> • For the GO enrichment analysis in Fig. 1I, GO enrichments should be performed for completely random progulons of the same size to determine if summing the number of GO terms observed within each progulon is meaningful. The results can be included as a supplementary figure.

We have now tested what happens if we attempt to calculate progulons from completely random seed groups. We found that this does not result in any progulons at all, since such random progulons do not pass our quality filters. In addition, we now tested how likely it is that a random group of proteins with the same size distribution as our progulons is enriched in GO terms. This has been added to Appendix Figure S3.

> • Seemingly arbitrary decisions are often made when it comes to the statistical analysis. For example, Spearman's correlation is used in Fig. 1H but Pearsons's for Fig. 2A-C. Also, correlation coefficients are calculated in Figs. 2 D-E but not in Fig 2C? Inconsistencies like this should be justified.

We generally used Spearman's correlations where there are outlier points, and Pearson's correlation where there are not. However, for consistency we now simply use Spearman's correlation coefficients throughout the manuscript. The dotted line in the previous Figure 2C was not a linear fit but simply a diagonal line dividing the data in progulons that are better coordinated on mRNA and protein level, respectively. This is why no correlation coefficient was given in that figure. We have added a regression line now (see new Figure 2C).

- Much of the analysis for Figure 2 has been repeated for two additional datasets (Supplementary Figure 3 and Supplementary Figure 4). The claim that the differences between results is 'minor' is highly dubious. The median mRNA-protein correlation in Supplementary Figure 3 for example is only 0.15. There also appears to be no correlation in the figure between mRNA coexpression and protein coexpression. The enrichment of progulons for low mRNA-to-protein correlations (implying regulation after transcription) also seems to disappear in this figure. Also, the negative correlations found in Fig 2D and Fig 2E seem to disappear in supplementary figures 3 and 4. These are significant deviations that undermine some of the conclusions in the main text.

The figures repeating the mRNA - protein comparison for lymphoblastoid cell lines (LCL) and mouse tissues are now Appendix Fig S5 and S6, respectively. They have been re-produced since the underlying progulon data changed, but are otherwise still very similar to the figures from the first manuscript version. It is correct that these two datasets show overall a poorer correlation between mRNA and protein levels than the breast cancer dataset, and we now state this in the text. We also no longer claim that the differences are "minor".

However, we are not sure what the subsequent statements of the reviewer refer to, since we actually find that mRNA coexpression and protein co-expression are strongly correlated also in the LCL (Fig S5C, rho = 0.7, p < 1.8e-5) and mouse tissue dataset (Fig S6C, rho = 0.74, p < 2.3e-6). Similarly, in both cases we still find that the majority of progulons are enriched for low mRNA-to-protein correlations, implying post-transcriptional / post-translational regulation. To avoid possible confusion, we have now divided these figures into sub-panels and refer to them explicitly in the main text.

Finally, it is also correct that the negative correlations found in Fig 2D,E disappear in the LCL and mouse tissue dataset. Note that we referred to this as "lack of correlation" in the text.

Our main conclusions from Figure 2 (breast cancer dataset) were that mRNA and protein coexpression are correlated, that most progulons appear to have poor mRNA - to - protein correlation and that there is no (positive) correlation between mRNA "coordination" and "contribution". These points have been clearly confirmed by the two additional datasets, which is why we had referred to the differences as "minor".

- The authors should also state in the main text that the results for supplementary figure 4 derive from one-to-one ortholog mappings between human and mouse for each progulon, and link this to the observation in Figure 2.I that only ~30% of co-expressed human proteins within a progulon are also co-expressed in mouse. This brings into question the appropriateness of using this data set as a means to validate the Figure 2 results.

Figure 2I looks at protein pairs that are strongly co-regulated in both human and mouse (rho > 0.5). As we now switched from Pearson to Spearman's correlation, which is less sensitive to outliers, the proportion of conserved protein pairs increased to 34%. When considering protein pairs that are at least weakly correlated (rho > 0.25) in both human and mouse, this proportion jumps to 47%. Moreover, Supplementary Fig. 4 (now Appendix Fig S6) focusses on progulons, and there are many pairs within these modules that are correlated with lower coefficients. Moreover, consider that a progulon with 100 proteins gives rise to 4,950 pairwise associations, therefore if even half of these are evolutionarily conserved (one-to-one

orthologs), then we still have sufficient data for appropriate statistics. This is also reflected in the fact that the mouse data do agree well with the other test sets.

- For all of the correlations given in Figure 2H, the regression lines seem to be heavily influenced by the two outliers (orange dots). Little-to-no correlation is apparent by eye for the remaining data points. The analysis has been repeated without the outliers in supplementary figure 5c, but the data for breast cancer cells has not been plotted (as in Supplementary figure 5b). This data should be plotted explicitly and r or R2 values displayed in the plots. The relevance of [NED%] as an explanatory variable should then be re-evaluated in the main text, given the weak r/R2 values calculated after outlier exclusiom.

Figures 2h and S5 were intended to show that NED could explain at least partially why there is a poor correlation between mRNA and protein expression changes. However, as explained above, due to this relationship being too tentative at the moment we have removed these plots from the manuscript.

- The claim is also made that the trend observed in Figure 2H (bottom-left) persists even after the exclusion of outliers. However, this is only the case for the mouse tissues and not the LCL cells. In fact, 3/6 of the correlations tested in Supplementary Figure 5b become non-significant after the outliers are excluded. This should be stated in the main text, and again undermines some of the conclusions in the main text. The conclusions given should be re-evaluated in light of these results.

These figures and statements have been removed now. We had included Fig. S5 for exactly this reason, i.e. to show the reader that there is evidence for NEDs playing a role in these relationships, but that this evidence is relatively weak and partially driven by two specific progulons. For the record, 4 of 6 relationships in S5b were significant with the outliers, and one of these lost significance without them.

- The authors identify ~4,000 proteins that belong to progulons. This leaves ~15,000 proteins that cannot be assigned to co-regulatory modules. The authors should comment in the discussion whether they believe this represents a biological reality or can be accounted for by data/statistical limitations. The stringent AUC threshold (AUC>0.99) adopted for progulon filtering may be relevant here.

The question of how many progulons there are exactly, and how many proteins belong to any progulon, is difficult to answer. We have addressed this by including the following statement in the discussion section: *While we report here on the discovery of progulons, it is difficult if not impossible to state their exact number or protein composition. Very much like the definition of protein families based on multiple sequence alignments, better algorithms and more/better data will likely increase the number of assigned progulons and affect their precise composition.*

Minor points:

• For figure 1 C-E, please explain in the legend your criteria for selecting the 25 different experiments shown in the line plot. Were they selected at random?

These 25 experiments were not selected randomly, but they were selected systematically in order to illustrate the behaviour of progulon proteins. They were selected using a genetic algorithm (GA), which is a type of optimisation algorithm. It was instructed to identify 25 "useful" experiments, defined as having actually detected the proteins in question and showing some (even modest) expression changes of those proteins. These are the type of experiments a Random Forest algorithm would use to decide into which category a protein falls. We have updated the figure legend to say that these are "representative" experiments.

• It should be explained somewhere exactly how the percentage overlaps in Fig. 1G were calculated. The authors should also explain how missing data was handled when calculating the Spearman's correlation in Fig. 1H.

Fig. 1G is now Appendix Fig S3 and it now shows the overlap both in protein numbers and in percent. The percentage overlap is given relative to the bigger of the two progulons in each pair. This is now indicated in the figure legend. Only pairwise complete observations were used for correlation analysis, i.e. missing values were ignored. This is now described in the Methods section.

• Line 152→ 'In contrast to bacterial and eukaryotic operons, we find no substantial clustering of progulon gene *in terms of chromosome location*'.

We have added the part "in terms of chromosome location" to this sentence.

• On line 174, indicate the significance threshold used for the test.

The significance threshold was $p = 9.9e^{-05}$. This has been added to the text.

• The negative correlation found for Figure 2D lies above the standard significance threshold ($p = 0.064 > 0.05$) and seems to be determined strongly by the 'Ribosome' data point. This should be stated in the main text.

We have updated this part of the text and analysis. With the optimised set of progulons this trend is now significant and persists even if the ribosome is excluded.

• The manuscript refers to a 'Supplementary Figure 2C.', but this does not seem to exist.

Thank you, this has now been corrected.

• In relation to Figure 2H (top-left) please state the number of progulons that lie significantly above and below the 10% threshold.

This figure panel has been removed from the revised version of the manuscript.

- The presentation of the phenotype data for Figure 3 is confusing. I think a brief description of the Figure 3E phenotypes should be given in the legend and also in the legend of supplementary figure 7 for all phenotypes listed to aid non-specialists. It is also not clear to me why 20 phenotypes are tested and yet the maximum siRNA score given is 13 and not 20? The green/yellow/purple color scheme used in Figure 3e and figure 8 also does not seem to correspond to the color scheme used in supplementary figure 7 as the colors represent different concepts. This is very confusing. In general, figure legends for supplementary figure 7 and supplementary figure 8 should be more detailed. It is also not clear if the results in figure 3f derive from the RSA- or SD-based scoring metric.

We have now added a description of the phenotypes to the legends of figure 3 and 7 (the latter is now S8). In addition, we changed the colour scheme used in Figs 3, EV4 and 8 (now S9) to avoid any confusion with the phenotypes of figure 7 (S8). We have also expanded the description of the screen scoring in the methods section (i.e. the reason why max score is 13 and not 20), and expanded the figure legends of 7 (S8) and 8 (S9) accordingly. Finally, we have clarified that the scoring metric used for the main figures was the SD-based one.

- For all figures and supplementary figures where a correlation has been calculated, state whether this is a Spearman's or Pearson's correlation coefficient if this has not already been shown in the figure.

We now use exclusively Spearman’s rank correlation in this manuscript and represent this in all figures with the letters rho.

Thank you for sending us your revised manuscript. We have now heard back from the three reviewers who were asked to evaluate your revised study. As you will see below, reviewers #2 and #3 are satisfied with the performed revisions and are supportive of publication. However, reviewer #1 lists some remaining concerns, which we would ask you to address in a final round of revision. Let me know if there is anything you would like to discuss in further detail related to the reviewer's comments.

We would also ask you to address some editorial issues listed below.

Reviewer #1:

The authors revised their previous work entitled "Higher-order modular regulation of the human proteome." However, there are major issues remaining, most related to the usage of https://www.proteomehd.net/progulonFinder.

1. We still cannot appreciate the logic of presenting "progulons". In the abstract, the authors suggest, "Progulons consist of dozens to hundreds of proteins that together mediate core cellular functions". The discovery of these "31" progulons is based on the entire proteomehd data basis. However, the webtool named "progulonFinder" only asks for 5-50 proteins (online version) as training proteins which seem report "additional proteins" showing similar up- or down- regulations compared to the training proteins, as a "progulon". As we commented during the first review, the "progulon" is not a stable concept and is depending on the input lists. It might make more sense if the authors present the webtool first and then clarify that the idea is to find high-order regulations on small lists of proteins. Then, the "31" progulons can be used as an example of applying this webtool - the clustering was only used to detect "seeds" for generating examples to demonstrate the tool. Accordingly, how a progulon is called (e.g., only 31 of the 72 seed modules produced a progulon) needs to be fully described and rationalized in the main text. In any case, we feel like the logic flow and the discussion about the concept of progulons need to be improved.

2. The performance of progulonFinder. The webtool provided https://www.proteomehd.net/progulonFinder asks the user to provide an email address. Please note that this is against the anonymity of the reviewer process, precluding us from testing its performance and usage. Please remove the requirement of email, at least during the peer-review process.

3. A related question is about the requirement of the input "seed". Currently, it allows as low as five proteins. How do they ensure the stability of the output list and avoid overfitting using such a small list? Additionally, could the author show some examples of very small lists?

4. Figure 2. The author used the example dataset of breast cancer cell lines to demonstrate that "as the coordination of mRNA or protein expression changes becomes stronger, the contribution of mRNA to protein changes becomes weaker" and claimed that the removal of "ribosome" and "proteasome" still generate negative correlation (Page 8, 1st paragraph). However, very clearly, in the other two example datasets (Appendix Figure 5HI and Appendix Figure 6HI), removing two or three items will not generate a negative correlation. The author should to tune down the claim. Also, it seems the negative or no correlation between protein/mRNA coexpression and mRNA~protein correlation will be not only dependent on "progulons" and can also be true for e.g., protein complexes. The author should include similar analysis for protein complexes.

Reviewer #2:

I thank the authors for addressing most of the points I raised. I still think that (i) the overall conceptual novelty is somewhat modest and that (ii) the paper would greatly benefit from more extensive experimental follow-up. However, I also see that the paper makes a significant contribution to the field and therefore think it can be accepted for publication.

Reviewer #3:

I would like to thank the authors for taking the time to write a full response to the review. All of my specific points were appropriately addressed, and I now consider all of the major claims in the manuscript to be adequately supported by the underlying data.

In reference to the comments I made in the original review about the (then) Supplementary Figures 3 and 4 (first bullet point of page 19/22 in the response to reviewers), I would like to clarify my original comments. First I would like to apologise for my

ambiguous and unclear reference to particular panels in these supplementary figures. For the following statement:

'There also appears to be no correlation in the figure between mRNA coexpression and protein coexpression.'

I was referring to the original Supplementary Figure 3 panel C (second sub-panel from the left), which is now Appendix Figure S5G. The original panel gave no correlation coefficient and by eye the correlation looked much weaker than in the original Figure 2C. However, with the updated analysis this correlation is clearly significant (rho = 0.7, $p < 1.8e-5$) and is now given in the figure panel. For the following statement:

'The enrichment of progulons for low mRNA-to-protein correlations (implying regulation after transcription) also seems to disappear in this figure'

I am unable to recollect which panel I was referring to as the original review was so long ago. There may have been a mistake in the wording of the original sentence. However, after reading the response of the authors and reviewing the relevant figures I am satisfied that the main text faithfully represents the data.

Overall, I think this manuscript represents a significant advance albeit a fairly modest one in the context of Kustatscher et al., 2019 (Nature Biotech). However, I appreciate the statistical and methodological rigour that has been offered for the revisions and the easy accessibility of Progulon Finder to non-computational biologists: https://www.proteomehd.net/index

Response to the reviewers, Kustatscher et al (MSB-20-9503R)

We would like to thank all reviewers for their feedback on the revised version of our manuscript. We provide a point-by-point response to the remaining comments and editorial suggestions below.

Response to reviewer #1

> The authors revised their previous work entitled "Higher-order modular regulation of the human proteome." However, there are major issues remaining, most related to the usage of https://www.proteomehd.net/progulonFinder.
>
> 1. We still cannot appreciate the logic of presenting "progulons". In the abstract, the authors suggest, "Progulons consist of dozens to hundreds of proteins that together mediate core cellular functions". The discovery of these "31" progulons is based on the entire proteomehd data basis. However, the webtool named "progulonFinder" only asks for 5-50 proteins (online version) as training proteins which seem report "additional proteins" showing similar up- or down- regulations compared to the training proteins, as a "progulon". As we commented during the first review, the "progulon" is not a stable concept and is depending on the input lists. It might make more sense if the authors present the webtool first and then clarify that the idea is to find high-order regulations on small lists of proteins. Then, the "31" progulons can be used as an example of applying this webtool - the clustering was only used to detect "seeds" for generating examples to demonstrate the tool. Accordingly, how a progulon is called (e.g., only 31 of the 72 seed modules produced a progulon) needs to be fully described and rationalized in the main text. In any case, we feel like the logic flow and the discussion about the concept of progulons need to be improved.

We thank the reviewer for these suggestions. We agree that inverting the overall layout of the manuscript (webtool first, progulons later) would also be a viable “narrative”. However, such a massive change would require a complete rewrite of the manuscript and, more importantly, would also shift the focus of the manuscript from the existence of higher-order protein co-regulation modules to the technology developed to detect them. Nonetheless, we appreciate the suggestion by the reviewer that the concept of progulons, in particular their “stability”, is still insufficiently described and have therefore expanded the discussion section accordingly. It now reads as follows:

While we report here on the discovery of progulons, it is difficult if not impossible to state their exact number or protein composition. There are both biological and technical reasons for this. Different cell types, for example, contain a different set of proteins and require a different set of functionalities. It is therefore expected that their protein co-regulation modules may be somewhat different, too. The exact composition of a progulon will also be somewhat dependent on a range of technical factors, such as the choice of algorithms and parameter settings, input data, and seed lists. In this sense, our current view of progulons is reminiscent of early definitions of protein families based on multiple

sequence alignments: on one hand, it is likely that better algorithms and more/better data will in the future contribute to the discovery of more progulons and affect their precise composition. On the other hand, the existence of such modules, and the ability to detect them using an online tool, may provide a new entry point for the functional characterisation of understudied human proteins.

Regarding the description of how progulons are called, i.e. how we arrive from 72 seed proteins to the 31 "final" progulons, we describe this in detail in a 460-word section under Material and Methods ("Identifying progulons from seed proteins"). We have now added a shorted version of this to the main text, as requested by the reviewer. The corresponding main text paragraph now reads as follows:

We designed several stringent filtering criteria to ensure the high quality of the final list of progulons. This included a requirement for the cross-validated training data to achieve a ROC curve area above 0.99 and a requirement that at least 4 of the 10 proteins with the highest RF scores had to be cross-validated training proteins. Finally, we introduced a requirement for progulons to be genuine co-regulation modules, i.e. a group of proteins that are not only co-regulated with the seed proteins but also with each other. This was achieved by calculating a "connectivity score": we compare progulons to a co-regulation network created with all proteins in the analysis and, using a Fisher's Exact test, calculate a p-value that reflects if a progulon is enriched for protein pairs that are among the top 0.5% of co-regulated protein pairs in the overall network. We then chose the minimum RF score cut-off that creates a significantly inter-connected module (see Methods). Only 31 of the 72 seed modules produced a progulon which passed all of our quality control filters (Dataset EV2).

> 2. The performance of progulonFinder. The webtool provided https://www.proteomehd.net/progulonFinder asks the user to provide an email address. Please note that this is against the anonymity of the reviewer process, precluding us from testing its performance and usage. Please remove the requirement of email, at least during the peer-review process.

We apologise for this inconvenience. We understand that the email feature makes it harder to use the online progulonFinder service anonymously. However, we cannot disable this feature, because it is an integral part of the service, as the search results are transmitted to the user via email. This design choice was made because, depending on the server load, it can take between 10 minutes and several hours for the results to be ready. In this context, we believe it makes more sense to deliver the results by email whenever they are ready, rather than expecting a user to keep a browser window open and repeatedly refresh it to see if the search is finished. Please note, we do not store email or IP addresses or any other users details, we just need the email address to return the results.

One option for the reviewer would be to use a "disposable email" service such as https://www.emailondeck.com, which provides users with a temporary email address and inbox.

This can be created within seconds, without login details, and in our tests such addresses could be used successfully to receive progulonFinder results.

In addition, to allow the reviewer to inspect a typical result of our online tool, we append to this document the two files that would be created for a search with 13 subunits of the ATP synthase complex (Reviewer Appendix 1). The first is a table in .csv format (suitable for both Excel and R), which contains the list of all proteins (we only show the first page in the appendix below) together with their Random Forest scores and multiple annotation columns, which are described in detail on the website to help a user interpret the output. The second file is a PDF report, which includes a methods paragraph, a plot, and statistical information about the Random Forest model, e.g. the number of training proteins used and the area under the ROC curve.

> 3. A related question is about the requirement of the input "seed". Currently, it allows as low as five proteins. How do they ensure the stability of the output list and avoid overfitting using such a small list? Additionally, could the author show some examples of very small lists?

The reviewer raises an excellent point. It is quite unusual for random forests to have such few training instances. However, as we have shown here and in Montaño-Gutierrez et al (Mol Biol Cell, 2017, PMID: 28057767), small training sets such as the subunits of a protein complex work surprisingly well for this type of challenge. Our progulonFinder automatically performs leave-one-out cross-validation of the training data and provides the user with a brief statistical report on this basis. For example, after searching progulonFinder with 5 random proteins (Reviewer Appendix 2), the report reads: "*Cross–validated training data were used to assess performance, yielding an area under the precision–recall curve (AUPRC) of 0.003. (A random classifier would yield an AUPRC of 0.005.)*". This makes it clear that these randomly selected training data did not succeed in identifying a group of co-regulated proteins. In addition, the report includes a scatter plot that visually reinforces the same message.

Random forests are generally considered to be among the most robust machine learning tools. However, if overfitting does occur, in our specific application the worst-case scenario is that the model may fail to identify "test proteins" that are co-regulated with the training data. We reasoned that a possible example for this could be training a model with the six subunits of the minichromosome maintenance (MCM) complex, which are known to be extremely tightly co-regulated. In this case we find indeed that the training data are strongly separated from all other proteins (Reviewer Appendix 3). Notably, even in that case, many of the top hits have replication-related functions.

Finally, the stability of the resulting progulons also depends on the chosen "negative class" training data. With a small training set this could, in principle, lead to substantial unwanted bias. However, we avoid this by running and averaging results across many different random forest models. For example, if a user selects 5 "seed proteins", progulonFinder will automatically and randomly select 5 negative training data, and create the first random forest model. It will then select another 5 negative training proteins and repeat the proteins 200

times (until a total of 1,000 negative training proteins have been sampled). The reported final scores are then the result of averaging the scores over all models.

> 4. Figure 2. The author used the example dataset of breast cancer cell lines to demonstrate that "as the coordination of mRNA or protein expression changes becomes stronger, the contribution of mRNA to protein changes becomes weaker" and claimed that the removal of "ribosome" and "proteasome" still generate negative correlation (Page 8, 1st paragraph). However, very clearly, in the other two example datasets (Appendix Figure 5HI and Appendix Figure 6HI), removing two or three items will not generate a negative correlation. The author should to tune down the claim.

We have added a sentence to make it clear that this observation is specifically referring to the breast cancer cell line dataset. The full sentence now reads as follows: *This trend is driven by the ribosome and proteasome progulons in particular, but persists in weakened form even if these two progulons are removed (rho -0.36 and -0.46), at least across this breast cancer cell line dataset.*

Please note that the key observation in this context is not that there is a negative correlation, but that there isn't a positive one. When talking about the data from Appendix Figs 5HI and 6HI we make this very clear in the main text: *In these datasets the negative correlation between coordination and contribution of expression changes is not significant. Nevertheless, this confirms that these are two separate aspects of gene regulation that are not directly, positively correlated as one might have expected.*

> Also, it seems the negative or no correlation between protein/mRNA coexpression and mRNA~protein correlation will be not only dependent on "progulons" and can also be true for e.g., protein complexes. The author should include similar analysis for protein complexes.

We thank the reviewer for this suggestion and have added three new figure panels showing that this is indeed the case. This new Appendix Figure S4 F-H shows that also for human protein

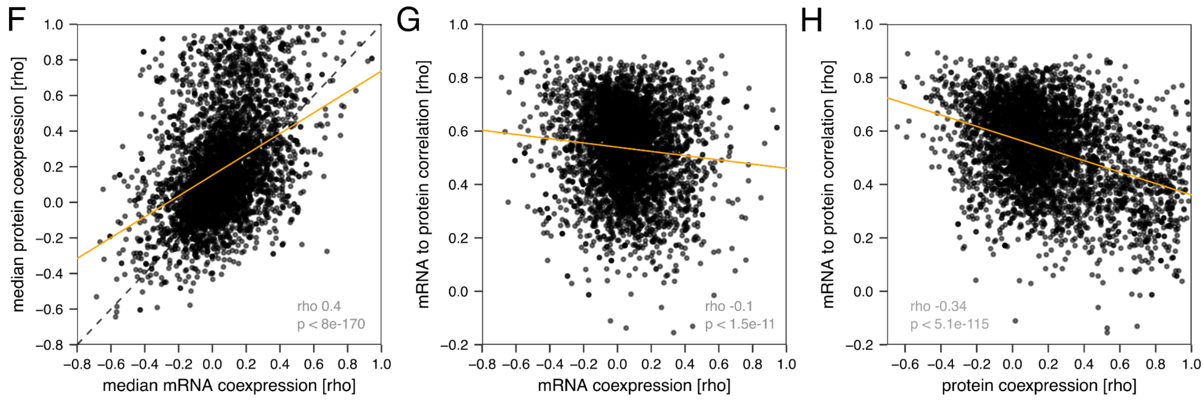

Appendix Figure S4 F-H: mRNA and protein abbundances across breast cancer cell line for Humap2 protein complexes.
(**F**) Protein coordination increases with the mRNA coordination (rho, orange regression line). The majority of Humap2 protein complexes are located on the upper side of the diagonal dashed line, suggesting that they are better coordinated on the protein level. (**G, H**) The mRNA-to-protein contribution is inversely correlated (rho, orange regression line) with both mRNA and protein coordination.

complexes of the hu.Map2 collection (Drew et al, MSB, 2021) there is a positive correlation between mRNA and protein coordination (rho 0.4, p < 8e-170) and a weak but significant negative correlation between mRNA-to-protein contribution and mRNA coordination (rho -0.1, p < 1.5e-11) and protein coordination (rho -0.34, p < 5.1e-115), respectively.

Response to reviewer #2

> I thank the authors for addressing most of the points I raised. I still think that (i) the overall conceptual novelty is somewhat modest and that (ii) the paper would greatly benefit from more extensive experimental follow-up. However, I also see that the paper makes a significant contribution to the field and therefore think it can be accepted for publication.

We would like to thank the reviewer for this supportive conclusion regarding our manuscript.

Response to reviewer #3

> I would like to thank the authors for taking the time to write a full response to the review. All of my specific points were appropriately addressed, and I now consider all of the major claims in the manuscript to be adequately supported by the underlying data.

We would like to thank the reviewer for this supportive conclusion regarding our manuscript.

> In reference to the comments I made in the original review about the (then) Supplementary Figures 3 and 4 (first bullet point of page 19/22 in the response to reviewers), I would like to clarify my original comments. First I would like to apologise for my ambiguous and unclear reference to particular panels in these supplementary figures. For the following statement:
>
> 'There also appears to be no correlation in the figure between mRNA coexpression and protein coexpression.'
>
> I was referring to the original Supplementary Figure 3 panel C (second sub-panel from the left), which is now Appendix Figure S5G. The original panel gave no correlation coefficient and by eye the correlation looked much weaker than in the original Figure 2C. However, with the updated analysis this correlation is clearly significant (rho = 0.7, p < 1.8e-5) and is now given in the figure panel.

We thank the reviewer for providing this clarification and are glad that this issue has been successfully resolved.

> For the following statement: 'The enrichment of progulons for low mRNA-to-protein correlations (implying regulation after transcription) also seems to disappear in this figure'
>
> I am unable to recollect which panel I was referring to as the original review was so long ago. There may have been a mistake in the wording of the original sentence. However, after reading the response of the authors and reviewing the relevant figures I am satisfied that the main text faithfully represents the data.

We are glad to hear that this issue has also been resolved. We apologise that the revision process has taken such a long time.

> Overall, I think this manuscript represents a significant advance albeit a fairly modest one in the context of Kustatscher et al., 2019 (Nature Biotech). However, I appreciate the statistical and methodological rigour that has been offered for the revisions and the easy accessibility of Progulon Finder to non-computational biologists: https://www.proteomehd.net/index

We thank the reviewer for these supportive concluding remarks.

Reviewer Appendix #1

ProgulonFinder online output, part 1: Screenshot of a section of the first result file, which contains Random Forest scores.

Uniprot_ACs	RandomForest_score	StDev_of_score	Feature_count	Used_for_positive_training	Training_label	Protein_names	Gene_names	Summed_intensities	Fasta_headers
P24539	0.971554147976302	0.045074114176166	243	Used	Positive	ATP synthase F(0) compl	ATP5F1	275000000000	>sp|P24539|AT5F1_HU
P06576	0.969947955247736	0.051762281215097	291	Used	Positive	ATP synthase subunit bet	ATP5B	2930000000000	>sp|P06576|ATPB_HU
P48047	0.948600606613705	0.067774264663164	254	Used	Positive	ATP synthase subunit O,	ATP5O	407000000000	>sp|P48047|ATPO_HU
O75964	0.935169929946013	0.076178613597738	206	Used	Positive	ATP synthase subunit g,	ATP5L	117000000000	>sp|O75964|ATP5L_HU
P25705;P25705-3	0.929430323436229	0.08007705608862	285	Used	Positive	ATP synthase subunit alp	ATP5A1	2080000000000	>sp|P25705|ATPA_HU
O75947;O75947-2	0.91955129549536	0.079217064647232	220	Used	Positive	ATP synthase subunit d,	ATP5H	202000000000	>sp|O75947|ATP5H_HU
P56385	0.874435612577114	0.10669307121015	183	Used	Positive	ATP synthase subunit e,	ATP5I	53200000000	>sp|P56385|ATP5I_HU
Q96IX5	0.845145478943565	0.109009794264062	196	Used	Positive	Up-regulated during skelet	USMG5	44800000000	>sp|Q96IX5|USMG5_H
P56134;P56134-3	0.830288256687538	0.117107221195647	230	Used	Positive	ATP synthase subunit f, n	ATP5J2	77700000000	>sp|P56134|ATPK_HU
P36542	0.822136549797846	0.123497456480198	258	Used	Positive	ATP synthase subunit gar	ATP5C1	238000000000	>sp|P36542|ATPG_HU
P30049	0.778090123994122	0.124391153972322	209	Used	Positive	ATP synthase subunit del	ATP5D	76700000000	>sp|P30049|ATPD_HU
P03928	0.760536253676829	0.084909651838996	144			ATP synthase protein 8	MT-ATP8	11100000000	>sp|P03928|ATP8_HU
P56378;P56378-2	0.749205228572057	0.062696452892302	144			6.8 kDa mitochondrial prot	MP68	26000000000	>sp|P56378|68MP_HU
P56134-2;P56134-4	0.726858100077079	0.117378880578963	105	Used	Positive	ATP synthase subunit f, n	ATP5J2	2450000000	>sp|P56134-2|ATPK_H
P09669	0.695621667968964	0.06656831526226	168			Cytochrome c oxidase sul	COX6C	57300000000	>sp|P09669|COX6C_HU
P36957	0.695393342691144	0.096562936901584	235			Dihydrolipoyllysine-residue	DLST	132000000000	>sp|P36957|ODO2_HU
P13073	0.690136927579276	0.073698636631615	211			Cytochrome c oxidase sul	COX4I1	201000000000	>sp|P13073|COX41_HU
P20674	0.680788697025906	0.078543851270045	222			Cytochrome c oxidase sul	COX5A	103000000000	>sp|P20674|COX5A_HU
Q99623;Q99623-2	0.680599211117875	0.080972802711647	270			Prohibitin-2	PHB2	679000000000	>sp|Q99623|PHB2_HU
P53597	0.674884407694793	0.06423950540023	241			Succinyl-CoA ligase [ADP	SUCLG1	94400000000	>sp|P53597|SUCA_HU
O96011;O96011-2	0.668651898365658	0.083004685119782	177			Peroxisomal membrane pr	PEX11B	20600000000	>sp|O96011|PX11B_HU
Q9UJZ1;Q9UJZ1-2	0.666150886750753	0.074019605387926	251			Stomatin-like protein 2, m	STOML2	190000000000	>sp|Q9UJZ1|STML2_H
P10176	0.662781641650519	0.076750381139699	75			Cytochrome c oxidase sul	COX8A	110000000	>sp|P10176|COX8A_HU
O43772	0.661965425631348	0.084363824180407	141			Mitochondrial carnitine/acy	SLC25A20	9950000000	>sp|O43772|MCAT_HU
P10606	0.660634959495446	0.060527573710297	212			Cytochrome c oxidase sul	COX5B	40700000000	>sp|P10606|COX5B_HU
P14927	0.65993215530278	0.078144269055503	201			Cytochrome b-c1 complex	UQCRB	47800000000	>sp|P14927|QCR7_HU
P49755	0.657242554478558	0.078977540046941	250			Transmembrane emp24 do	TMED10	253000000000	>sp|P49755|TMEDA_H
O14949	0.655319805582701	0.078321465368724	205			Cytochrome b-c1 complex	UQCRQ	46600000000	>sp|O14949|QCR8_HU
P00403	0.654825115742052	0.073411007890224	227			Cytochrome c oxidase sul	MT-CO2	169000000000	>sp|P00403|COX2_HU
Q9Y6C9	0.652930806906452	0.096156098733094	255			Mitochondrial carrier homo	MTCH2	135000000000	>sp|Q9Y6C9|MTCH2_H
Q00325	0.652585587402865	0.075413072304101	260			Phosphate carrier protein,	SLC25A3	544000000000	>sp|Q00325|MPCP_HU
Q8NCN5	0.650926872893856	0.083411278760276	156			Pyruvate dehydrogenase p	PDPR	13000000000	>sp|Q8NCN5|PDPR_HU

Details for each column are presented on the website after initiating a search, as follows:

- **Uniprot_ACs**: Uniprot accession numbers of the identified proteins and isoforms. If mass spectrometry identifications fit two or more isoforms of a protein, these are separated by a semicolon.
- **RandomForest_score**: The machine learning score reflecting how similar a protein behaves to the uploaded training set. RegulonFinder builds multiple random forests, each comparing the uploaded training proteins against a different set of unrelated proteins. This is the average

score that a protein receives in these different runs. For training proteins, cross-validated scores are reported. The score ranges from 0 to 1. A score of 0.5 or higher indicates that the protein belongs to the same class as the uploaded training proteins, i.e. is part of their regulon.

- **StDev_of_score:** The standard deviation of the score reflects the variation of the score as a result from comparing the uploaded training proteins against different sets of unrelated proteins.
- **Feature_count:** The number of SILAC ratios available for a given protein in ProteomeHD. This is the most important parameter to assess the reliability of the score, i.e. predictions based on more observations are more robust. We report results for proteins with at least 20 feature counts, although care should be taken when following up predictions based on less than 50 counts.
- **Used_for_positive_training:** Indicates whether the protein from the uploaded list of interest was actually used to train the random forest. This may differ from Training_label, because we require proteins to have at least 30 feature counts to be used as training proteins.
- **Training_label:** Indicates whether a protein was on the uploaded list of interest.
- **Protein_names:** Name of the protein as provided by Uniprot.
- **Gene_names:** Name of the gene as provided by Uniprot.
- **Summed_intensities:** The combined peptide intensity of a protein across all experiments in ProteomeHD. This is only included as a useful second dimension for plotting protein scores.
- **Fasta_headers:** Fasta headers of the protein sequence used in ProteomeHD.

Reviewer Appendix #2

The next page is a report generated by the online ProgulonFinder version, after it was executed using 5 random seed proteins

Your "randomexample" report

Method summary

Your proteins of interest were used to mine ProteomeHD v1.0 for co−regulated proteins. The Random Forest implementation of Weka 3.7 was executed in Knime 4.1.3, using 500 trees.
Of your selected training proteins, 5 could be matched to ProteomeHD and these were used as "positive" training data. Of the remaining proteins in ProteomeHD, a representative sample of 1000 were drawn randomly and used as "negative" training data. To avoid using an unbalanced training set, multiple Random Forests were constructed, each using the 5 positive and 5 of the negative training proteins. The average scores and their standard deviation were collected.
Training proteins were extensively cross−validated. This means that, for training data, a total of 5 different Random Forest models were built, each omitting one positive and one negative training protein. The omitted training proteins were then scored by the model for which they did not serve as training proteins.
Predictions are reported for 7813 proteins for which SILAC ratios from at least 30 out of all 294 experiments in ProteomeHD were available. Values in at least 45 features were required for proteins to be used as training data.

Results summary

Cross−validated training data were used to assess performance, yielding an area under the precision−recall curve (AUPRC) of 0.003. (A random classifier would yield an AUPRC of 0.005.)
Random Forest scores are plotted below.

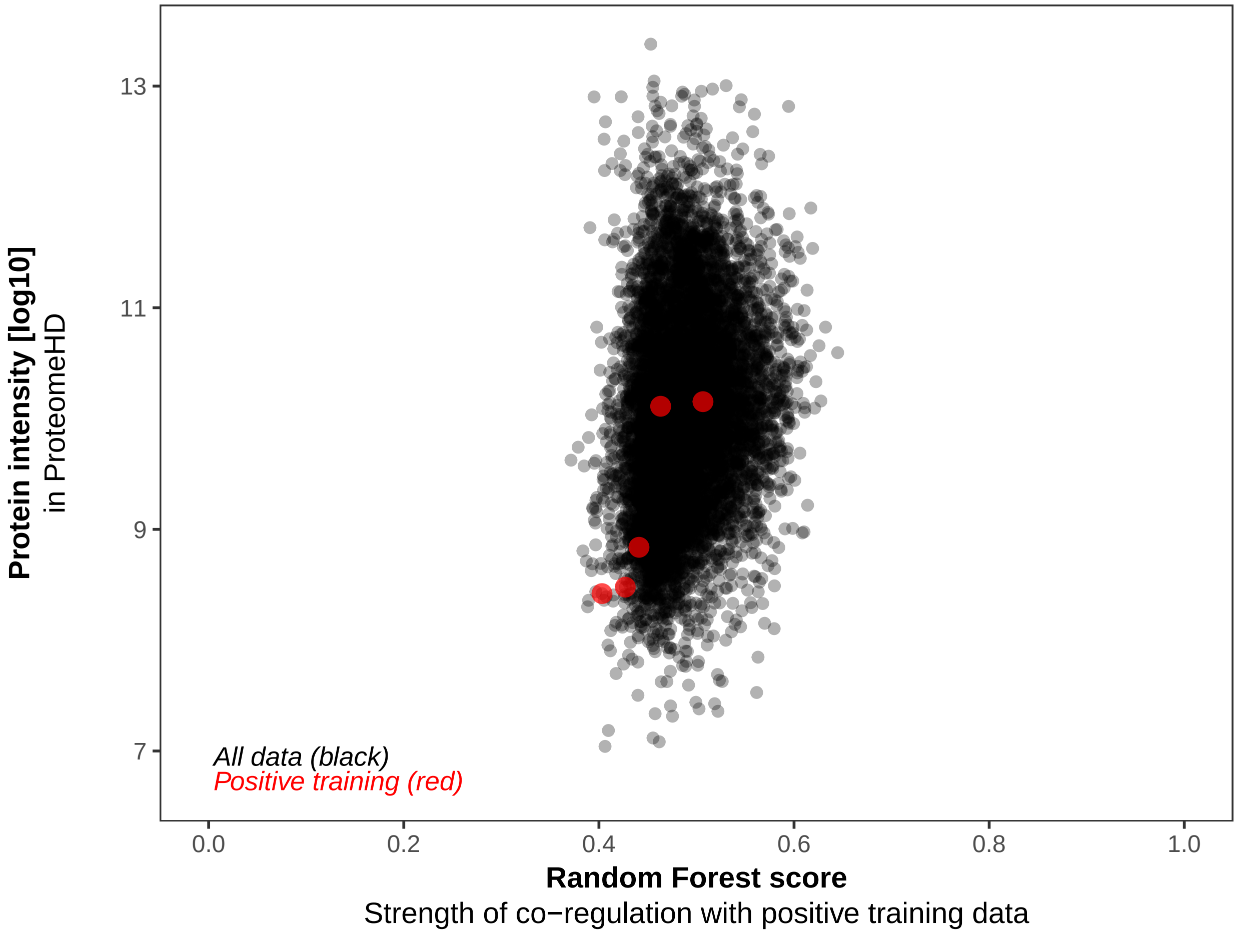

Reviewer Appendix #3

The next page is a report generated by the online ProgulonFinder version, after it was executed using six subunits of the MCM complex

Your "MCM_complex" report

Method summary

Your proteins of interest were used to mine ProteomeHD v1.0 for co−regulated proteins. The Random Forest implementation of Weka 3.7 was executed in Knime 4.1.3, using 500 trees.
Of your selected training proteins, 6 could be matched to ProteomeHD and these were used as "positive" training data. Of the remaining proteins in ProteomeHD, a representative sample of 1002 were drawn randomly and used as "negative" training data. To avoid using an unbalanced training set, multiple Random Forests were constructed, each using the 6 positive and 6 of the negative training proteins. The average scores and their standard deviation were collected.
Training proteins were extensively cross−validated. This means that, for training data, a total of 6 different Random Forest models were built, each omitting one positive and one negative training protein. The omitted training proteins were then scored by the model for which they did not serve as training proteins.
Predictions are reported for 7813 proteins for which SILAC ratios from at least 30 out of all 294 experiments in ProteomeHD were available. Values in at least 45 features were required for proteins to be used as training data.

Results summary

Cross−validated training data were used to assess performance, yielding an area under the precision−recall curve (AUPRC) of 1. (A random classifier would yield an AUPRC of 0.006.)
Random Forest scores are plotted below.

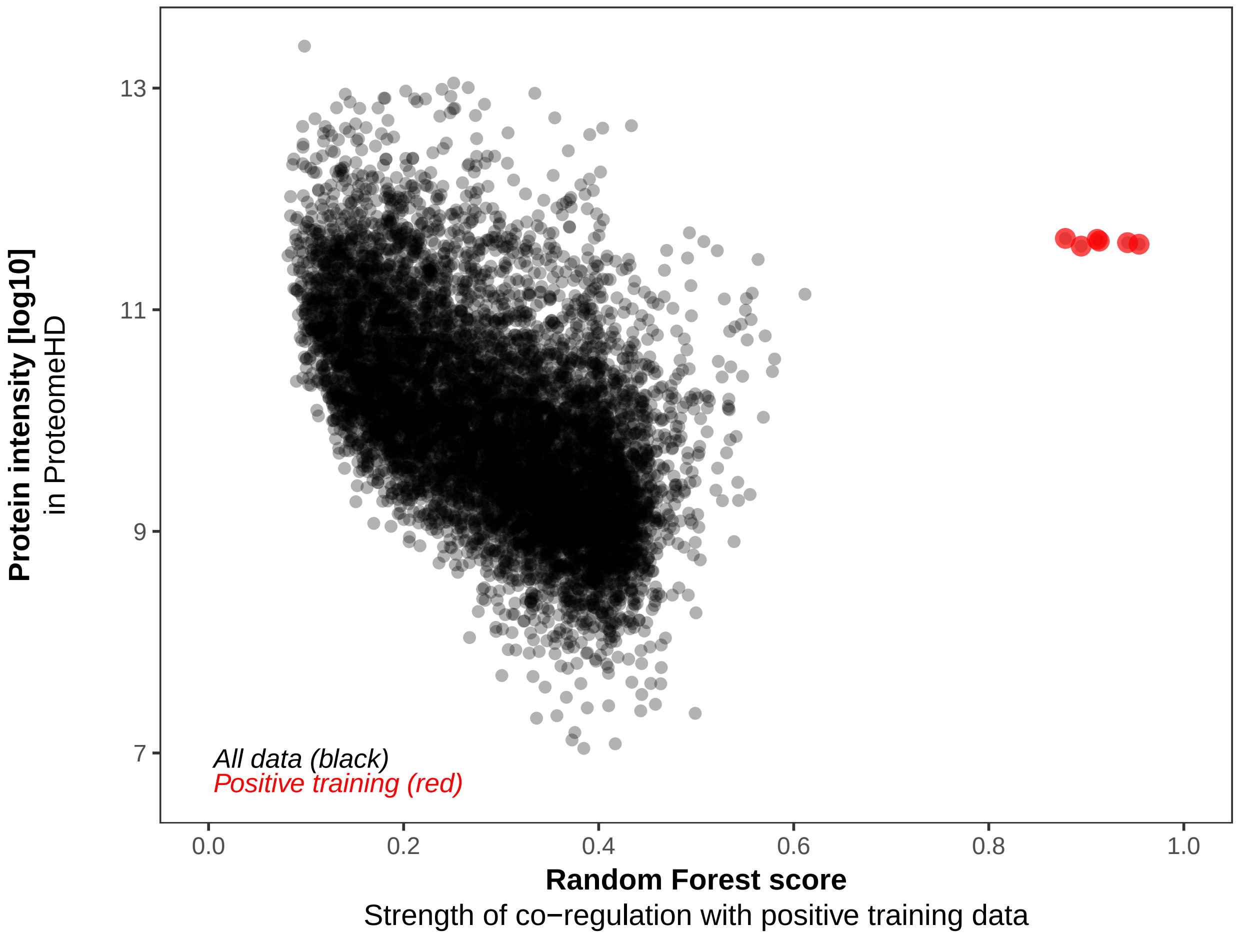

2nd Revision - Editorial Decision 14th Feb 2023

Thank you again for sending us your revised manuscript. We are now satisfied with the modifications made and I am pleased to inform you that your paper has been accepted for publication.

EMBO Press Author Checklist

Corresponding Author Name: **Juri Rappsilber**	
Journal Submitted to: **Molecular Systems Biology**	
Manuscript Number: **MSB-20-9503R**	

USEFUL LINKS FOR COMPLETING THIS FORM
The EMBO Journal - Author Guidelines
EMBO Reports - Author Guidelines
Molecular Systems Biology - Author Guidelines
EMBO Molecular Medicine - Author Guidelines

Reporting Checklist for Life Science Articles (updated January
This checklist is adapted from Materials Design Analysis Reporting (MDAR) Checklist for Authors. MDAR establishes a minimum set of requirements in transparent reporting in the life sciences (see Statement of Task: 10.31222/osf.io/9sm4x). Please follow the journal's guidelines in preparing your manuscript.
Please note that a copy of this checklist will be published alongside your article.

Abridged guidelines for figures
1. Data
The data shown in figures should satisfy the following conditions:
- the data were obtained and processed according to the field's best practice and are presented to reflect the results of the experiments in an accurate and unbiased manner.
- ideally, figure panels should include only measurements that are directly comparable to each other and obtained with the same assay.
- plots include clearly labeled error bars for independent experiments and sample sizes. Unless justified, error bars should not be shown for technical
- if n<5, the individual data points from each experiment should be plotted. Any statistical test employed should be justified.
- Source Data should be included to report the data underlying figures according to the guidelines set out in the authorship guidelines on Data

2. Captions
Each figure caption should contain the following information, for each panel where they are relevant:
- a specification of the experimental system investigated (eg cell line, species name).
- the assay(s) and method(s) used to carry out the reported observations and measurements.
- an explicit mention of the biological and chemical entity(ies) that are being measured.
- an explicit mention of the biological and chemical entity(ies) that are altered/varied/perturbed in a controlled manner.
- the exact sample size (n) for each experimental group/condition, given as a number, not a range;
- a description of the sample collection allowing the reader to understand whether the samples represent technical or biological replicates (including how many animals, litters, cultures, etc.).
- a statement of how many times the experiment shown was independently replicated in the laboratory.
- definitions of statistical methods and measures:

- common tests, such as t-test (please specify whether paired vs. unpaired), simple χ2 tests, Wilcoxon and Mann-Whitney tests, can be unambiguously identified by name only, but more complex techniques should be described in the methods section;

- are tests one-sided or two-sided?
- are there adjustments for multiple comparisons?
- exact statistical test results, e.g., P values = x but not P values < x;
- definition of 'center values' as median or average;
- definition of error bars as s.d. or s.e.m.

Please complete ALL of the questions below.
Select "Not Applicable" only when the requested information is not relevant for your study.

Materials

**Newly Created Materials**	**Information included in the manuscript?**	**In which section is the information available?** (Reagents and Tools Table, Materials and Methods, Figures, Data Availability Section)
New materials and reagents need to be available; do any restrictions apply?	Not Applicable	

**Antibodies**	**Information included in the manuscript?**	**In which section is the information available?** (Reagents and Tools Table, Materials and Methods, Figures, Data Availability Section)
For **antibodies** provide the following information:<br>- Commercial antibodies: RRID (if possible) or supplier name, catalogue number and or/clone number<br>- Non-commercial: RRID or citation	Yes	Methods section

**DNA and RNA sequences**	**Information included in the manuscript?**	**In which section is the information available?** (Reagents and Tools Table, Materials and Methods, Figures, Data Availability Section)
**Short novel DNA or RNA including primers, probes**: provide the sequences.	Yes	Tab "siRNAs used" in data set EV7

**Cell materials**	**Information included in the manuscript?**	**In which section is the information available?** (Reagents and Tools Table, Materials and Methods, Figures, Data Availability Section)
**Cell lines:** Provide species information, strain. Provide accession number in repository **OR** supplier name, catalog number, clone number, and/**OR** RRID.	Yes	Information about cell lines is in paragraph "High-throughput siRNA screening" in the Methods section
**Primary cultures:** Provide species, strain, sex of origin, genetic modification status.	Not Applicable	
Report if the cell lines were recently **authenticated** (e.g., by STR profiling) and tested for mycoplasma contamination.	Not Applicable	

**Experimental animals**	**Information included in the manuscript?**	**In which section is the information available?** (Reagents and Tools Table, Materials and Methods, Figures, Data Availability Section)
**Laboratory animals or Model organisms:** Provide species, strain, sex, age, genetic modification status. Provide accession number in repository **OR** supplier name, catalog number, clone number, **OR** RRID.	Not Applicable	
**Animal observed in or captured from the field:** Provide species, sex, and age where possible**.**	Not Applicable	
Please detail **housing and husbandry conditions**.	Not Applicable	

**Plants and microbes**	**Information included in the manuscript?**	**In which section is the information available?** (Reagents and Tools Table, Materials and Methods, Figures, Data Availability Section)
**Plants:** provide species and strain, ecotype and cultivar where relevant, unique accession number if available, and source (including location for collected wild specimens).	Not Applicable	
**Microbes:** provide species and strain, unique accession number if available, and source.	Not Applicable	

**Human research participants**	**Information included in the manuscript?**	**In which section is the information available?** (Reagents and Tools Table, Materials and Methods, Figures, Data Availability Section)
If collected and within the bounds of privacy constraints report on age, sex and gender or ethnicity for all study participants.	Not Applicable	

**Core facilities**	**Information included in the manuscript?**	**In which section is the information available?** (Reagents and Tools Table, Materials and Methods, Figures, Data Availability Section)
If your work benefited from core facilities, was their service mentioned in the acknowledgments section?	Yes	Acknowledgements

Design

**Study protocol**	**Information included in the manuscript?**	**In which section is the information available?** (Reagents and Tools Table, Materials and Methods, Figures, Data Availability Section)

If study protocol has been **pre-registered, provide DOI in the manuscript**. For clinical trials, provide the trial registration number **OR** cite DOI.	Not Applicable	
Report the **clinical trial registration number** (at ClinicalTrials.gov or equivalent), where applicable.	Not Applicable	

Laboratory protocol	Information included in the manuscript?	In which section is the information available? (Reagents and Tools Table, Materials and Methods, Figures, Data Availability Section)
Provide DOI OR other citation details if **external detailed step-by-step protocols** are available.	Not Applicable	

Experimental study design and statistics	Information included in the manuscript?	In which section is the information available? (Reagents and Tools Table, Materials and Methods, Figures, Data Availability Section)
Include a statement about **sample size** estimate even if no statistical methods were used.	Not Applicable	
Were any steps taken to minimize the effects of subjective bias when allocating animals/samples to treatment (e.g. **randomization procedure**)? If yes, have they been described?	Not Applicable	
Include a statement about **blinding** even if no blinding was done.	Not Applicable	
Describe **inclusion/exclusion criteria** if samples or animals were excluded from the analysis. Were the criteria pre-established?<br><br>If sample or data points were omitted from analysis, report if this was due to attrition or intentional exclusion and provide justification.	Not Applicable	
For every figure, are **statistical tests** justified as appropriate? Do the data meet the assumptions of the tests (e.g., normal distribution)? Describe any methods used to assess it. Is there an estimate of variation within each group of data? Is the variance similar between the groups that are being statistically compared?	Yes	Where necessary, we use Mann Whitney or permutation tests to deal with non-normally distributed data

Sample definition and in-laboratory replication	Information included in the manuscript?	In which section is the information available? (Reagents and Tools Table, Materials and Methods, Figures, Data Availability Section)
In the figure legends: state number of times the experiment was **replicated** in laboratory.	Yes	This is only relevant for the siRNA screen. For this we performed a standard of three independent biological replicates per assay, except in +Aph conditions (6 replicates). This is indicated in the methods section.
In the figure legends: define whether data describe **technical or biological replicates**.	Yes	This is only relevant for the siRNA screen. For this we performed a standard of three independent biological replicates per assay, except in +Aph conditions (6 replicates). This is indicated in the methods section.

Ethics

Ethics	Information included in the manuscript?	In which section is the information available? (Reagents and Tools Table, Materials and Methods, Figures, Data Availability Section)
Studies involving **human participants**: State details of **authority granting ethics approval** (IRB or equivalent committee(s), provide reference number for approval.	Not Applicable	
Studies involving **human participants**: Include a statement confirming that **informed consent** was obtained from all subjects and that the experiments conformed to the principles set out in the WMA Declaration of Helsinki and the Department of Health and Human Services Belmont Report.	Not Applicable	
Studies involving **human participants:** For publication of **patient photos**, include a statement confirming that consent to publish was obtained.	Not Applicable	
Studies involving experimental **animals**: State details of **authority granting ethics approval** (IRB or equivalent committee(s), provide reference number for approval. Include a statement of compliance with ethical regulations.	Not Applicable	
Studies involving **specimen and field samples:** State if relevant **permits** obtained, provide details of authority approving study; if none were required, explain why.	Not Applicable	

Dual Use Research of Concern (DURC)	Information included in the manuscript?	In which section is the information available? (Reagents and Tools Table, Materials and Methods, Figures, Data Availability Section)
Could your study fall under dual use research restrictions? Please check biosecurity documents and list of **select agents and toxins** (CDC): https://www.selectagents.gov/sat/list.htm	Not Applicable	
If you used a select agent, is the security level of the lab appropriate and reported in the manuscript?	Not Applicable	
If a study is subject to dual use research of concern regulations, is the name of the **authority granting approval and reference number** for the regulatory approval provided in the manuscript?	Not Applicable	

Reporting

The MDAR framework recommends adoption of discipline-specific guidelines, established and endorsed through community initiatives. Journals have their own policy about requiring specific guidelines and recommendations to complement MDAR.

Adherence to community standards	Information included in the manuscript?	In which section is the information available? (Reagents and Tools Table, Materials and Methods, Figures, Data Availability Section)
State if relevant guidelines or checklists (e.g., **ICMJE, MIBBI, ARRIVE, PRISMA**) have been followed or provided.	Not Applicable	
For **tumor marker prognostic studies,** we recommend that you follow the **REMARK** reporting guidelines (see link list at top right). See author guidelines, under 'Reporting Guidelines'. Please confirm you have followed these guidelines.	Not Applicable	
For **phase II and III randomized controlled trials**, please refer to the **CONSORT** flow diagram (see link list at top right) and submit the CONSORT checklist (see link list at top right) with your submission. See author guidelines, under 'Reporting Guidelines'. Please confirm you have submitted this list.	Not Applicable	

Data Availability

Data availability	Information included in the manuscript?	In which section is the information available? (Reagents and Tools Table, Materials and Methods, Figures, Data Availability Section)
Have **primary datasets** been deposited according to the journal's guidelines (see 'Data Deposition' section) and the respective accession numbers provided in the Data Availability Section?	Not Applicable	
Were **human clinical and genomic datasets** deposited in a public access-controlled repository in accordance to ethical obligations to the patients and to the applicable consent agreement?	Not Applicable	
Are **computational models** that are central and integral to a study available without restrictions in a machine-readable form? Were the relevant accession numbers or links provided?	Yes	Data availability statement and methods section
If publicly available data were reused, provide the respective **data citations in the reference list**.	Yes	Publicly available data were used (ProteomeHD) and appropriately referenced.
